# Polyphenols for the Prevention or Management of Preeclampsia: A Systematic Review and Meta‐Analysis

**DOI:** 10.1111/1471-0528.18106

**Published:** 2025-03-03

**Authors:** Phi‐Yen Nguyen, Ben Sanderson, Maureen Makama, Kate Mills, Anne Ammerdorffer, A. Metin Gülmezoglu, Joshua P. Vogel, Annie R. A. McDougall

**Affiliations:** ^1^ Maternal, Child and Adolescent Health Program Burnet Institute Melbourne Australia; ^2^ Concept Foundation Geneva Switzerland

**Keywords:** cranberry extract, Danshen, ECGC, epigallocatechin gallate, polyphenols, preeclampsia, pregnancy, raspberry extract, resveratrol, *Salvia miltiorrhiza*

## Abstract

**Objectives:**

To evaluate the effects of polyphenol‐containing products during pregnancy on preeclampsia‐related maternal and neonatal outcomes.

**Design:**

Systematic review and meta‐analysis.

**Setting:**

Nine databases and one trial registry, from inception to August 11th, 2023.

**Population/Sample:**

Randomised controlled trials where women received polyphenolic‐containing products (as standardised extracts or dietary supplements) compared to placebo or standard care.

**Methods:**

All review stages were conducted by two independent reviewers. Random‐effects meta‐analysis with the Hartung–Knapp–Sidik–Jonkman method using a framework for studies with few events.

**Main Outcome Measures:**

Clinical outcomes combining the core outcome set for preeclampsia and WHO's priority outcomes.

**Results:**

Fourteen trials investigating six candidates were included. In women with preeclampsia, the addition of epigallocatechin gallate (EGCG) to nifedipine may reduce the time needed to achieve blood pressure control (mean difference (MD) = −14.10 min, 95% CI −18.46 to −9.74) and increase the time to the next hypertensive crisis (MD = 3.10 h, 95% CI 2.35 to 3.85) compared to nifedipine alone (1 trial, 349 women; low certainty). Similarly, the addition of resveratrol to nifedipine may reduce the time needed to achieve blood pressure control (MD = −15.50 min, 95% CI −19.83 to −11.17) and increase the time to the next hypertensive crisis (MD = 2.50 h, 95% CI 2.09 to 2.91) (1 trial, 349 women; low certainty). No differences were observed for other outcomes or candidates (*Salvia miltiorrhiza, Bryophyllum pinnatum* , raspberry and cranberry extracts).

**Conclusions:**

ECGC and resveratrol supplements have been investigated for potential effects in managing clinical signs and symptoms of preeclampsia; however, evidence on the clinical and adverse effects of polyphenols is limited and uncertain.

## 1 Introduction

Globally, preeclampsia and other hypertensive disorders of pregnancy (HDP) are responsible for 14% of maternal deaths annually [1]. Preeclampsia increases the risk of several maternal and birth complications [2, 3]. Although the aetiology of preeclampsia is poorly understood, it involves abnormal placentation, in which an increased secretion of inflammatory cytokines, soluble fms‐like tyrosine kinase‐1 (sFlt‐1) and endoglin [4] leads to endothelial dysfunction, placental inflammation and maternal vascular dysfunction [5, 6].

There are currently few therapeutic options for the prevention and management of preeclampsia. The World Health Organization (WHO) and the International Federation of Obstetrics and Gynecology (FIGO) recommend low‐dose aspirin and calcium supplementation for the prevention of preeclampsia [7, 8]. Antihypertensives are recommended for the management of hypertension during pregnancy [7, 8, 9, 10, 11]. Magnesium sulphate is effective for the prevention and treatment of eclampsia [12]. However, the adoption of magnesium sulphate has been impeded in many settings by the complexities of administering the full regimen, supply chain issues and persisting beliefs against its adoption [13]. Therefore, there is a clear need to identify and evaluate novel, promising preventive and treatment options for preeclampsia.

In a medicines R&D pipeline developed as part of the Accelerating Innovation for Mothers (AIM) project [14], polyphenols were found to be one of the most commonly investigated subclasses of medicines for the prevention and treatment of preeclampsia, with 15 unique candidates in preclinical or clinical development between 2000 and 2021. Polyphenols are a chemical class comprising over 8000 compounds [15, 16], present in a wide range of food, beverages and dietary supplements [15, 16]. In animal models of hypertensive disorders, polyphenolic compounds demonstrated antihypertensive effects by inhibiting inflammatory cytokines, increasing endothelial nitric oxide production and correcting imbalances in vascular angiogenic growth factors [17, 18, 19].

Despite the growing interest in polyphenols, their clinical effectiveness for pregnant women at risk of or diagnosed with preeclampsia remains unclear. A 2021 comprehensive review of polyphenols focused primarily on preclinical evidence [17]. Previous reviews of clinical evidence only focused on polyphenol‐rich foods [20, 21]; the effects of extracts or supplements, which usually contain higher concentrations of specific polyphenols, have not been systematically evaluated.

In this review, we aim to synthesise the evidence on the effects of supplements and standardised extracts containing polyphenols during pregnancy on preeclampsia and other maternal, foetal and neonatal outcomes.

## 2 Methods

This review was registered on PROSPERO (CRD42023403442) and reported according to the PRISMA guideline [22]. Minor deviations from the protocol are outlined in File S1.

### 2.1 Eligibility Criteria

Eligible interventions include any type of products containing polyphenols or their derivatives, in the form of a standardised plant extract or dietary supplement (See examples in File S2). For the purpose of this review, polyphenols were defined as molecules that contain at least two phenyl rings and one or more hydroxyl substituents [23, 24]. Polyphenols included in mixtures of traditional or herbal supplements, such as Traditional Chinese Medicine or Ayurveda formulations, were not eligible.

Eligible studies were randomised, controlled clinical trials comparing polyphenol‐containing products to placebo, no treatment, usual care or standard treatment. Trials, where a polyphenol supplementation was used in combination with other supplements, were only included if the control group received the same supplement minus the polyphenol. Trials that included pregnant women who received the polyphenol‐containing product during pregnancy (regardless of the parity or plurality) were eligible. Eligible trials were considered treatment trials if included women who were already diagnosed with preeclampsia. Prevention trials were defined as those including women who had not been diagnosed with preeclampsia at recruitment (regardless of included women's risk of preeclampsia). Trials that included women in the preconception or postpartum period, women undergoing treatment for infertility or studies where women did not become pregnant throughout the study were not eligible. Observational studies, reviews, case reports, case series, letters and commentaries, book chapters and conference abstracts were not eligible. Studies were not excluded on the basis of whether our review outcomes were reported or not.

### 2.2 Information Sources

Nine databases were searched (MEDLINE, Embase, EMCare, Maternity and Infant Care Database (MIC/MIDIRS), and Allied and Complementary Medicine Database (AMED) via Ovid; The Cochrane Library for Cochrane Reviews; CINAHL via EBSCOhost; Global Index Medicus; and the ICTRP trial registry), from inception to date of search (last search was on 11 August 2023), with no restriction on date or language. In addition, we searched for eligible studies from the AIM database of candidate medicines for priority maternal conditions [14].

### 2.3 Search Strategy

The search strategy was developed in consultation with a librarian, which combined keywords and subject headings related to maternal and neonatal outcomes, classes of polyphenols [15, 16] and polyphenol‐rich plants that have been evaluated in preclinical studies for their potential role in treating preeclampsia [17, 25] (File S3). References from systematic reviews of eligible interventions were also screened for potentially eligible studies.

### 2.4 Study Selection

Two review authors (P‐YN and BS) independently screened the titles and abstracts, followed by their full texts, using Covidence [26]. Discrepancies were resolved by consensus or by consulting a third reviewer (JPV or AMD).

### 2.5 Trial Integrity Assessment

Two authors (P‐YN and BS) independently assessed the included studies for research integrity and their adherence to good clinical practices, using an adapted tool [27]. Trials with identified issues across multiple categories were assigned the status of ‘Awaiting classification’ and were not included for further analysis until a satisfactory response was received from authors.

### 2.6 Data Extraction

Two authors (P‐YN and BS) independently extracted data using a standardised data extraction form in Covidence. Discrepancies were resolved through consensus. The following characteristics were extracted from each included study: (a) study characteristics: location, funding and conflicts of interest; (b) participants: gestational age at baseline and initiation of treatment and risk factors for or diagnosis of preeclampsia; (c) interventions and comparators: name of polyphenol or plant product, mode of administration, dosage, frequency and duration of administration and any co‐intervention(s); and (d) outcomes: clinical definition and timing of measurement.

The primary outcome of prevention trials (involving women at risk of preeclampsia) was the incidence of preeclampsia. For treatment trials (involving women with diagnosed preeclampsia), the primary outcome was maternal death, or progression to severe preeclampsia, eclampsia or HELLP syndrome. The complete list of primary and secondary outcomes (File S4) aligns with the core outcome set for preeclampsia research [28] and the priority outcomes used in WHO's guidelines [10].

For each outcome, numbers of events were recorded for dichotomous outcomes; means and standard deviations were recorded for continuous outcomes. Missing standard deviations were imputed using other available statistics. We attempted to contact the corresponding authors via email to seek any missing data for up to three times.

### 2.7 Risk of Bias and Certainty of Evidence Assessment

Two authors independently assessed the risk of bias in included studies using the RoB 2.0 tool for randomised trials (RoB 2.0) [29]. Disagreements were resolved through consensus. The certainty of evidence was assessed following the GRADE framework [30].

### 2.8 Data Synthesis

Intervention effects were estimated using risk ratios for dichotomous outcomes and mean differences for continuous outcomes. Separate meta‐analyses were conducted for each polyphenol compound and outcome [31]. We followed the framework proposed by Schulz and colleagues for analysis and reporting of outcomes with few studies [32]. We implemented a random‐effects model. The summary effect estimate was calculated using the Mantel–Haenszel method for dichotomous data and the inverse variance method for continuous data. The 95% confidence interval was estimated using the Hartung–Knapp–Sidik–Jonkman (HKSJ) method with an ad hoc variance correction [33, 34, 35]. If the 95% confidence interval of the HKSJ effect estimate was larger than the union of the confidence intervals of all included studies, the effect estimate was considered noninformative. In such cases, we determined the general direction of the effect based on the largest studies contributing 80% of weight to the meta‐analysis [32]. Meta‐analyses were conducted using the *meta* package (v6.2–1) in R (v4.2.3). For studies that could not be meta‐analysed, we presented a narrative summary of the findings and the results as reported by the authors.

### 2.9 Funding

This project was funded by the Bill & Melinda Gates Foundation (INV‐038938), Perpetual's IMPACT Philanthropy Application Program (IPAP2022/0475; 22/06/2022) and the Burnet Institute (Z3101). JPV is supported by an Australian National Health and Medical Research Council (NHMRC) Investigator Grant (GNT1194248). The funder organisations had no role in the design and conduct of the study; collection, management, analysis and interpretation of the data; preparation, review or approval of the manuscript; and decision to submit the manuscript for publication.

### 2.10 Patient and Public Involvement

As this was a systematic review, patients and the public were not involved in the design, conduct, reporting or dissemination plans of our research.

## 3 Results

### 3.1 Study Selection

We retrieved 9947 records, of which 7553 remained after removal of duplicates. We excluded 7391 abstracts and subsequently 118 full texts during screening (File S5). Eight reports were ongoing trials or protocols without any published results (File S6). One of these ongoing trials is specifically recruiting women with preexisting preeclampsia and aims to investigate the effects of broccoli sprout extract compared to placebo in managing early‐onset preeclampsia. Following the trial integrity assessment, 16 reports were classified as ‘awaiting classification’ due to unclear descriptions of randomisation and recruitment methods (File S7). We thus included 21 trials, of which 14 reported on our review outcomes [36, 37, 38, 39, 40, 41, 42, 43, 44, 45, 46, 47, 48] (File S8). The other seven eligible trials did not report data on any review outcome [49, 50, 51, 52, 53, 54, 55] (File S9). The Cohen's Kappa statistic is 0.56 for title/abstract screening and 0.63 for full‐text screening.

### 3.2 Study Characteristics

The 14 included trials were published between 2000 and 2018 (File S10). Most were conducted in China (9 trials, 64%) [36, 37, 38, 39, 41, 42, 45, 47, 48]; the remaining five trials were conducted in four high‐income countries (Italy, Switzerland, Australia and the United States of America) [40, 43, 44, 46]. Five trials (36%) recruited pregnant women with diagnosed preeclampsia, which we defined as treatment trials [36, 38, 41, 42, 48]. Six trials (43%) recruited pregnant women with some health risk factors (e.g., being overweight, gestational diabetes, intrauterine growth retardation or nulliparity) [37, 40, 43, 45, 47], and three trials (21%) recruited women with uncomplicated pregnancies [39, 44, 46], which we defined as prevention trials. Among treatment trials, one specified that treatment was initiated between 26 and 34 gestational weeks [41]; the remaining trials only reported the gestational age at baseline, which ranged from 33 to 38 weeks [36, 38, 42, 48]. Among the nine prevention trials, two trials initiated treatment between 12 and 16 gestational weeks [25, 46]; three trials initiated treatment in the third trimester [37, 44, 47]; and four trials only reported the gestational age at baseline, which ranged from 24 to 41 weeks [40, 43, 45].

No trials were reported on these outcomes: maternal mortality, preeclampsia disease progression/severity, prolonged gestation in women with preeclampsia, eclampsia, preterm birth or severe morbidities (e.g., thrombocytopenia, renal or liver complications). Neonatal outcomes without data included foetal loss (stillbirth, miscarriage or intrapartum death), small‐for‐gestational age and congenital defects. There were no data available on important biomarkers for preeclampsia, namely, soluble fms‐like tyrosine kinase 1 (sFlt‐1), pregnancy‐associated plasma protein A (PAPP‐A), placental growth factor (PIGF) or soluble endoglin (sEng).

All trials have either some concerns (48%) or a high risk of bias (52%) (File S11).

### 3.3 Overview of Interventions

The polyphenol‐containing products investigated in the 14 included trials were as follows: Danshen ( *Salvia miltiorrhiza* ) injection [37, 38, 39, 41, 45, 48], epigallocatechin gallate (ECGC) supplement (commonly found in green tea) [42, 47], resveratrol supplement [36, 40] (commonly found in red grapes), *Bryophyllum pinnatum* extract [43], cranberry ( *Vaccinium macrocarpon* ) extract [46] and raspberry ( *Rubus idaeus* ) extract [44] (File S10). Danshen is the dried rhizome of *Salvia miltiorrhiza* or red sage, commonly used in Chinese traditional medicine, which contains phenolic acids (including salvianolic acids A and B and rosmarinic acid) [56]. ECGC is the main flavonoid compound in green tea extract [57]. Resveratrol is an antioxidant from the stilbenoid class, found abundantly in whole grapes (Vitis vinifera) and grape products [58]. *B. pinnatum* is a succulent plant native to Madagascar; its leaves contain several flavonoids and anthocyanins [59]. Raspberry has a high content of anthocyanins and ellagitannins, two major groups of polyphenols [60]. Cranberry contains a diverse profile of polyphenols, including flavonols, proanthocyanidins, anthocyanins and phenolic acid derivatives [61].

### 3.4 Synthesis of Results

#### 3.4.1 Danshen ( *Salvia miltiorrhiza* )

Six trials were reported on Danshen injection; three in women with risk factors for preeclampsia and three in women with diagnosed preeclampsia. All six trials used a similar method of preparing the Danshen injection, that is, by mixing Danshen extract (concentration and dosage not specified) with 250–500 mL of 5% glucose solution or dextran solution for infusion once daily. However, the timing of initiation varied across trials, ranging from 14 to 37 weeks of gestation, and the duration of treatment ranged from 3 to 10 days.

Three trials (4967 women) in China evaluated the effectiveness of intravenous (IV) injections of Danshen extract in women without preeclampsia but with at least one risk factor (e.g., liver diseases, intrauterine growth restriction and age above 40) [37, 39, 45]. One trial compared the combination of Danshen injection with oral ursodeoxycholic acid against ursodeoxycholic acid alone [37]. A second trial compared the combination of Danshen injection with oral vitamin C and vitamin E tablets against vitamin C and vitamin E alone [39]. In the third trial, the intervention group received Danshen injection, while the control group received no intervention [45]. Evidence on the effects of Danshen injection on all reported outcomes was very uncertain (Table 1).

**TABLE 1:** Summary of results | Danshen ( *Salvia miltiorrhiza* ).

Outcome	Results from meta‐analysis^a^	No. of patients/RCT	Reference	Quality of evidence (GRADE)
Women without preeclampsia				
Hypertensive disorders of pregnancy Includes chronic hypertension, gestational hypertension and preeclampsia	RR 0.85 (0.63 to 1.15) 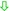	4814 / 1	[37]	⊖⊕⊕⊖ LOW
Birthweight (g)	MD 415.32 (38.40 to 792.24), *I* ^2^=0% 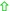 Summary MD is noninformative (95% CI was too wide). Effects were directed towards higher birth weight in the intervention group.	153 / 2	[35, 43]	⊖⊕⊖⊕ VERY LOW
High Apgar score 5‐min Apgar score > 7	RR 1.00 (0.92 to 1.09)	128 / 1	[35]	⊖⊕⊖⊖ VERY LOW
Foetal distress Foetus experiencing altered heart rates, altered movement or showing signs of oxygen deprivation before or during labour; or otherwise as defined by authors	RR 0.92 (0.45 to 1.89) 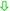	128 / 1	[35]	⊖⊕⊖⊖ VERY LOW
Meconium staining of amniotic fluid	RR 0.86 (0.38 to 1.98) 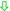	128 / 1	[35]	⊖⊕⊖⊖ VERY LOW
Women with preexisting preeclampsia or other HDPs				
Elevated transaminases (ALT/AST)	RR 0.95 (0.39 to 2.34) 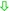	69 / 1	[39]	⊖⊕⊕⊖ LOW
Placental abruption	RR 3.27 (0.14 to 77.51) 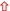	69 / 1	[39]	⊖⊕⊕⊖ LOW
Postpartum haemorrhage Blood loss of > 500 mL within 24 h after childbirth; or otherwise as defined by authors	RR 0.36 (0.04 to 3.33) 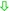	69 / 1	[39]	⊖⊕⊕⊖ LOW
Birthweight (g)	MD 19.10 (−44.40 to 82.60) 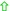	69 / 1	[39]	⊖⊕⊕⊖ LOW
Neonatal asphyxia	RR 0.31 (0.10 to 0.95) 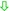	69 / 1	[39]	⊖⊕⊕⊖ VERY LOW
Neonatal death	Zero events in both groups	69 / 1	[39]	—
Prostaglandin I_2_ (PGI_2_) (pg/L)	MD 49.65 (−275.75 to 375.06), *I* ^2^=98.9% 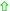 Summary MD is noninformative (95% CI was too wide). Effects were directed towards higher PGI_2_ levels in the intervention group.	758 / 2	[36, 46]	⊖⊖⊕⊖ VERY LOW
Endothelin‐1 (ET‐1) (ng/L)	MD‐12.96 (−77.06 to 51.14), *I* ^2^=98.11% 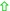 Summary MD is noninformative (95% CI was too wide). Effects were directed towards lower ET‐1 levels in the intervention group.	758 / 2	[36, 46]	⊖⊖⊕⊖ VERY LOW

Abbreviations: ALT, alanine transaminase; AST, aspartate transaminase; HDP, hypertensive disorders of pregnancy; MD, mean difference; RCT, randomised controlled trial; RR: risk ratio.

^a^ Legend: 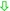 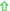 Effect in favourable direction (health benefit). 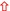 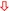 Effect in unfavourable direction (health risk/harm). Forest plots are available in File S13. Heterogeneity statistics *I* ^2^ is only provided if a meta‐analysis was conducted; otherwise, results are from single trials.

Three trials (827 women) in China evaluated the effectiveness of intravenous (IV) injections of Danshen extract in women with diagnosed preeclampsia [38, 41, 48]. Two trials compared the combination of Danshen injection plus IV magnesium sulphate against IV magnesium sulphate alone [38, 41]. One trial compared the combination of Danshen injection plus IV magnesium sulphate and oral nifedipine against IV magnesium sulphate and oral nifedipine only [48]. Low‐certainty evidence from one trial (69 women) [41] suggests that Danshen may not reduce the risk of elevated transaminases (RR 0.95, 95% CI 0.39 to 2.34), placental abruption (RR 3.27, 95% CI 0.14 to 77.51), postpartum haemorrhage (RR 0.36, 95% CI 0.04 to 3.33) or birthweight (MD 19.10 g, 95% CI –44.40 to 82.60). Evidence for other neonatal outcomes (e.g., neonatal asphyxia and neonatal death) and biomarkers of preeclampsia pathogenesis (serum PGI_2_ level and ET‐1) were very uncertain (Table 1).

#### 3.4.2 Epigallocatechin Gallate (ECGC)

One trial of 404 women in China examined ECGC for the prevention of GDM [47]. Preeclampsia was not reported as an outcome. Evidence for reported neonatal outcomes was very uncertain (Table 2).

**TABLE 2:** Summary of results | Epigallocatechin gallate extract.

Outcome	Results from meta‐analysis^a^	No. of patients/RCT	Reference	Quality of evidence (GRADE)
Women without preeclampsia				
Apgar score [5‐min] (points)	MD 1.40 (1.28 to 1.52) 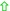	326 / 1	[45]	⊖⊕⊖⊖ VERY LOW
Low birthweight Birthweight < 2500 g; or otherwise as defined by authors	RR 0.27 (0.14 to 0.52) 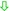	326 / 1	[45]	⊖⊕⊖⊖ VERY LOW
Macrosomia	RR 0.32 (0.09 to 1.18) 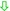	326 / 1	[45]	⊖⊕⊖⊖ VERY LOW
Gestational age at birth (weeks)	MD‐0.70 (−0.95 to −0.45) 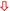	326 / 1	[45]	⊖⊕⊖⊖ VERY LOW
Neonatal hypoglycaemia	RR 0.31 (0.10 to 0.95)) 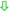	326 / 1	[45]	⊖⊕⊖⊖ VERY LOW
Neonatal respiratory distress	RR 0.51 (0.12 to 2.10) 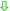	326 / 1	[45]	⊖⊕⊖⊖ VERY LOW
Women with preexisting preeclampsia or other HDPs				
Number of doses needed to control blood pressure	Among the intervention group, the most frequent number of doses is two (36%), three (39%) and one (28%). Among the control group, the most frequent number of doses is two (37%), three (35%) and one (17%).	304 / 1	[40]	—
Time needed to control blood pressure (min)	MD‐14.10 (−18.46 to −9.74) 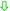	304 / 1	[40]	⊖⊕⊕⊖ LOW
Time to next hypertensive crisis (hr)	MD 3.10 (2.35 to 3.85) 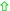	304 / 1	[40]	⊖⊕⊕⊖ LOW
High Apgar score 5‐min Apgar score > 6	RR 1.03 (0.93 to 1.14) 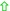	304 / 1	[40]	⊖⊕⊕⊖ LOW
Birthweight (g)	MD‐160.00 (−290.79 to −29.21) 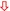	304 / 1	[40]	⊖⊕⊕⊖ LOW

Abbreviations: ECGC, epigallocatechin gallate; MD, mean difference; RCT, randomised controlled trial; RR: risk ratio.

^a^ Legend: 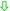 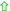 Effect in favourable direction (health benefit). 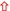 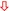 Effect in unfavourable direction (health risk/harm). Forest plots are available in File S13. Heterogeneity statistics *I* ^2^ is only provided if a meta‐analysis was conducted; otherwise, results are from single trials.

In a trial of 304 women with preeclampsia [42] in China, compared to nifedipine alone, the addition of ECGC supplement to nifedipine may reduce the time needed to achieve blood pressure control (MD‐14.10 min, 95% CI −18.46 to −9.74) and may increase the time to the next hypertensive crisis (MD = 3.10 h, 95% CI 2.35 to 3.85, low certainty). The addition of ECGC may make no difference to the risk of Apgar scores > 6 (RR = 1.03, 95% CI 0.93 to 1.14, low certainty), but may result in lower birthweight (MD = −160.00 g, 95% CI −290.79 to −29.21, low certainty). Side effects (e.g., headache, dizziness, nausea and vomiting) were reported, but their frequencies may not be different between the ECGC and control groups (low‐certainty evidence) (File S12).

### 3.5 Resveratrol

In an Italian trial of 69 overweight women without preeclampsia [40], compared to myo‐inositol and D‐chiro‐inositol alone, the effects of adding transresveratrol (a form of resveratrol with higher bioavailability) on systolic and diastolic blood pressure were uncertain (Table 3).

**TABLE 3:** Summary of results | Resveratrol and *Bryophyllum pinnatum* extract.

Outcome	Results from meta‐analysis^a^	No. of patients/RCT	Reference	Quality of evidence (GRADE)
Resveratrol supplement				
Women without preeclampsia				
Systolic blood pressure (mmHg)	At 30 days postintervention: MD 0.66 (−2.23 to 3.55) 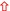 At 60 days postintervention: MD‐1.16 (−4.08 to 1.76) 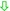	69/1	[38]	⊖⊕⊕⊖ LOW
Diastolic blood pressure (mmHg)	At 30 days postintervention: MD 2.02 (−1.11 to 5.15) 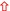 At 60 days postintervention: MD 1.88 (−1.14 to 4.90) 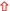	69/1	[38]	⊖⊕⊕⊖ LOW
Women with preexisting preeclampsia or other HDPs				
Number of doses needed to control blood pressure	Data not provided	349/1	[34]	—
Time needed to control blood pressure (min)	MD‐15.50 (−19.83 to −11.17) 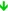	349/1	[34]	⊖⊕⊕⊖ LOW
Time to next hypertensive crisis (hr)	MD 2.50 (2.09 to 2.91) 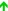	349/1	[34]	⊖⊕⊕⊖ LOW
High Apgar score 5‐min Apgar score > 6	RR 1.03 (0.93 to 1.14) 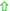	349/1	[34]	⊖⊕⊕⊖ LOW
Birthweight (g)	MD‐120.0 (−244.90 to 4.90) 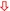	349/1	[34]	⊖⊕⊕⊖ LOW
*Bryophyllum pinnatum* extract				
Women without preeclampsia				
Systolic blood pressure (mmHg)	MD 1.00 (−6.97 to 8.97) 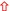	27/1	[41]	⊖⊕⊖⊖ VERY LOW
Diastolic blood pressure (mmHg)	MD‐1.00 (−7.16 to 5.16) 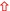	27/1	[41]	⊖⊕⊖⊖ VERY LOW
Apgar score [5‐min] (points)	MD 4.45 (−53.36 to 62.26), *I* ^2^=99.81% 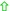 Summary MD is noninformative (95% CI was too wide). No directed effect was observed.	68/2	[41]	⊖⊖⊖⊖ VERY LOW
Birthweight (g)	MD 63.28 (−2932.55 to 3059.10), *I* ^2^=58.3% 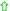 Summary RR is noninformative (95% CI was too wide). No directed effect was observed.	68/2	[41]	⊖⊖⊖⊖ VERY LOW
Gestational age at birth (weeks)	MD 1.13 (−4.25 to 6.50), *I* ^2^=0% 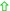 Summary RR is noninformative (95% CI was too wide). No directed effect was observed.	51/2	[41]	⊖⊖⊖⊖ VERY LOW
Preterm birth Birth before 37 weeks of pregnancy; or otherwise as defined by authors	RR 1.12 (0.64 to 1.97) 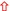	26/1	[41]	⊖⊕⊖⊖ VERY LOW
Neonatal intensive care unit admission	RR 1.05 (0.24 to 4.61) 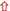	41/1	[41]	⊖⊕⊖⊖ VERY LOW

Abbreviations: MD: mean difference; RCT: randomised controlled trial; RR: risk ratio.

^a^ Legend: 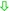 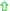 Effect in favourable direction (health benefit). 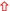 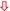 Effect in unfavourable direction (health risk/harm). Forest plots are available in File S13. Heterogeneity statistics *I* ^2^ is only provided if a meta‐analysis was conducted; otherwise, results are from single trials.

A trial of 349 women with severe preeclampsia in China [36] compared nifedipine alone to resveratrol plus nifedipine and found that the addition of resveratrol may reduce the time needed to achieve control of blood pressure (MD = −15.50 min, 95% CI −19.83 to −11.17, low certainty) and may increase the time to the next hypertensive crisis (MD = 2.50 h, 95% CI 2.09 to 2.91, low certainty). The addition of resveratrol may make no difference to birthweight (MD = −120 g, 95% CI −224.9 to 4.90, low certainty) or Apgar scores (RR = 1.03, 95% CI 0.93 to 1.14, low certainty). Side effects were reported, but their frequencies may not be different between the resveratrol and control groups (low‐certainty evidence) (File S12).

#### 3.5.1 Bryophyllum pinnatum

Two trials in Switzerland [43] measured the clinical efficacy of *B. pinnatum* as a tocolytic agent in women at risk of preterm contractions without preeclampsia, one comparing to placebo and one comparing to nifedipine—both were stopped prematurely. Evidence was very uncertain for blood pressures, neonatal outcomes (e.g., Apgar scores, birthweight and preterm birth) (Table 3) and maternal side effects (File S12) [43].

### 3.6 Raspberry Leaf Extract ( *Rubus idaeus* )

One trial of 192 women in Australia evaluated the effect of raspberry leaf extract in nulliparous pregnant women without preeclampsia, compared to placebo [44]. Raspberry extract may not reduce the risk of NICU admission (RR1.43, 95% CI 0.57 to 3.60; low certainty). Evidence was very uncertain about the effects of raspberry extract on the risk of preeclampsia, postpartum haemorrhage and other neonatal outcomes (Table 4). Side effects were reported, but the evidence was very uncertain (File S12).

**TABLE 4:** Summary of results | Raspberry ( *Rubus idaeus* ) and cranberry ( *Vaccinium macrocarpon* ) extract.

Outcome	Results from meta‐analysis^a^	No. of patients/RCT	Reference	Quality of evidence (GRADE)
Raspberry ( *Rubus idaeus* ) extract				
Women without preeclampsia				
Preeclampsia: Onset of high blood pressure and protein, with or without signs of organ damage; or otherwise as defined by authors	RR 2.00 (0.71 to 5.63) 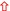	192/1	[42]	⊖⊕⊖⊖ VERY LOW
Diastolic blood pressure (mmHg)	Data not provided	Not reported/1	[42]	—
Postpartum haemorrhage: Blood loss of > 500 mL within 24 h after childbirth; or otherwise as defined by authors	Incidence was 9 in intervention group and 12 in control group	Not reported/1	[42]	—
Amount of vaginal blood loss (mL)	MD‐11.70 (−141.49 to 118.09)^b^ 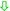	148/1	[42]	⊖⊕⊖⊖ VERY LOW
Apgar score [5‐min] (points)	MD‐0.14 (−0.32 to 0.04) 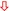	192/1	[42]	⊖⊕⊖⊖ VERY LOW
Birthweight (g)	MD‐44.27 (−161.47 to 72.93)^b^ 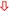	192/1	[42]	⊖⊕⊖⊖ VERY LOW
Low birthweight Birthweight < 2500 g; or otherwise as defined by authors	RR 0.24 (0.01 to 5.08)^c^ 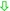	17/1	[42]	⊖⊕⊖⊖ VERY LOW
Gestational age at birth (weeks)	MD‐0.11 (−0.44 to 0.22)^a^ 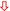	192/1	[42]	⊖⊕⊖⊖ VERY LOW
Preterm birth Birth before 37 weeks of pregnancy; or otherwise as defined by authors	RR 0.50 (0.05 to 5.30)^c^ 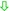	17/1	[42]	⊖⊕⊖⊖ VERY LOW
Meconium staining of amniotic fluid	RR 0.47 (0.10 to 2.10)^b^ 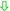	17/1	[42]	⊖⊕⊖⊖ VERY LOW
Neonatal intensive care unit admission	RR 1.43 (0.57 to 3.60) 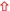	192/1	[42]	⊖⊕⊖⊖ VERY LOW
Neonatal respiratory distress	RR 6.43 (0.40 to 102.33)^c^ 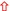	17/1	[42]	⊖⊕⊖⊖ VERY LOW
*Cranberry (Vaccinium macrocarpon) extract*				
Women without preeclampsia				
High Apgar score 5‐min Apgar score ≥ 9	RR 0.86 (0.70 to 1.06) 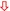	33/1	[44]	⊖⊕⊖⊖ VERY LOW
Birthweight (g)	MD‐6.00 (−340.37 to 328.37) 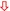	33/1	[44]	⊖⊕⊖⊖ VERY LOW
Low birthweight Birthweight < 2500 g; or otherwise as defined by authors	RR 1.36 (0.09 to 19.88) 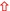	33/1	[44]	⊖⊕⊖⊖ VERY LOW
Gestational age at birth (weeks)	MD‐0.10 (−1.07 to 0.87) 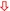	33/1	[44]	⊖⊕⊖⊖ VERY LOW
Preterm birth Birth before 37 weeks of pregnancy; or otherwise as defined by authors	RR 0.68 (0.07 to 6.76) 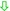	33/1	[44]	⊖⊕⊖⊖ VERY LOW
Neonatal intensive care unit admission	RR 2.71 (0.27 to 27.05) 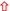	33/1	[44]	⊖⊕⊖⊖ VERY LOW

Abbreviations: MD: mean difference; RCT: randomised controlled trial; RR: risk ratio.

^a^ Legend: 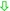 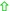 Effect in favourable direction (health benefit). 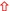 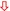 Effect in unfavourable direction (health risk/harm). Forest plots are available in File S13. Heterogeneity statistics *I* ^2^ is only provided if a meta‐analysis was conducted; otherwise, results are from single trials.

^b^ Summary effect estimate (MD) and 95% confidence interval were imputed based on *t*‐scores and *p* values of the *t*‐test.

^c^ Outcomes were measured among a subgroup of infants who were admitted to the neonatal intensive care unit.

### 3.7 Cranberry Extract ( *Vaccinium macrocarpon* )

One trial of 33 women in the United States evaluated the effect of cranberry extract in pregnant women without preeclampsia (risk factors not described), compared to placebo [46]. Evidence was very uncertain about the effects of cranberry extract on Apgar scores, birthweight, preterm birth and NICU admission (Table 4). Gastrointestinal side effects were reported, but evidence of their frequencies was very uncertain (File S12).

## 4 Discussion

### 4.1 Main Findings

This systematic review of polyphenols in pregnancy identified 14 trials of six different products, and eight additional trials currently registered, supporting our previous observation that polyphenols are an active area of research for preeclampsia [14]. However, our review findings show little compelling evidence of the efficacy and safety of polyphenols in pregnancy. All included trials had moderate to high concerns about the risk of bias, and all but two trials included fewer than 404 women. EGCG and resveratrol supplements might have beneficial effects on the management of blood pressure and hypertensive crisis episodes; however, the true effect may be substantially different from the available low‐certainty trial data. No trials have initiated treatment with polyphenols at an early stage of pregnancy (i.e., < 12 weeks); the question of the efficacy of polyphenols on the prevention of preeclampsia is thus unresolved.

### 4.2 Strengths and Limitations

The search for this review was designed to find evidence for a wide range of polyphenols and polyphenol‐containing products. For analysis, we used a statistical framework that minimises the pitfalls of under‐ or overestimation of the effect estimates when few events are present [31].

There are some limitations to this review. Most outcomes only had data from single trials or small trials, resulting in very low to low certainty of evidence. Even with this framework, many meta‐analytic effects were noninformative due to limited sample size, precluding us from accurately quantifying the treatment effect.

### 4.3 Interpretation (In Light of Other Evidence)

Preclinical literature on the included polyphenols suggests potential effects on various pathophysiological pathways of preeclampsia [17, 62]; however, the majority of this evidence comes from isolated cells in vitro, the translation of which to humans remains unknown [63, 64, 65, 66, 67]. Evidence from robust, well‐designed clinical trials is lacking for these candidates. Antihypertensive effects of Danshens and resveratrol have so far only been observed in nonpregnant populations [68, 69]. Green tea supplementation was found to reduce blood pressure in a 2020 meta‐analysis, although this decrease was unlikely to be clinically meaningful [70].

The trials included in this review used short follow‐up periods without active surveillance of adverse effects. Outside our review, safety profile evidence is scant and largely from animal studies. The safety of Danshen preparations has not been established, though it has been shown to inhibit platelet aggregation and interact with warfarin [71]. An intake level of up to 150 mg/day of resveratrol and 1000 mg/day of ECGC is generally considered safe for consumption in adults, although no specific guidance was issued for pregnant women [72, 73]. Animal studies, however, suggest that ECGC and resveratrol intake may impair early metabolism and foetal development [74, 75]. An observational study reported that consumption of *B. pinnatum* and cranberry products during pregnancy is not linked to increased risks for congenital malformations or severe drug reactions [76]. Lastly, raspberry leaf demonstrates effects on human smooth muscles (including uterine), but toxicity and obstetric complications have not been studied in clinical studies [77]. The safety of these products during pregnancy thus remains unknown.

Based on current evidence, no firm conclusions can be drawn on the efficacy and safety of polyphenol therapies for prevention or treatment of preeclampsia. The majority of trials did not report the gestational age at the initiation of intervention—a critical consideration for the management of preeclampsia. Most prevention trials initiated polyphenol intervention in late pregnancy, after which time the underlying aetiology was likely already established [78]. Currently, recommended preventive agents (e.g., aspirin) have demonstrated greater efficacy when administered before 16 gestational weeks [79]. Another challenge is the need for standardised and clearly reported dosing regimens, which may be difficult to do for some polyphenol‐containing products. Trial protocols should plan for the detection of less common adverse effects, including long‐term effects on infants.

Other polyphenol‐containing products have been investigated in clinical studies, such as curcumin ( *Curcuma longa* ) [51], silymarin ( *Silybum marianum* ) [80, 81] and *Moringa oleifera* [55, 82]. However, these studies only measured surrogate endpoints (e.g., liver transaminases, COX‐2 and anticoagulant factors), which are not this review's outcomes of interest. Future trials of these products should measure and report core maternal and neonatal outcomes for preeclampsia [28]. An ongoing trial is investigating the effect of broccoli sprout extract ( *Brassica oleracea* ) [83]. The extract contains a range of phenolic acids and flavonoids, both of which have protective effects against oxidative stress [84].

## 5 Conclusion

Polyphenols are an active area of research for novel medicines for the prevention and treatment of preeclampsia. ECGC and resveratrol supplements have been investigated for their potential effects in managing clinical signs and symptoms of preeclampsia; however, evidence of their efficacy and safety is limited and uncertain. More clinical trials, designed with appropriate timing of interventions for the prevention or treatment of preeclampsia, are needed to increase the certainty of evidence and evaluate other important maternal and neonatal outcomes.

## Author Contributions

The corresponding author attests that all listed authors meet authorship criteria and that no others meeting the criteria have been omitted. P.‐Y.N.: Funding acquisition, formal analysis, investigation, methodology and writing – original draft preparation. B.S.: Investigation, formal analysis and writing – review and editing. A.A., A.M.G., M.M., K.M.: Validation and writing – review and editing. A.M. and J.P.V.: Conceptualisation, funding acquisition, supervision and writing – review and editing.

## Ethics Statement

This is a systematic review which uses publicly available data, hence ethics approval was not required.

## Consent

The authors have nothing to report.

## Conflicts of Interest

The authors declare no conflicts of interest.

## Supporting information


**Figure S1.** Deviation from protocol.
**Figure S2.** List of eligible polyphenolic compounds and polyphenol‐rich plants.
**Figure S3.** Search strategies for all databases.
**Figure S4.** List of outcomes for meta‐analysis.
**Figure S5.** List of excluded full texts with reasons.
**Figure S6.** Ongoing trials and protocols without results.
**Figure S8.** Flowchart of study screening and selection.
**Figure S9.** Study characteristics of trials not included in analysis.
**Figure S10.** Characteristics of included trials.
**Figure S12.** Summary of results—side effects.
**Figure S13.** Forest plots.

**Figure S7.** Research integrity assessment.

**Figure S11.** Risk‐of‐bias assessment.

## Data Availability

The data that support the findings of this study are openly available in Open Science Framework at https://osf.io/c2hrp/.
