# Supplementary material for: Polyphenols for the Prevention or Management of Preeclampsia: A Systematic Review and Meta‐Analysis
Source: BJOG. 2025 Mar 3;132(7):867–79. doi: 10.1111/1471-0528.18106 (PMC12051244; doi:10.1111/1471-0528.18106)
Supplement: Supplementary file 1 — Figure S1. Deviation from protocol. Figure S2. List of eligible polyphenolic compounds and polyphenol‐rich plants. Figure S3. Search strategies for all databases. Figure S4. List of outcomes for meta‐analysis. Figure S5. List of excluded full texts with reasons. Figure S6. Ongoing trials and protocols without results. Figure S8. Flowchart of study screening and selection. Figure S9. Study characteristics of trials not included in analysis. Figure S10. Characteristics of included trials. Figure S12. Summary of results—side effects. Figure S13. Forest plots. [file BJO-132-867-s001.docx]

**CONTENT PAGE**

S1. Deviation from protocol 2

S2. List of eligible polyphenolic compounds and polyphenol-rich plants 3

S3. Search strategies for all databases 5

S4. List of outcomes for meta-analysis 39

S5. List of excluded full-texts with reasons 41

S6. Ongoing trials and protocols without result 46

S7. Research integrity assessment (separate Excel file*)

S8. Flowchart of study screening and selection 48

S9. Study characteristics of trials not included in analysis 49

S10. Characteristics of included trials 52

S11. Risk of bias assessment (separate Excel file*)

S12. Summary of results – side effects 54

S13. Forest plots 57

*Accessible via <https://osf.io/c2hrp/>

**S1. Deviation from protocol**

| **Modified plan** | **Reason for modification** |
| --- | --- |
| We searched literature for studies of polyphenolic compounds, both as food and drinks and as dietary supplements. However, for this review, we only included studies where the intervention is dietary supplement or plant extracts. | We found a large number of studies on food and drinks containing polyphenolic compounds. Therefore, these studies will be analyzed and reported in a separate review. |
| We excluded interventions in women who underwent treatment for infertility or in-vitro fertilization. | Pregnancies conceived after a long time to pregnancy or after in-vitro fertilization are associated with heightened risks of maternal and neonatal complications (2,3). We decided that the use of polyphenols in this sub-population should be investigated in a separate review to account for potential confounding effects. |
| We used an adapted version of the research integrity assessment described by Weibel and colleagues (4). Instead of excluding studies that any of the six domains, we made the final judgment based on consideration of all six domains together. | Some of the domains of the tool were considered too strict for the subject of our review, which are dietary supplements and not investigational drugs. For example, many of the studies were conducted in low-middle income countries where trial pre-registration is not common. |
| For meta-analysis, we adopted the framework for meta-analysis with few studies by Schulz and colleagues (5), which selects from a range of model choices depending on heterogeneity, number of studies, and whether the estimate was informative. | After stratifying by the polyphenolic compounds and outcomes, most meta-analyses have 3 or fewer studies. The DerSimonian-Laird random-effects method can produce biased estimates in meta-analysis of few studies, which can lead to erroneous conclusion (6,7). The new framework uses the Hartung‐Knapp‐Sidik‐Jonkman (HKSJ) method, which has been shown to outperform the DerSimonian-Laird method in this scenario (8). |
| We used R software instead of Stata for meta-analysis. | R meta package provides options to conduct the HKSJ method with and without adhoc variance, consistent with the framework described above. |
| We did not stratify our meta-analysis by intention of intervention (prevention vs treatment). | There were insufficient number of studies for stratification. Sensitivity analyses did not show substantial differences in effect estimates between these two groups of studies. Instead, we present this information in the summary of characteristics of each study. |
| We did not conduct subgroup analyses (low-middle income vs high-income countries, early vs late gestational age, low vs high risk of preeclampsia). | There were insufficient number of studies for subgroup analyses. Instead, we present this information in the summary of characteristics of each study. |
| We did not conduct sensitivity analysis by excluding studies with high risk of bias. | There were insufficient number of studies for sensitivity analyses. Instead, we present this information in the summary of characteristics of each study. |

**S2. List of eligible polyphenolic compounds and polyphenol-rich plants**

**2.1. Classes of polyphenolic compounds**

| **Flavonoids** |  | **Phenolic acids** |
| --- | --- | --- |
| ➤ Flavonols |  | ➤ Hydrobenzoic acids |
| ➤➤ Kaempferol |  | ➤➤ Ellagic acid |
| ➤➤ Myricetin |  | ➤➤ Gallic acid |
| ➤➤ Quercetin (Derivatives: rutin, rutoside, sophorin) |  | ➤➤ Protocatechuic acid |
| ➤ Flavones |  | ➤➤ Vanillic acid |
| ➤➤ Apigenin |  | ➤ Hydroxycinnamic acids |
| ➤➤ Diosmin |  | ➤➤ Caffeic acid |
| ➤➤ Luteolin (Derivatives: luteolin glucosides) |  | ➤➤ Chlorogenic acid |
| ➤➤ Nobiletin |  | ➤➤ Coumaric acid / p-coumaric acid |
| ➤➤ Vitexin |  | ➤➤ Ferulic acid |
| ➤ Isoflavones |  | ➤➤ Quinic acid |
| ➤➤ Daidzein |  | ➤➤ Sinapic acid |
| ➤➤ Genistein |  | **Stilbenes** |
| ➤➤ Glycitein |  | ➤ Pterostilbene |
| ➤➤ Biochanin A |  | ➤ Resveratrol |
| ➤➤ Formononetin |  | **Lignans** |
| ➤ Flavanones |  | ➤ Hydroxymatairesinol |
| ➤➤ Eriodictyol |  | ➤ Lariciresinol |
| ➤➤ Hesperetin |  | ➤ Matairesinol |
| ➤➤ Hesperetin |  | ➤ Pinoresinol |
| ➤➤ Naringenin |  | ➤ Secoisolariciresinol |
| ➤ Anthocyanidins |  | ➤ Sesamin |
| ➤➤ Cyanidin |  | ➤ Syrigaresinol |
| ➤➤ Delphinidin |  | **Tannins** |
| ➤➤ Malvedin |  | ➤ Ellagitannin |
| ➤➤ Pelargonidin |  | ➤ Gallotannin |
| ➤ Proanthocyanidins |  |  |
| ➤ Flavanols |  |  |
| ➤➤ Catechin (Derivatives: catechinic acid) |  |  |
| ➤➤ Epicatechin |  |  |
| ➤➤ Epigallocatechin (galleate – ECGC) |  |  |

**2.2. Plants containing polyphenolic compounds**

This is a representative, but not exhaustive, list of plants and plant-derived products that have previously been investigated in preclinical and clinical studies for their high levels of polyphenolic compounds.

| **Food** | **Main polyphenolic compound(s)** |
| --- | --- |
| Berries (blueberries, chokeberries, cranberries, gooseberries, lingonberries, whortleberries, strawberries, raspberries, blackberries, blackcurrants, mulberries, cloudberries) | Various polyphenolic compounds |
| Citrus fruits ([*Rutaceae*](https://www.google.com/search?rlz=1C1GCEA_enAU1020AU1020&sxsrf=APwXEdey7Q350NnY123k02YQFKotklfqHg:1684754828434&q=Rutaceae&stick=H4sIAAAAAAAAAONgVuLQz9U3yC4oMHrEaMwt8PLHPWEprUlrTl5jVOHiCs7IL3fNK8ksqRQS42KDsnikuLjgmngWsXIElZYkJqcmpgIAudbJwE0AAAA&sa=X&ved=2ahUKEwi2gdTo6Ij_AhUzTWwGHfwRChoQzIcDKAB6BAgkEAE) family) | Nobiletin, diosmin, hesperidins |
| Cruciferous vegetables (broccoli/kale/Brussel sprouts etc), *Brassica oleracea* | Quercetin, kaempferol and isorhamnetin |
| Danshen/Chinese sage, *Salvia miltiorrhiza* | Salvianolic acids |
| Dill, *Anethum graveolens* | Various polyphenolic compounds |
| English ivy, *Hedera helix* | Chlorogenic acid, rutin |
| Grape seed extract, *Vitis vinifera/labrusca* | Proanthocyanidins |
| Green tea, *Camellia sinensis* | Epigallocatechin gallate |
| Houpo, *Magnolia officinalis* | Honokiol |
| Huangqin/skullcap, *Scutellaria baicalensis* | Baicalein, baicalin, oroxylin A |
| Mango, *Mangifera indica* | Mangiferin |
| Milk thistle/silymarin, *Silybum marianum* | Silibinin |
| Moringa/benzolive, *Moringa oleifera* | Various polyphenolic compounds |
| Olive, *Olea europaea* | Hydroxytyrosol |
| *Piptadenia stipulacea* | Galetin-3,6-dimethyl ether, FGAL |
| Pomegranate, *Punica granatum* | Punicalagin |
| Turmeric, *curcuma longa* | Curcumin |

**S3. Search strategies for all databases**

**Embase Classic+Embase via Ovid <1947 to 11^th^ August, 2023>**

| 1 | (intake or ingest* or consum* or eat* or ate or drink* or drank).ti,ab,kf. |
| --- | --- |
| 2 | pregnancy/ or prenatal care/ or pregnancy outcome/ or perinatal care/ |
| 3 | pregnan*.ti,kf. |
| 4 | (maternal or maternity).ti,kf. |
| 5 | ((pregnan* or maternal or maternity or obstetric* or childbirth or birth) adj2 outcome*).ab. |
| 6 | (prenatal or "pre-natal" or antenatal or "ante-natal").ti,kf. |
| 7 | (peripartum or "peri-partum" or perinatal or "peri-natal").ti,kf. |
| 8 | or/2-7 |
| 9 | maternal hypertension/ |
| 10 | eclampsia and preeclampsia/ or eclampsia/ or preeclampsia/ |
| 11 | (preeclamp* or "pre-eclamp*" or eclamp*).ti,kf,ab. |
| 12 | pregnancy toxemia/ |
| 13 | (toxemi* or toxaemi*).ti,kf,ab. |
| 14 | HELLP syndrome/ |
| 15 | hellp.ti,kf,ab. |
| 16 | exp intrauterine growth retardation/ |
| 17 | ((fetal or foetal or fetus or intrauterine or "intra-uterine") adj2 ("growth restrict*" or "growth retard*" or "growth disorder*")).ti,kf,ab. |
| 18 | exp "immature and premature labor"/ |
| 19 | ((preterm or "pre-term" or premature or "pre-mature" or immature) adj2 (labor or labour or birth or infant* or neonat* or babies or baby or child*)).ti,kf,ab. |
| 20 | pregnancy disorders of endocrine origin/ or pregnancy diabetes mellitus/ or maternal diabetes mellitus/ |
| 21 | gestation* diabete*.ti,kf,ab. |
| 22 | fetus disease/ or fetal distress/ or fetus hypoxia/ |
| 23 | ((fetus or fetal or foetus or foetal) adj2 (distress or hypoxia)).ti,ab,kf. |
| 24 | or/9-23 |
| 25 | polyphenol/ or "polyphenol derivative"/ |
| 26 | polyphenol*.ti,ab,kf,du. |
| 27 | flavonoid/ or "flavonoid glycoside"/ |
| 28 | flavon*.ti,ab,kf,du. |
| 29 | Quercetin/ or "quercetin derivative"/ |
| 30 | quercetin.ti,ab,kf,du. |
| 31 | rutoside/ |
| 32 | (rutin or rutoside* or sophorin).ti,ab,kf,du. |
| 33 | kaempferol/ |
| 34 | kaempferol*.ti,ab,kf,du. |
| 35 | myricetin/ |
| 36 | myricetin*.ti,ab,kf,du. |
| 37 | flavone/ or flavone derivative/ |
| 38 | apigenin/ or apigenin derivative/ |
| 39 | apigenin*.ti,ab,kf,du. |
| 40 | luteolin/ or "luteolin 7 glucoside"/ or "luteolin 4' glucoside"/ |
| 41 | luteolin*.ti,ab,kf,du. |
| 42 | vitexin/ |
| 43 | vitexin*.ti,ab,kf,du. |
| 44 | isoflavone/ or isoflavone derivative/ |
| 45 | isoflavo*.ti,ab,kf,du. |
| 46 | daidzein/ |
| 47 | daidzein*.ti,ab,kf,du. |
| 48 | genistein/ |
| 49 | genistein*.ti,ab,kf,du. |
| 50 | glycitein/ |
| 51 | glycitein*.ti,ab,kf,du. |
| 52 | flavanol/ or flavanol derivative/ |
| 53 | flavan*.ti,ab,kf,du. |
| 54 | catechin/ |
| 55 | catechinic acid/ |
| 56 | catechol/ or catechol derivative/ |
| 57 | (catechin* or catechol or catechols).ti,ab,kf,du. |
| 58 | epicatechin/ |
| 59 | epicatechin*.ti,ab,kf,du. |
| 60 | epigallocatechin gallate/ or epigallocatechin/ |
| 61 | (epigallocatechin* or ecgc).ti,ab,kf,du. |
| 62 | flavanone derivative/ or flavanone/ |
| 63 | hesperetin/ |
| 64 | hesperetin*.ti,ab,kf,du. |
| 65 | hesperidin/ |
| 66 | hesperidin*.ti,ab,kf,du. |
| 67 | naringenin/ |
| 68 | naringenin*.ti,ab,kf,du. |
| 69 | eriodictyol/ |
| 70 | eriodictyol*.ti,ab,kf,du. |
| 71 | proanthocyanidin/ |
| 72 | proanthocyanid*.ti,ab,kf,du. |
| 73 | anthocyanidin/ |
| 74 | anthocyanins/ |
| 75 | anthocyani*.ti,ab,kf,du. |
| 76 | cyanidin chloride/ or cyanidin 3 glucoside/ |
| 77 | cyanid*.ti,ab,kf,du. |
| 78 | delphinidin/ |
| 79 | delphinid*.ti,ab,kf,du. |
| 80 | malvedin/ |
| 81 | malvedin*.ti,ab,kf,du. |
| 82 | pelargonidin/ |
| 83 | pelargonidin*.ti,ab,kf,du. |
| 84 | Phenolic acid/ |
| 85 | phenolic*.ti,ab,kf,du. |
| 86 | hydroxybenzoic acid/ or hydroxybenzoic acid derivative/ |
| 87 | (hydroxybenzo* or "p-hydroxybenzo*").ti,ab,kf,du. |
| 88 | gallic acid/ |
| 89 | (gallic* or gallate*).ti,ab,kf,du. |
| 90 | Protocatechuic acid/ or catechiuc acid/ |
| 91 | ("proto-catechuic*" or protocatechuic* or catechiuc*).ti,ab,kf,du. |
| 92 | vanillic acid/ |
| 93 | vanillic*.ti,ab,kf,du. |
| 94 | ellagic acid/ |
| 95 | ellagic*.ti,ab,kf,du. |
| 96 | hydroxycinnamic acid/ |
| 97 | hydroxycinnam*.ti,ab,kf,du. |
| 98 | coumaric acid/ |
| 99 | (coumar* or "p-coumar*").ti,ab,kf,du. |
| 100 | caffeic acid/ |
| 101 | caffeic*.ti,ab,kf,du. |
| 102 | ferulic acid/ |
| 103 | ferulic*.ti,ab,kf,du. |
| 104 | sinapic acid/ |
| 105 | sinapic*.ti,ab,kf,du. |
| 106 | chlorogenic acid/ |
| 107 | chlorogenic*.ti,ab,kf,du. |
| 108 | lignan derivative/ or lignan/ |
| 109 | lignan*.ti,ab,kf,du. |
| 110 | secoisolariciresinol/ |
| 111 | secoisolariciresinol*.ti,ab,kf,du. |
| 112 | pinoresinol dimethyl ether/ or pinoresinol/ |
| 113 | pinoresinol*.ti,ab,kf,du. |
| 114 | lariciresinol/ |
| 115 | lariciresinol*.ti,ab,kf,du. |
| 116 | syrigaresinol/ |
| 117 | syrigaresinol*.ti,ab,kf,du. |
| 118 | matairesinol/ |
| 119 | matairesinol*.ti,ab,kf,du. |
| 120 | hydroxymatairesinol/ |
| 121 | hydroxymatairesinol*.ti,ab,kf,du. |
| 122 | sesamin/ |
| 123 | sesamin*.ti,ab,kf,du. |
| 124 | stilbenes/ or "stilbene derivative"/ |
| 125 | (stilbenoid* or stilbene* or stillbenoid* or stillbene*).ti,ab,kf,du. |
| 126 | resveratrol/ |
| 127 | resveratrol.ti,ab,kf,du. |
| 128 | pterostilbene/ |
| 129 | pterostilben*.ti,ab,kf,du. |
| 130 | tannin derivative/ or tannin/ |
| 131 | (tannin* or tannic).ti,ab,kf,du. |
| 132 | gallotannin/ or ellagitannin/ |
| 133 | (gallotannin* or ellagitannin*).ti,ab,kf,du. |
| 134 | Camellia/ or tea/ or "camellia sinensis extract"/ or "green tea extract"/ |
| 135 | tea/ and 1 |
| 136 | ((tea adj5 extract) or "green tea" or Camelia or sinensis).ti,ab,kf,du. |
| 137 | Curcumin/ |
| 138 | curcumin*.ti,ab,kf,du. |
| 139 | (turmeric or curcuma or longa).ti,ab,kf,du. |
| 140 | Grape Seed Extract/ or Vitis/ or "Vitis vinifera extract"/ |
| 141 | (grape or grapes or GSE or Vitis or vinifera or labrusca or vinifera).ti,ab,kf,du. |
| 142 | Mangifera/ or "Mangifera indica extract"/ or mango/ |
| 143 | (mango or mangoes or mangifera*).ti,ab,kf,du. |
| 144 | mangiferin*.ti,ab,kf,du. |
| 145 | mangiferin/ |
| 146 | moringa/ or "Moringa oleifera extract"/ or horseradish/ |
| 147 | (Moringa or oleifera or drumstick or horseradish or "horse-radish" or "ben oil" or "benzolive").ti,ab,kf,du. |
| 148 | Pomegranate/ or "pomegranate extract"/ or punica/ or "punica granatum extract"/ |
| 149 | (pomegranate* or Punica or granatum).ti,ab,kf,du. |
| 150 | punicalagin*.ti,ab,kf,du. |
| 151 | Scutellaria/ or "Scutellaria baicalensis extract"/ |
| 152 | (Scutellaria or baicalensis or skullcap or "Huang Qin").ti,ab,kf,du. |
| 153 | baicalein/ or baicalin/ |
| 154 | baicalin*.ti,ab,kf,du. |
| 155 | oroxylin*.ti,ab,kf,du. |
| 156 | oroxylin A/ |
| 157 | Salvia/ or "Salvia miltiorrhiza extract"/ |
| 158 | ("red sage" or "Chinese sage" or "tan shen" or tanshen or "dan shen" or danshen or Salvia or miltiorrhiza).ti,ab,kf,du. |
| 159 | salvianolic acid B/ |
| 160 | Salvianolic*.ti,ab,kf,du. |
| 161 | Silymarin/ or Silybum/ |
| 162 | ("milk thistle" or Silybum or marianum or silymarin).ti,ab,kf,du. |
| 163 | silibinin/ |
| 164 | (silibinin* or silybin*).ti,ab,kf,du. |
| 165 | Brassica/ or "Brassica extract"/ |
| 166 | (Brassica* or Crucifer* or oleracea).ti,ab,kf,du. |
| 167 | (broccoli or kale or "brussel sprout*").ti,ab,kf,du. |
| 168 | Anethum/ or dill/ |
| 169 | (dill or dills or Anethum or graveolens).ti,ab,kf,du. |
| 170 | exp berry juice/ or berry/ |
| 171 | (berry or berries).ti,ab,kf,du. and 1 |
| 172 | (blueberr* or chokeberr* or cranberr* or gooseberr* or lingonberr* or whortleberr* or strawberr* or raspberr* or blackberr* or blackcurrant* or mulberr* or cloudberr*).ti,ab,kf,du. and 1 |
| 173 | piptadenia/ |
| 174 | (Piptadenia or stipulacea*).ti,ab,kf,du. |
| 175 | ("galetin-3,6-dimethyl ether" or fgal).ti,ab,kf,du. |
| 176 | Magnolia extract/ or Magnolia/ |
| 177 | magnolia.ti,ab,kf,du. |
| 178 | honokiol/ |
| 179 | (honokiol* or houpa).ti,ab,kf,du. |
| 180 | olive oil/ and 1 |
| 181 | olive oil.ti,ab,kf,du. and 1 |
| 182 | Olea/ or "olive tree"/ |
| 183 | (olea or europaea).ti,ab,kf,du. |
| 184 | hydroxytyrosol/ |
| 185 | hydroxytyrosol*.ti,ab,kf,du. |
| 186 | (citrus fruit extract/ or Citrus/ or citrus fruit/ or citrus juice/) and 1 |
| 187 | citrus.ti,ab,kf,du. and 1 |
| 188 | nobiletin/ |
| 189 | nobiletin*.ti,ab,kf,du. |
| 190 | diosmin/ |
| 191 | diosmin*.ti,ab,kf,du. |
| 192 | chocolate/ and 1 |
| 193 | chocolate.ti,ab,kf,du. and 1 |
| 194 | exp wine/ and 1 |
| 195 | wine.ti,ab,kf,du. and 1 |
| 196 | or/25-195 |
| 197 | ((mouse or rat or rats or mice or "in vitro") not (clinical or human*)).ti. |
| 198 | (editorial or "case report*" or "case stud*" or commentary).ti. |
| 199 | (animal experiment/ or animal/) not human/ |
| 200 | (editorial or Books or Chapter or erratum or letter or note).pt. |
| 201 | or/197-200 |
| 202 | ((8 or 24) and 196) not 201 |

**Ovid MEDLINE(R) and Epub Ahead of Print, In-Process, In-Data-Review & Other Non-Indexed Citations, Daily and Versions via Ovid <1946 to 11^th^ August, 2023>**

| 1 | (intake or ingest* or consum* or eat* or ate or drink* or drank).ti,ab,kf. |
| --- | --- |
| 2 | pregnancy/ or prenatal care/ or pregnancy outcome/ or perinatal care/ |
| 3 | pregnan*.ti,kf. |
| 4 | (maternal or maternity).ti,kf. |
| 5 | ((pregnan* or maternal or maternity or obstetric* or childbirth or birth) adj2 outcome*).ab. |
| 6 | (prenatal or "pre-natal" or antenatal or "ante-natal").ti,kf. |
| 7 | (peripartum or "peri-partum" or perinatal or "peri-natal").ti,kf. |
| 8 | or/2-7 |
| 9 | Pregnancy Complications, Cardiovascular/ |
| 10 | hypertension, pregnancy-induced/ or eclampsia/ or hellp syndrome/ or pre-eclampsia/ |
| 11 | (preeclamp* or "pre-eclamp*").ti,ab,kf. |
| 12 | hellp.ti,ab,kf. |
| 13 | (toxemi* or toxaemi*).ti,kf,ab. |
| 14 | Fetal Growth Retardation/ |
| 15 | ((fetal or foetal or fetus or intrauterine or "intra-uterine") adj2 ("growth restrict*" or "growth retard*" or "growth disorder*")).ti,kf,ab. |
| 16 | Premature Birth/ |
| 17 | ((preterm or "pre-term" or premature or "pre-mature" or immature) adj2 (labor or labour or birth or infant* or neonat* or babies or baby or child*)).ti,kf,ab. |
| 18 | Pregnancy in Diabetics/ |
| 19 | Diabetes, Gestational/ |
| 20 | gestation* diabete*.ti,ab,kf. |
| 21 | fetal diseases/ or fetal hypoxia/ |
| 22 | Fetal Distress/ |
| 23 | ((fetus or fetal or foetus or foetal) adj2 (distress or hypoxia)).ti,ab,kf. |
| 24 | or/9-23 |
| 25 | Polyphenols/ |
| 26 | polyphenol*.ti,ab,kf,sy,nm. |
| 27 | flavonoids/ or biflavonoids/ or flavonolignans/ or flavonols/ |
| 28 | flavon*.ti,ab,kf,sy,nm. |
| 29 | quercetin/ or rutin/ |
| 30 | quercetin.ti,ab,kf,sy,nm. |
| 31 | (rutin or rutoside or sophorin).ti,ab,kf,sy,nm. |
| 32 | Kaempferols/ |
| 33 | kaempferol*.ti,ab,kf,sy,nm. |
| 34 | myricetin/ |
| 35 | myricetin*.ti,ab,kf,sy,nm. |
| 36 | benzoflavones/ or flavones/ |
| 37 | Apigenin/ |
| 38 | apigenin*.ti,ab,kf,sy,nm. |
| 39 | Luteolin/ |
| 40 | luteolin*.ti,ab,kf,sy,nm. |
| 41 | vitexin/ |
| 42 | vitexin*.ti,ab,kf,sy,nm. |
| 43 | Isoflavones/ |
| 44 | isoflavo*.ti,ab,kf,sy,nm. |
| 45 | daidzein/ |
| 46 | daidzein*.ti,ab,kf,sy,nm. |
| 47 | genistein/ |
| 48 | genistein*.ti,ab,kf,sy,nm. |
| 49 | glycitein/ |
| 50 | glycitein*.ti,ab,kf,sy,nm. |
| 51 | flavanol/ |
| 52 | flavan*.ti,ab,kf,sy,nm. |
| 53 | Catechin/ |
| 54 | catechinic acid/ |
| 55 | Catechols/ |
| 56 | (catechin* or catechol or catechols).ti,ab,kf,sy,nm. |
| 57 | epicatechin/ |
| 58 | epicatechin*.ti,ab,kf,sy,nm. |
| 59 | epigallocatechin gallate/ or epigallocatechin/ |
| 60 | (epigallocatechin* or ecgc).ti,ab,kf,sy,nm. |
| 61 | Flavanones/ |
| 62 | Hesperidin/ |
| 63 | hesperidin*.ti,ab,kf,sy,nm. |
| 64 | hesperetin/ |
| 65 | hesperetin*.ti,ab,kf,sy,nm. |
| 66 | naringenin/ |
| 67 | naringenin*.ti,ab,kf,sy,nm. |
| 68 | eriodictyol/ |
| 69 | eriodictyol*.ti,ab,kf,sy,nm. |
| 70 | proanthocyanidin/ |
| 71 | proanthocyanid*.ti,ab,kf,sy,nm. |
| 72 | anthocyanidin/ |
| 73 | anthocyanins/ |
| 74 | anthocyani*.ti,ab,kf,sy,nm. |
| 75 | cyanidin chloride/ or cyanidin 3 glucoside/ |
| 76 | cyanid*.ti,ab,kf,sy,nm. |
| 77 | delphinidin/ |
| 78 | delphinid*.ti,ab,kf,sy,nm. |
| 79 | malvedin/ |
| 80 | malvedin*.ti,ab,kf,sy,nm. |
| 81 | pelargonidin/ |
| 82 | pelargonidin*.ti,ab,kf,sy,nm. |
| 83 | Phenolic acid/ |
| 84 | phenolic*.ti,ab,kf,sy,nm. |
| 85 | Hydroxybenzoates/ |
| 86 | (hydroxybenzo* or "p-hydroxybenzo*").ti,ab,kf,sy,nm. |
| 87 | Gallic Acid/ |
| 88 | (gallic* or gallate*).ti,ab,kf,sy,nm. |
| 89 | Protocatechuic acid/ or catechiuc acid/ |
| 90 | ("proto-catechuic*" or protocatechuic* or catechiuc*).ti,ab,kf,sy,nm. |
| 91 | Vanillic Acid/ |
| 92 | vanillic*.ti,ab,kf,sy,nm. |
| 93 | ellagic acid/ |
| 94 | ellagic*.ti,ab,kf,sy,nm. |
| 95 | hydroxycinnamic acid/ |
| 96 | hydroxycinnam*.ti,ab,kf,sy,nm. |
| 97 | coumaric acid/ |
| 98 | (coumar* or "p-coumar*").ti,ab,kf,sy,nm. |
| 99 | Caffeic Acids/ |
| 100 | caffeic*.ti,ab,kf,sy,nm. |
| 101 | ferulic acids/ |
| 102 | ferulic*.ti,ab,kf,sy,nm. |
| 103 | sinapic acids/ |
| 104 | sinapic*.ti,ab,kf,sy,nm. |
| 105 | Chlorogenic Acid/ |
| 106 | chlorogenic*.ti,ab,kf,sy,nm. |
| 107 | Lignans/ |
| 108 | lignan*.ti,ab,kf,sy,nm. |
| 109 | secoisolariciresinol/ |
| 110 | secoisolariciresinol*.ti,ab,kf,sy,nm. |
| 111 | pinoresinol/ |
| 112 | pinoresinol*.ti,ab,kf,sy,nm. |
| 113 | lariciresinol/ |
| 114 | lariciresinol*.ti,ab,kf,sy,nm. |
| 115 | syrigaresinol/ |
| 116 | syrigaresinol*.ti,ab,kf,sy,nm. |
| 117 | matairesinol/ |
| 118 | matairesinol*.ti,ab,kf,sy,nm. |
| 119 | hydroxymatairesinol/ |
| 120 | hydroxymatairesinol*.ti,ab,kf,sy,nm. |
| 121 | sesamin/ |
| 122 | sesamin*.ti,ab,kf,sy,nm. |
| 123 | Stilbenes/ |
| 124 | (stilbenoid* or stilbene* or stillbenoid* or stillbene*).ti,ab,kf,sy,nm. |
| 125 | Resveratrol/ |
| 126 | resveratrol.ti,ab,kf,sy,nm. |
| 127 | pterostilbene/ |
| 128 | pterostilben*.ti,ab,kf,sy,nm. |
| 129 | Tannins/ or Hydrolyzable Tannins/ |
| 130 | (tannin* or tannic).ti,ab,kf,sy,nm. |
| 131 | gallotannin/ or ellagitannin/ |
| 132 | (gallotannin* or ellagitannin*).ti,ab,kf,sy,nm. |
| 133 | Camellia sinensis/ or Camellia/ |
| 134 | Tea/ and 1 |
| 135 | ((tea adj5 extract) or "green tea" or Camelia or sinensis).ti,ab,kf,sy,nm. |
| 136 | Curcumin/ |
| 137 | curcumin*.ti,ab,kf,sy,nm. |
| 138 | (turmeric or curcuma or longa).ti,ab,kf,sy,nm. |
| 139 | Vitis/ |
| 140 | Grape Seed Extract/ |
| 141 | (grape or grapes or GSE or Vitis or vinifera or labrusca or vinifera).ti,ab,kf,sy,nm. |
| 142 | Mangifera/ |
| 143 | (mango or mangoes or mangifera*).ti,ab,kf,sy,nm. |
| 144 | mangiferin/ |
| 145 | mangiferin*.ti,ab,kf,sy,nm. |
| 146 | Moringa/ or Moringa oleifera/ |
| 147 | (Moringa or oleifera or drumstick or horseradish or "horse-radish" or "ben oil" or "benzolive").ti,ab,kf,sy,nm. |
| 148 | Pomegranate/ |
| 149 | (pomegranate* or Punica or granatum).ti,ab,kf,sy,nm. |
| 150 | punicalagin*.ti,ab,kf,sy,nm. |
| 151 | Scutellaria/ or Scutellaria baicalensis/ |
| 152 | (Scutellaria or baicalensis or skullcap or "Huang Qin").ti,ab,kf,sy,nm. |
| 153 | baicalein/ or baicalin/ |
| 154 | baicalin*.ti,ab,kf,sy,nm. |
| 155 | oroxylin A/ |
| 156 | oroxylin*.ti,ab,kf,sy,nm. |
| 157 | Salvia/ or Salvia miltiorrhiza/ |
| 158 | ("red sage" or "Chinese sage" or "tan shen" or tanshen or "dan shen" or danshen or Salvia or miltiorrhiza).ti,ab,kf,sy,nm. |
| 159 | salvianolic acid B/ |
| 160 | salvianolic*.ti,ab,kf,sy,nm. |
| 161 | Silymarin/ or Silybum/ |
| 162 | ("milk thistle" or Silybum or marianum or silymarin).ti,ab,kf,sy,nm. |
| 163 | silibinin/ |
| 164 | (silibinin* or silybin*).ti,ab,kf,sy,nm. |
| 165 | Brassica/ |
| 166 | Brassicaceae/ |
| 167 | (Brassica* or Crucifer* or oleracea).ti,ab,kf,sy,nm. |
| 168 | (broccoli or kale or "brussel sprout*").ti,ab,kf,sy,nm. |
| 169 | Anethum graveolens/ |
| 170 | (dill or dills or Anethum or graveolens).ti,ab,kf,sy,nm. |
| 171 | (berry or berries).ti,ab,kf,sy,nm. and 1 |
| 172 | (blueberr* or chokeberr* or cranberr* or gooseberr* or lingonberr* or whortleberr* or strawberr* or raspberr* or blackberr* or blackcurrant* or mulberr* or cloudberr*).ti,ab,kf,sy,nm. and 1 |
| 173 | piptadenia/ |
| 174 | (Piptadenia or stipulacea*).ti,ab,kf,sy,nm. |
| 175 | ("galetin-3,6-dimethyl ether" or fgal).ti,ab,kf,sy,nm. |
| 176 | Magnolia/ |
| 177 | magnolia.ti,ab,kf,sy,nm. |
| 178 | honokiol/ |
| 179 | (honokiol* or houpa).ti,ab,kf,sy,nm. |
| 180 | Olive Oil/ and 1 |
| 181 | Olea/ |
| 182 | (olea or europaea).ti,ab,kf,sy,nm. |
| 183 | olive oil.ti,ab,kf,sy,nm. and 1 |
| 184 | hydroxytyrosol/ |
| 185 | hydroxytyrosol*.ti,ab,kf,sy,nm. |
| 186 | Citrus/ and 1 |
| 187 | citrus.ti,ab,kf,sy,nm. and 1 |
| 188 | nobiletin/ |
| 189 | nobiletin*.ti,ab,kf,sy,nm. |
| 190 | diosmin/ |
| 191 | diosmin*.ti,ab,kf,sy,nm. |
| 192 | Chocolate/ and 1 |
| 193 | chocolate.ti,ab,kf,sy,nm. and 1 |
| 194 | Wine/ and 1 |
| 195 | wine.ti,ab,kf,sy,nm. and 1 |
| 196 | or/25-195 |
| 197 | ((mouse or rat or rats or mice or "in vitro") not (clinical or human*)).ti. |
| 198 | (Disease Models, Animal/ or animals/) not humans/ |
| 199 | (editorial or "case report*" or "case stud*" or commentary).ti. |
| 200 | ("Book Review" or "Case Reports" or Editorial or Essay or Lecture or "lecture note" or letter or news or "newspaper article" or "personal narrative" or "publishd erratum" or "retraction of publication" or "retracted publication" or "textbook" or "unpublished work").pt. |
| 201 | or/197-200 |
| 202 | ((8 or 24) and 196) not 201 |

**Ovid Emcare via Ovid <1995 to 2023 Week 32>**

| 1 | (intake or ingest* or consum* or eat* or ate or drink* or drank).ti,ab,kf. |
| --- | --- |
| 2 | pregnancy/ or prenatal care/ or pregnancy outcome/ or perinatal care/ |
| 3 | pregnan*.ti,kf. |
| 4 | (maternal or maternity).ti,kf. |
| 5 | ((pregnan* or maternal or maternity or obstetric* or childbirth or birth) adj2 outcome*).ab. |
| 6 | (prenatal or "pre-natal" or antenatal or "ante-natal").ti,kf. |
| 7 | (peripartum or "peri-partum" or perinatal or "peri-natal").ti,kf. |
| 8 | or/2-7 |
| 9 | maternal hypertension/ |
| 10 | eclampsia and preeclampsia/ or eclampsia/ or preeclampsia/ |
| 11 | (preeclamp* or "pre-eclamp*" or eclamp*).ti,kf,ab. |
| 12 | pregnancy toxemia/ |
| 13 | (toxemi* or toxaemi*).ti,kf,ab. |
| 14 | HELLP syndrome/ |
| 15 | hellp.ti,kf,ab. |
| 16 | exp intrauterine growth retardation/ |
| 17 | ((fetal or foetal or fetus or intrauterine or "intra-uterine") adj2 ("growth restrict*" or "growth retard*" or "growth disorder*")).ti,kf,ab. |
| 18 | exp "immature and premature labor"/ |
| 19 | ((preterm or "pre-term" or premature or "pre-mature" or immature) adj2 (labor or labour or birth or infant* or neonat* or babies or baby or child*)).ti,kf,ab. |
| 20 | pregnancy disorders of endocrine origin/ or pregnancy diabetes mellitus/ or maternal diabetes mellitus/ |
| 21 | gestation* diabete*.ti,kf,ab. |
| 22 | fetus disease/ or fetal distress/ or fetus hypoxia/ |
| 23 | ((fetus or fetal or foetus or foetal) adj2 (distress or hypoxia)).ti,ab,kf. |
| 24 | or/9-23 |
| 25 | polyphenol/ or "polyphenol derivative"/ |
| 26 | polyphenol*.ti,ab,kf,tn. |
| 27 | flavonoid/ or "flavonoid glycoside"/ |
| 28 | flavon*.ti,ab,kf,tn. |
| 29 | Quercetin/ or "quercetin derivative"/ |
| 30 | quercetin.ti,ab,kf,tn. |
| 31 | rutoside/ |
| 32 | (rutin or rutoside* or sophorin).ti,ab,kf,tn. |
| 33 | kaempferol/ |
| 34 | kaempferol*.ti,ab,kf,tn. |
| 35 | myricetin/ |
| 36 | myricetin*.ti,ab,kf,tn. |
| 37 | flavone/ or flavone derivative/ |
| 38 | apigenin/ or apigenin derivative/ |
| 39 | apigenin*.ti,ab,kf,tn. |
| 40 | luteolin/ or "luteolin 7 glucoside"/ or "luteolin 4' glucoside"/ |
| 41 | luteolin*.ti,ab,kf,tn. |
| 42 | vitexin/ |
| 43 | vitexin*.ti,ab,kf,tn. |
| 44 | isoflavone/ or isoflavone derivative/ |
| 45 | isoflavo*.ti,ab,kf,tn. |
| 46 | daidzein/ |
| 47 | daidzein*.ti,ab,kf,tn. |
| 48 | genistein/ |
| 49 | genistein*.ti,ab,kf,tn. |
| 50 | glycitein/ |
| 51 | glycitein*.ti,ab,kf,tn. |
| 52 | flavanol/ or flavanol derivative/ |
| 53 | flavan*.ti,ab,kf,tn. |
| 54 | catechin/ |
| 55 | catechinic acid/ |
| 56 | catechol/ or catechol derivative/ |
| 57 | (catechin* or catechol or catechols).ti,ab,kf,tn. |
| 58 | epicatechin/ |
| 59 | epicatechin*.ti,ab,kf,tn. |
| 60 | epigallocatechin gallate/ or epigallocatechin/ |
| 61 | (epigallocatechin* or ecgc).ti,ab,kf,tn. |
| 62 | flavanone derivative/ or flavanone/ |
| 63 | hesperetin/ |
| 64 | hesperetin*.ti,ab,kf,tn. |
| 65 | hesperidin/ |
| 66 | hesperidin*.ti,ab,kf,tn. |
| 67 | naringenin/ |
| 68 | naringenin*.ti,ab,kf,tn. |
| 69 | eriodictyol/ |
| 70 | eriodictyol*.ti,ab,kf,tn. |
| 71 | proanthocyanidin/ |
| 72 | proanthocyanid*.ti,ab,kf,tn. |
| 73 | anthocyanidin/ |
| 74 | anthocyanins/ |
| 75 | anthocyani*.ti,ab,kf,tn. |
| 76 | cyanidin chloride/ or cyanidin 3 glucoside/ |
| 77 | cyanid*.ti,ab,kf,tn. |
| 78 | delphinidin/ |
| 79 | delphinid*.ti,ab,kf,tn. |
| 80 | malvedin/ |
| 81 | malvedin*.ti,ab,kf,tn. |
| 82 | pelargonidin/ |
| 83 | pelargonidin*.ti,ab,kf,tn. |
| 84 | Phenolic acid/ |
| 85 | phenolic*.ti,ab,kf,tn. |
| 86 | hydroxybenzoic acid/ or hydroxybenzoic acid derivative/ |
| 87 | (hydroxybenzo* or "p-hydroxybenzo*").ti,ab,kf,tn. |
| 88 | gallic acid/ |
| 89 | (gallic* or gallate*).ti,ab,kf,tn. |
| 90 | Protocatechuic acid/ or catechiuc acid/ |
| 91 | ("proto-catechuic*" or protocatechuic* or catechiuc*).ti,ab,kf,tn. |
| 92 | vanillic acid/ |
| 93 | vanillic*.ti,ab,kf,tn. |
| 94 | ellagic acid/ |
| 95 | ellagic*.ti,ab,kf,tn. |
| 96 | hydroxycinnamic acid/ |
| 97 | hydroxycinnam*.ti,ab,kf,tn. |
| 98 | coumaric acid/ |
| 99 | (coumar* or "p-coumar*").ti,ab,kf,tn. |
| 100 | caffeic acid/ |
| 101 | caffeic*.ti,ab,kf,tn. |
| 102 | ferulic acid/ |
| 103 | ferulic*.ti,ab,kf,tn. |
| 104 | sinapic acid/ |
| 105 | sinapic*.ti,ab,kf,tn. |
| 106 | chlorogenic acid/ |
| 107 | chlorogenic*.ti,ab,kf,tn. |
| 108 | lignan derivative/ or lignan/ |
| 109 | lignan*.ti,ab,kf,tn. |
| 110 | secoisolariciresinol/ |
| 111 | secoisolariciresinol*.ti,ab,kf,tn. |
| 112 | pinoresinol dimethyl ether/ or pinoresinol/ |
| 113 | pinoresinol*.ti,ab,kf,tn. |
| 114 | lariciresinol/ |
| 115 | lariciresinol*.ti,ab,kf,tn. |
| 116 | syrigaresinol/ |
| 117 | syrigaresinol*.ti,ab,kf,tn. |
| 118 | matairesinol/ |
| 119 | matairesinol*.ti,ab,kf,tn. |
| 120 | hydroxymatairesinol/ |
| 121 | hydroxymatairesinol*.ti,ab,kf,tn. |
| 122 | sesamin/ |
| 123 | sesamin*.ti,ab,kf,tn. |
| 124 | stilbenes/ or "stilbene derivative"/ |
| 125 | (stilbenoid* or stilbene* or stillbenoid* or stillbene*).ti,ab,kf,tn. |
| 126 | resveratrol/ |
| 127 | resveratrol.ti,ab,kf,tn. |
| 128 | pterostilbene/ |
| 129 | pterostilben*.ti,ab,kf,tn. |
| 130 | tannin derivative/ or tannin/ |
| 131 | (tannin* or tannic).ti,ab,kf,tn. |
| 132 | gallotannin/ or ellagitannin/ |
| 133 | (gallotannin* or ellagitannin*).ti,ab,kf,tn. |
| 134 | Camellia/ or tea/ or "camellia sinensis extract"/ or "green tea extract"/ |
| 135 | tea/ and 1 |
| 136 | ((tea adj5 extract) or "green tea" or Camelia or sinensis).ti,ab,kf,tn. |
| 137 | Curcumin/ |
| 138 | curcumin*.ti,ab,kf,tn. |
| 139 | (turmeric or curcuma or longa).ti,ab,kf,tn. |
| 140 | Grape Seed Extract/ or Vitis/ or "Vitis vinifera extract"/ |
| 141 | (grape or grapes or GSE or Vitis or vinifera or labrusca or vinifera).ti,ab,kf,tn. |
| 142 | Mangifera/ or "Mangifera indica extract"/ or mango/ |
| 143 | (mango or mangoes or mangifera*).ti,ab,kf,tn. |
| 144 | mangiferin*.ti,ab,kf,tn. |
| 145 | mangiferin/ |
| 146 | moringa/ or "Moringa oleifera extract"/ or horseradish/ |
| 147 | (Moringa or oleifera or drumstick or horseradish or "horse-radish" or "ben oil" or "benzolive").ti,ab,kf,tn. |
| 148 | Pomegranate/ or "pomegranate extract"/ or punica/ or "punica granatum extract"/ |
| 149 | (pomegranate* or Punica or granatum).ti,ab,kf,tn. |
| 150 | punicalagin*.ti,ab,kf,tn. |
| 151 | Scutellaria/ or "Scutellaria baicalensis extract"/ |
| 152 | (Scutellaria or baicalensis or skullcap or "Huang Qin").ti,ab,kf,tn. |
| 153 | baicalein/ or baicalin/ |
| 154 | baicalin*.ti,ab,kf,tn. |
| 155 | oroxylin*.ti,ab,kf,tn. |
| 156 | oroxylin A/ |
| 157 | Salvia/ or "Salvia miltiorrhiza extract"/ |
| 158 | ("red sage" or "Chinese sage" or "tan shen" or tanshen or "dan shen" or danshen or Salvia or miltiorrhiza).ti,ab,kf,tn. |
| 159 | salvianolic acid B/ |
| 160 | Salvianolic*.ti,ab,kf,tn. |
| 161 | Silymarin/ or Silybum/ |
| 162 | ("milk thistle" or Silybum or marianum or silymarin).ti,ab,kf,tn. |
| 163 | silibinin/ |
| 164 | (silibinin* or silybin*).ti,ab,kf,tn. |
| 165 | Brassica/ or "Brassica extract"/ |
| 166 | (Brassica* or Crucifer* or oleracea).ti,ab,kf,tn. |
| 167 | (broccoli or kale or "brussel sprout*").ti,ab,kf,tn. |
| 168 | Anethum/ or dill/ |
| 169 | (dill or dills or Anethum or graveolens).ti,ab,kf,tn. |
| 170 | exp berry juice/ or berry/ |
| 171 | (berry or berries).ti,ab,kf,tn. and 1 |
| 172 | (blueberr* or chokeberr* or cranberr* or gooseberr* or lingonberr* or whortleberr* or strawberr* or raspberr* or blackberr* or blackcurrant* or mulberr* or cloudberr*).ti,ab,kf,tn. and 1 |
| 173 | piptadenia/ |
| 174 | (Piptadenia or stipulacea*).ti,ab,kf,tn. |
| 175 | ("galetin-3,6-dimethyl ether" or fgal).ti,ab,kf,tn. |
| 176 | Magnolia extract/ or Magnolia/ |
| 177 | magnolia.ti,ab,kf,tn. |
| 178 | honokiol/ |
| 179 | (honokiol* or houpa).ti,ab,kf,tn. |
| 180 | olive oil/ and 1 |
| 181 | olive oil.ti,ab,kf,tn. and 1 |
| 182 | Olea/ or "olive tree"/ |
| 183 | (olea or europaea).ti,ab,kf,tn. |
| 184 | hydroxytyrosol/ |
| 185 | hydroxytyrosol*.ti,ab,kf,tn. |
| 186 | (citrus fruit extract/ or Citrus/ or citrus fruit/ or citrus juice/) and 1 |
| 187 | citrus.ti,ab,kf,tn. and 1 |
| 188 | nobiletin/ |
| 189 | nobiletin*.ti,ab,kf,tn. |
| 190 | diosmin/ |
| 191 | diosmin*.ti,ab,kf,tn. |
| 192 | chocolate/ and 1 |
| 193 | chocolate.ti,ab,kf,tn. and 1 |
| 194 | exp wine/ and 1 |
| 195 | wine.ti,ab,kf,tn. and 1 |
| 196 | or/25-195 |
| 197 | ((mouse or rat or rats or mice or "in vitro") not (clinical or human*)).ti. |
| 198 | (editorial or "case report*" or "case stud*" or commentary).ti. |
| 199 | (animal experiment/ or animal/) not human/ |
| 200 | (editorial or Books or Chapter or erratum or letter or note).pt. |
| 201 | or/197-200 |
| 202 | ((8 or 24) and 196) not 201 |

**CINAHL Complete via EBSCOHost <Sunday, 11^th^ August, 2023>**

| 1 | TI (intake or ingest* or consum* or eat* or ate or drink* or drank) OR AB (intake or ingest* or consum* or eat* or ate or drink* or drank) OR SU (intake or ingest* or consum* or eat* or ate or drink* or drank) |
| --- | --- |
| 2 | MH "Pregnancy" |
| 3 | MH "Prenatal Care" OR MH "Prenatal Care (Iowa NIC)" |
| 4 | MH "Perinatal Care" |
| 5 | MH "Pregnancy Outcomes" |
| 6 | TI (pregnan*) OR SU (pregnan*) |
| 7 | TI (maternal or maternity) OR SU (maternal or maternity) |
| 8 | AB ((pregnan* or maternal or maternity or obstetric* or childbirth or birth) N2 outcome*) |
| 9 | TI (prenatal or "pre-natal" or antenatal or "ante-natal") OR SU (prenatal or "pre-natal" or antenatal or "ante-natal") |
| 10 | TI (peripartum or "peri-partum" or perinatal or "peri-natal") OR SU (peripartum or "peri-partum" or perinatal or "peri-natal") |
| 11 | S2 OR S3 OR S4 OR S5 OR S6 OR S7 OR S8 OR S9 OR S10 |
| 12 | MH "Pregnancy Complications, Cardiovascular" |
| 13 | MH "Pregnancy-Induced Hypertension" |
| 14 | MH "Eclampsia" |
| 15 | MH "Pre-Eclampsia" |
| 16 | TI (preeclamp* or "pre-eclamp*" or eclamp*) OR AB (preeclamp* or "pre-eclamp*" or eclamp*) OR SU (preeclamp* or "pre-eclamp*" or eclamp*) |
| 17 | TI (toxemi* or toxaemi*) OR AB (toxemi* or toxaemi*) OR SU (toxemi* or toxaemi*) |
| 18 | MH "HELLP Syndrome" |
| 19 | TI (hellp) OR AB (hellp) OR SU (hellp) |
| 20 | MH "Fetal Growth Retardation" |
| 21 | TI ((fetal or foetal or fetus or intrauterine or "intra-uterine") N2 ("growth restrict*" or "growth retard*" or "growth disorder*")) OR AB ((fetal or foetal or fetus or intrauterine or "intra-uterine") N2 ("growth restrict*" or "growth retard*" or "growth disorder*")) OR SU ((fetal or foetal or fetus or intrauterine or "intra-uterine") N2 ("growth restrict*" or "growth retard*" or "growth disorder*")) |
| 22 | MH "Labor, Premature" |
| 23 | TI ((preterm or "pre-term" or premature or "pre-mature" or immature) N2 (labor or labour or birth or infant* or neonat* or babies or baby or child*)) OR AB ((preterm or "pre-term" or premature or "pre-mature" or immature) N2 (labor or labour or birth or infant* or neonat* or babies or baby or child*)) OR SU ((preterm or "pre-term" or premature or "pre-mature" or immature) N2 (labor or labour or birth or infant* or neonat* or babies or baby or child*)) |
| 24 | MH "Diabetes Mellitus, Gestational" |
| 25 | MH "Pregnancy in Diabetes" |
| 26 | TI ("gestation* diabete*") OR AB ("gestation* diabete*") OR SU ("gestation* diabete*") |
| 27 | MH "Fetal Distress" OR MH "Fetal Diseases" |
| 28 | TI ((fetus or fetal or foetus or foetal) N2 (distress or hypoxia)) OR AB ((fetus or fetal or foetus or foetal) N2 (distress or hypoxia)) OR SU ((fetus or fetal or foetus or foetal) N2 (distress or hypoxia)) |
| 29 | S12 OR S13 OR S14 OR S15 OR S16 OR S17 OR S18 OR S19 OR S20 OR S21 OR S22 OR S23 OR S24 OR S25 OR S26 OR S27 OR S28 |
| 30 | MH "Polyphenols" |
| 31 | TI (polyphenol*) OR AB (polyphenol*) OR SU (polyphenol*) |
| 32 | MH "Flavonoids" |
| 33 | MH "Bioflavonoids" |
| 34 | MH "Flavonols" |
| 35 | TI (flavon*) OR AB (flavon*) OR SU (flavon*) |
| 36 | MH "Quercetin" |
| 37 | TI (quercetin) OR AB (quercetin) OR SU (quercetin) |
| 38 | MH "Rutin" |
| 39 | TI (rutin or rutoside* or sophorin) OR AB (rutin or rutoside* or sophorin) OR SU (rutin or rutoside* or sophorin) |
| 40 | TI (kaempferol*) OR AB (kaempferol*) OR SU (kaempferol*) |
| 41 | TI (myricetin*) OR AB (myricetin*) OR SU (myricetin*) |
| 42 | MH "Flavones" |
| 43 | TI (apigenin*) OR AB (apigenin*) OR SU (apigenin*) |
| 44 | TI (luteolin*) OR AB (luteolin*) OR SU (luteolin*) |
| 45 | TI (vitexin*) OR AB (vitexin*) OR SU (vitexin*) |
| 46 | MH "Isoflavones" |
| 47 | TI (isoflavo*) OR AB (isoflavo*) OR SU (isoflavo*) |
| 48 | TI (daidzein*) OR AB (daidzein*) OR SU (daidzein*) |
| 49 | MH "Genistein" |
| 50 | TI (genistein*) OR AB (genistein*) OR SU (genistein*) |
| 51 | TI (glycitein*) OR AB (glycitein*) OR SU (glycitein*) |
| 52 | TI (flavan*) OR AB (flavan*) OR SU (flavan*) |
| 53 | TI (catechin* or catechol or catechols) OR AB (catechin* or catechol or catechols) OR SU (catechin* or catechol or catechols) |
| 54 | TI (epicatechin*) OR AB (epicatechin*) OR SU (epicatechin*) |
| 55 | TI (epigallocatechin* or ecgc) OR AB (epigallocatechin* or ecgc) OR SU (epigallocatechin* or ecgc) |
| 56 | MH "Flavanones" |
| 57 | TI (hesperetin*) OR AB (hesperetin*) OR SU (hesperetin*) |
| 58 | TI (hesperidin*) OR AB (hesperidin*) OR SU (hesperidin*) |
| 59 | TI (naringenin*) OR AB (naringenin*) OR SU (naringenin*) |
| 60 | TI (eriodictyol*) OR AB (eriodictyol*) OR SU (eriodictyol*) |
| 61 | TI (proanthocyanid*) OR AB (proanthocyanid*) OR SU (proanthocyanid*) |
| 62 | TI (anthocyani*) OR AB (anthocyani*) OR SU (anthocyani*) |
| 63 | TI (cyanid*) OR AB (cyanid*) OR SU (cyanid*) |
| 64 | TI (delphinid*) OR AB (delphinid*) OR SU (delphinid*) |
| 65 | TI (malvedin*) OR AB (malvedin*) OR SU (malvedin*) |
| 66 | TI (pelargonidin*) OR AB (pelargonidin*) OR SU (pelargonidin*) |
| 67 | TI (phenolic*) OR AB (phenolic*) OR SU (phenolic*) |
| 68 | TI (hydroxybenzo* or "p-hydroxybenzo*") OR AB (hydroxybenzo* or "p-hydroxybenzo*") OR SU (hydroxybenzo* or "p-hydroxybenzo*") |
| 69 | TI (gallic* or gallate*) OR AB (gallic* or gallate*) OR SU (gallic* or gallate*) |
| 70 | TI ("proto-catechuic*" or protocatechuic* or catechiuc*) OR AB ("proto-catechuic*" or protocatechuic* or catechiuc*) OR SU ("proto-catechuic*" or protocatechuic* or catechiuc*) |
| 71 | TI (vanillic*) OR AB (vanillic*) OR SU (vanillic*) |
| 72 | TI (ellagic*) OR AB (ellagic*) OR SU (ellagic*) |
| 73 | TI (hydroxycinnam*) OR AB (hydroxycinnam*) OR SU (hydroxycinnam*) |
| 74 | TI (coumar* or "p-coumar*") OR AB (coumar* or "p-coumar*") OR SU (coumar* or "p-coumar*") |
| 75 | TI (caffeic*) OR AB (caffeic*) OR SU (caffeic*) |
| 76 | TI (ferulic*) OR AB (ferulic*) OR SU (ferulic*) |
| 77 | TI (sinapic*) OR AB (sinapic*) OR SU (sinapic*) |
| 78 | TI (chlorogenic*) OR AB (chlorogenic*) OR SU (chlorogenic*) |
| 79 | TI (lignan*) OR AB (lignan*) OR SU (lignan*) |
| 80 | MH "Lignans" |
| 81 | TI (secoisolariciresinol*) OR AB (secoisolariciresinol*) OR SU (secoisolariciresinol*) |
| 82 | TI (pinoresinol*) OR AB (pinoresinol*) OR SU (pinoresinol*) |
| 83 | TI (lariciresinol*) OR AB (lariciresinol*) OR SU (lariciresinol*) |
| 84 | TI (syrigaresinol*) OR AB (syrigaresinol*) OR SU (syrigaresinol*) |
| 85 | TI (matairesinol*) OR AB (matairesinol*) OR SU (matairesinol*) |
| 86 | TI (hydroxymatairesinol*) OR AB (hydroxymatairesinol*) OR SU (hydroxymatairesinol*) |
| 87 | TI (sesamin*) OR AB (sesamin*) OR SU (sesamin*) |
| 88 | MH "Stilbenes" |
| 89 | TI (stilbenoid* or stilbene* or stillbenoid* or stillbene*) OR AB (stilbenoid* or stilbene* or stillbenoid* or stillbene*) OR SU (stilbenoid* or stilbene* or stillbenoid* or stillbene*) |
| 90 | MH "Resveratrol" |
| 91 | TI (resveratrol) OR AB (resveratrol) OR SU (resveratrol) |
| 92 | TI (pterostilben*) OR AB (pterostilben*) OR SU (pterostilben*) |
| 93 | MH "Tannins" |
| 94 | TI (tannin* or tannic) OR AB (tannin* or tannic) OR SU (tannin* or tannic) |
| 95 | TI (gallotannin* or ellagitannin*) OR AB (gallotannin* or ellagitannin*) OR SU (gallotannin* or ellagitannin*) |
| 96 | (MH "Green Tea") |
| 97 | TI ((tea N5 extract) or "green tea" or Camelia or sinensis) OR AB ((tea N5 extract) or "green tea" or Camelia or sinensis) OR SU ((tea N5 extract) or "green tea" or Camelia or sinensis) |
| 98 | MH "Curcumin" |
| 99 | TI (curcumin*) OR AB (curcumin*) OR SU (curcumin*) |
| 100 | TI (turmeric or curcuma or longa) OR AB (turmeric or curcuma or longa) OR SU (turmeric or curcuma or longa) |
| 101 | MH "Grape Seed Extract" |
| 102 | MH "Grape" |
| 103 | TI (grape or grapes or GSE or Vitis or vinifera or labrusca or vinifera) OR AB (grape or grapes or GSE or Vitis or vinifera or labrusca or vinifera) OR SU (grape or grapes or GSE or Vitis or vinifera or labrusca or vinifera) |
| 104 | TI (mango or mangoes or mangifera*) OR AB (mango or mangoes or mangifera*) OR SU (mango or mangoes or mangifera*) |
| 105 | TI (mangiferin*) OR AB (mangiferin*) OR SU (mangiferin*) |
| 106 | MH "Moringa oleifera" |
| 107 | TI (Moringa or oleifera or drumstick or horseradish or "horse-radish" or "ben oil" or "benzolive") OR AB (Moringa or oleifera or drumstick or horseradish or "horse-radish" or "ben oil" or "benzolive") OR SU (Moringa or oleifera or drumstick or horseradish or "horse-radish" or "ben oil" or "benzolive") |
| 108 | MH "Pomegranate" |
| 109 | TI (pomegranate* or Punica or granatum) OR AB (pomegranate* or Punica or granatum) OR SU (pomegranate* or Punica or granatum) |
| 110 | TI (punicalagin*) OR AB (punicalagin*) OR SU (punicalagin*) |
| 111 | MH "Scutellaria baicalensis" |
| 112 | TI (Scutellaria or baicalensis or skullcap or "Huang Qin") OR AB (Scutellaria or baicalensis or skullcap or "Huang Qin") OR SU (Scutellaria or baicalensis or skullcap or "Huang Qin") |
| 113 | TI (baicalin*) OR AB (baicalin*) OR SU (baicalin*) |
| 114 | TI (oroxylin*) OR AB (oroxylin*) OR SU (oroxylin*) |
| 115 | MH "Salvia miltiorrhiza" |
| 116 | TI ("red sage" or "Chinese sage" or "tan shen" or tanshen or "dan shen" or danshen or Salvia or miltiorrhiza) OR AB ("red sage" or "Chinese sage" or "tan shen" or tanshen or "dan shen" or danshen or Salvia or miltiorrhiza) OR SU ("red sage" or "Chinese sage" or "tan shen" or tanshen or "dan shen" or danshen or Salvia or miltiorrhiza) |
| 117 | TI (Salvianolic*) OR AB (Salvianolic*) OR SU (Salvianolic*) |
| 118 | MH "Silymarin" |
| 119 | TI ("milk thistle" or Silybum or marianum or silymarin) OR AB ("milk thistle" or Silybum or marianum or silymarin) OR SU ("milk thistle" or Silybum or marianum or silymarin) |
| 120 | MH "Milk thistle" |
| 121 | TI (silibinin* or silybin*) OR AB (silibinin* or silybin*) OR SU (silibinin* or silybin*) |
| 122 | MH "Cruciferous Vegetables" |
| 123 | TI (Brassica* or Crucifer* or oleracea) OR AB (Brassica* or Crucifer* or oleracea) OR SU (Brassica* or Crucifer* or oleracea) |
| 124 | TI (broccoli or kale or "brussel sprout*") OR AB (broccoli or kale or "brussel sprout*") OR SU (broccoli or kale or "brussel sprout*") |
| 125 | TI (dill or dills or Anethum or graveolens) OR AB (dill or dills or Anethum or graveolens) OR SU (dill or dills or Anethum or graveolens) |
| 126 | MH "Berries+" |
| 127 | (TI (berry or berries) OR AB (berry or berries) OR SU (berry or berries)) |
| 128 | (TI (blueberr* or chokeberr* or cranberr* or gooseberr* or lingonberr* or whortleberr* or strawberr* or raspberr* or blackberr* or blackcurrant* or mulberr* or cloudberr*) OR AB (blueberr* or chokeberr* or cranberr* or gooseberr* or lingonberr* or whortleberr* or strawberr* or raspberr* or blackberr* or blackcurrant* or mulberr* or cloudberr*) OR SU (blueberr* or chokeberr* or cranberr* or gooseberr* or lingonberr* or whortleberr* or strawberr* or raspberr* or blackberr* or blackcurrant* or mulberr* or cloudberr*)) and S1 |
| 129 | TI (Piptadenia or stipulacea*) OR AB (Piptadenia or stipulacea*) OR SU (Piptadenia or stipulacea*) |
| 130 | TI ("galetin-3,6-dimethyl ether" or fgal) OR AB ("galetin-3,6-dimethyl ether" or fgal) OR SU ("galetin-3,6-dimethyl ether" or fgal) |
| 131 | TI (magnolia) OR AB (magnolia) OR SU (magnolia) |
| 132 | TI (honokiol* or houpa) OR AB (honokiol* or houpa) OR SU (honokiol* or houpa) |
| 133 | MH "Olive oil" |
| 134 | (TI ("olive oil") OR AB ("olive oil") OR SU ("olive oil")) |
| 135 | MH "Olive" |
| 136 | TI (olea or europaea) OR AB (olea or europaea) OR SU (olea or europaea) |
| 137 | TI (hydroxytyrosol*) OR AB (hydroxytyrosol*) OR SU (hydroxytyrosol*) |
| 138 | MH "Citrus+" |
| 139 | (TI (citrus) OR AB (citrus) OR SU (citrus)) |
| 140 | TI (nobiletin*) OR AB (nobiletin*) OR SU (nobiletin*) |
| 141 | TI (diosmin*) OR AB (diosmin*) OR SU (diosmin*) |
| 142 | MH "Cacao" |
| 143 | (TI (chocolate) OR AB (chocolate) OR SU (chocolate)) |
| 144 | MH "Wine" |
| 145 | (TI (wine) OR AB (wine) OR SU (wine)) |
| 146 | S30 OR S31 OR S32 OR S33 OR S34 OR S35 OR S36 OR S37 OR S38 OR S39 OR S40 OR S41 OR S42 OR S43 OR S44 OR S45 OR S46 OR S47 OR S48 OR S49 OR S50 OR S51 OR S52 OR S53 OR S54 OR S55 OR S56 OR S57 OR S58 OR S59 OR S60 OR S61 OR S62 OR S63 OR S64 OR S65 OR S66 OR S67 OR S68 OR S69 OR S70 OR S71 OR S72 OR S73 OR S74 OR S75 OR S76 OR S77 OR S78 OR S79 OR S80 OR S81 OR S82 OR S83 OR S84 OR S85 OR S86 OR S87 OR S88 OR S89 OR S90 OR S91 OR S92 OR S93 OR S94 OR S95 OR S96 OR S97 OR S98 OR S99 OR S100 OR S101 OR S102 OR S103 OR S104 OR S105 OR S106 OR S107 OR S108 OR S109 OR S110 OR S111 OR S112 OR S113 OR S114 OR S115 OR S116 OR S117 OR S118 OR S119 OR S120 OR S121 OR S122 OR S123 OR S124 OR S125 OR S126 OR S127 OR S128 OR S129 OR S130 OR S131 OR S132 OR S133 OR S134 OR S135 OR S136 OR S137 OR S138 OR S139 OR S140 OR S141 OR S142 OR S143 OR S144 OR S145 |
| 147 | TI ((mouse or rat or rats or mice or "in vitro") not (clinical or human*)) |
| 148 | TI (editorial or "case report*" or "case stud*" or commentary) |
| 149 | (MH "Animal studies" or MH "Animals") NOT (MH "Clinical research" or MH "Human") |
| 150 | PT "ANECDOTE" OR PT "BOOK" OR PT "BOOK CHAPTER" OR PT "CASE STUDY" OR PT "COMMENTARY" OR PT "EDITORIAL" OR PT "LETTER" OR PT "QUESTIONS & ANSWERS" OR PT "RESPONSE" OR PT "STANDARDS" OR PT "TEACHING MATERIALS" OR PT "WEBSITE" |
| 151 | S147 OR S148 OR S149 OR S150 |
| 152 | (S11 or S29) and S146 not S151 |

**Maternity & Infant Care Database (MIDIRS) <1971 to 11^th^ August, 2023>**

| 1 | polyphenol*.ti,ab,hw,ss,sx. |
| --- | --- |
| 2 | flavon*.ti,ab,hw,ss,sx. |
| 3 | quercetin.ti,ab,hw,ss,sx. |
| 4 | (rutin or rutoside* or sophorin).ti,ab,hw,ss,sx. |
| 5 | kaempferol*.ti,ab,hw,ss,sx. |
| 6 | myricetin*.ti,ab,hw,ss,sx. |
| 7 | apigenin*.ti,ab,hw,ss,sx. |
| 8 | luteolin*.ti,ab,hw,ss,sx. |
| 9 | vitexin*.ti,ab,hw,ss,sx. |
| 10 | isoflavo*.ti,ab,hw,ss,sx. |
| 11 | daidzein*.ti,ab,hw,ss,sx. |
| 12 | genistein*.ti,ab,hw,ss,sx. |
| 13 | glycitein*.ti,ab,hw,ss,sx. |
| 14 | flavan*.ti,ab,hw,ss,sx. |
| 15 | (catechin* or catechol or catechols).ti,ab,hw,ss,sx. |
| 16 | epicatechin*.ti,ab,hw,ss,sx. |
| 17 | (epigallocatechin* or ecgc).ti,ab,hw,ss,sx. |
| 18 | hesperetin*.ti,ab,hw,ss,sx. |
| 19 | hesperidin*.ti,ab,hw,ss,sx. |
| 20 | naringenin*.ti,ab,hw,ss,sx. |
| 21 | eriodictyol*.ti,ab,hw,ss,sx. |
| 22 | proanthocyanid*.ti,ab,hw,ss,sx. |
| 23 | anthocyani*.ti,ab,hw,ss,sx. |
| 24 | cyanid*.ti,ab,hw,ss,sx. |
| 25 | delphinid*.ti,ab,hw,ss,sx. |
| 26 | malvedin*.ti,ab,hw,ss,sx. |
| 27 | pelargonidin*.ti,ab,hw,ss,sx. |
| 28 | phenolic*.ti,ab,hw,ss,sx. |
| 29 | (hydroxybenzo* or "p-hydroxybenzo*").ti,ab,hw,ss,sx. |
| 30 | (gallic* or gallate*).ti,ab,hw,ss,sx. |
| 31 | ("proto-catechuic*" or protocatechuic* or catechiuc*).ti,ab,hw,ss,sx. |
| 32 | vanillic*.ti,ab,hw,ss,sx. |
| 33 | ellagic*.ti,ab,hw,ss,sx. |
| 34 | hydroxycinnam*.ti,ab,hw,ss,sx. |
| 35 | (coumar* or "p-coumar*").ti,ab,hw,ss,sx. |
| 36 | caffeic*.ti,ab,hw,ss,sx. |
| 37 | ferulic*.ti,ab,hw,ss,sx. |
| 38 | sinapic*.ti,ab,hw,ss,sx. |
| 39 | chlorogenic*.ti,ab,hw,ss,sx. |
| 40 | lignan*.ti,ab,hw,ss,sx. |
| 41 | secoisolariciresinol*.ti,ab,hw,ss,sx. |
| 42 | pinoresinol*.ti,ab,hw,ss,sx. |
| 43 | lariciresinol*.ti,ab,hw,ss,sx. |
| 44 | syrigaresinol*.ti,ab,hw,ss,sx. |
| 45 | matairesinol*.ti,ab,hw,ss,sx. |
| 46 | hydroxymatairesinol*.ti,ab,hw,ss,sx. |
| 47 | sesamin*.ti,ab,hw,ss,sx. |
| 48 | (stilbenoid* or stilbene* or stillbenoid* or stillbene*).ti,ab,hw,ss,sx. |
| 49 | resveratrol.ti,ab,hw,ss,sx. |
| 50 | pterostilben*.ti,ab,hw,ss,sx. |
| 51 | (tannin* or tannic).ti,ab,hw,ss,sx. |
| 52 | (gallotannin* or ellagitannin*).ti,ab,hw,ss,sx. |
| 53 | ((tea adj5 extract) or "green tea" or Camelia or sinensis).ti,ab,hw,ss,sx. |
| 54 | curcumin*.ti,ab,hw,ss,sx. |
| 55 | (grape or grapes or GSE or Vitis or vinifera or labrusca or vinifera).ti,ab,hw,ss,sx. |
| 56 | (mango or mangoes or mangifera*).ti,ab,hw,ss,sx. |
| 57 | mangiferin*.ti,ab,hw,ss,sx. |
| 58 | (Moringa or oleifera or drumstick or horseradish or "horse-radish" or "ben oil" or "benzolive").ti,ab,hw,ss,sx. |
| 59 | (pomegranate* or Punica or granatum).ti,ab,hw,ss,sx. |
| 60 | punicalagin*.ti,ab,hw,ss,sx. |
| 61 | (Scutellaria or baicalensis or skullcap or "Huang Qin").ti,ab,hw,ss,sx. |
| 62 | baicalin*.ti,ab,hw,ss,sx. |
| 63 | oroxylin*.ti,ab,hw,ss,sx. |
| 64 | ("red sage" or "Chinese sage" or "tan shen" or tanshen or "dan shen" or danshen or Salvia or miltiorrhiza).ti,ab,hw,ss,sx. |
| 65 | Salvianolic*.ti,ab,hw,ss,sx. |
| 66 | ("milk thistle" or Silybum or marianum or silymarin).ti,ab,hw,ss,sx. |
| 67 | (silibinin* or silybin*).ti,ab,hw,ss,sx. |
| 68 | (Brassica* or Crucifer* or oleracea).ti,ab,hw,ss,sx. |
| 69 | (broccoli or kale or "brussel sprout*").ti,ab,hw,ss,sx. |
| 70 | (dill or dills or Anethum or graveolens).ti,ab,hw,ss,sx. |
| 71 | (berry or berries).ti,ab,hw,ss,sx. |
| 72 | (blueberr* or chokeberr* or cranberr* or gooseberr* or lingonberr* or whortleberr* or strawberr* or raspberr* or blackberr* or blackcurrant* or mulberr* or cloudberr*).ti,ab,hw,ss,sx. |
| 73 | (Piptadenia or stipulacea*).ti,ab,hw,ss,sx. |
| 74 | ("galetin-3,6-dimethyl ether" or fgal).ti,ab,hw,ss,sx. |
| 75 | magnolia.ti,ab,hw,ss,sx. |
| 76 | (honokiol* or houpa).ti,ab,hw,ss,sx. |
| 77 | olive oil.ti,ab,hw,ss,sx. |
| 78 | (olea or europaea).ti,ab,hw,ss,sx. |
| 79 | hydroxytyrosol*.ti,ab,hw,ss,sx. |
| 80 | citrus.ti,ab,hw,ss,sx. |
| 81 | nobiletin*.ti,ab,hw,ss,sx. |
| 82 | diosmin*.ti,ab,hw,ss,sx. |
| 83 | chocolate.ti,ab,hw,ss,sx. |
| 84 | wine.ti,ab,hw,ss,sx. |
| 85 | or/1-84 |

**Global Index Medicus <last date of search: 11^th^ August, 2023>**

| Concept | Search strategy |
| --- | --- |
| Pregnancy | mh:("pregnancy") OR mh:("prenatal care") OR mh:("pregnancy outcome") OR mh:("perinatal care") OR ti:(pregnan*) OR ti:(maternal or maternity) OR ab:((pregnan* or maternal or maternity or obstetric* or childbirth or birth) and outcome*) OR ti:(prenatal or "pre-natal" or antenatal or "ante-natal") OR ti:(peripartum or "peri-partum" or perinatal or "peri-natal") |
| Preeclampsia | mh:("Pregnancy Complications, Cardiovascular") OR mh:("hypertension, pregnancy-induced") OR mh:(eclampsia) OR mh:("hellp syndrome") OR mh:("pre-eclampsia") OR ti:(preeclamp* or "pre-eclamp*") OR ab:(preeclamp* or "pre-eclamp*") OR ti:(hellp) OR ab:(hellp) OR ti:(toxemi* or toxaemi*) OR ab:(toxemi* or toxaemi*) OR mh:("Fetal Growth Retardation") OR ti:((fetal or foetal or fetus or intrauterine or "intra-uterine") and ("growth restrict*" or "growth retard*" or "growth disorder*")) OR ab:((fetal or foetal or fetus or intrauterine or "intra-uterine") and ("growth restrict*" or "growth retard*" or "growth disorder*") |
| Polyphenols | mh:(Polyphenols) OR ti:(polyphenol*) OR ab:(polyphenol*) OR mh:(flavonoids) OR mh:(biflavonoids) OR mh:(flavonolignans) OR mh:(flavonols) OR ti:(flavon*) OR ab:(flavon*) OR mh:(quercetin) OR mh:(rutin) OR ti:(quercetin) OR ab:(quercetin) OR ti:(rutin or rutoside or sophorin) OR ab:(rutin or rutoside or sophorin) OR mh:(Kaempferols) OR ti:(kaempferol*) OR ab:(kaempferol*) OR mh:(myricetin) OR ti:(myricetin*) OR ab:(myricetin*) OR mh:(benzoflavones) OR mh:(flavones) OR mh:(Apigenin) OR ti:(apigenin*) OR ab:(apigenin*) OR mh:(Luteolin) OR ti:(luteolin*) OR ab:(luteolin*) OR mh:(vitexin) OR ti:(vitexin*) OR ab:(vitexin*) OR mh:(Isoflavones) OR ti:(isoflavo*) OR ab:(isoflavo*) OR mh:(daidzein) OR ti:(daidzein*) OR ab:(daidzein*) OR mh:(genistein) OR ti:(genistein*) OR ab:(genistein*) mh:(glycitein) OR ti:(glycitein*) OR ab:(glycitein*) OR mh:(flavanol) OR ti:(flavan*) OR ab:(flavan*) OR mh:(Catechin) OR mh:(catechinic acid) OR mh:(Catechols) OR ti:(catech*) OR ab:(catech*) OR mh:(epicatechin) OR ti:(epicatechin*) OR ab:(epicatechin*) OR mh:("epigallocatechin gallate") OR mh:(epigallocatechin) OR ti:(epigallocatechin* or ecgc) OR ab:(epigallocatechin* or ecgc) OR mh:(Flavanones) OR mh:(Hesperidin) OR ti:(hesperidin*) OR ab:(hesperidin*) OR mh:(hesperetin) OR ti:(hesperetin*) OR ab:(hesperetin*) OR mh:(naringenin) OR ti:(naringenin*) OR ab:(naringenin*) OR mh:(eriodictyol) OR ti:(eriodictyol*) OR ab:(eriodictyol*) OR mh:(proanthocyanidin) OR ti:(proanthocyanid*) OR ab:(proanthocyanid*) OR mh:(anthocyanidin) OR mh:(anthocyanins) OR ti:(anthocyani*) OR ab:(anthocyani*) OR mh:("cyanidin chloride") OR mh:("cyanidin 3 glucoside") OR ti:(cyanid*) OR ab:(cyanid*) OR mh:(delphinidin) OR ti:(delphinid*) OR ab:(delphinid*) OR mh:(malvedin) OR ti:(malvedin*) OR ab:(malvedin*) OR mh:(pelargonidin) OR ti:(pelargonidin*) OR ab:(pelargonidin*) OR mh:("Phenolic acid") OR ti:(phenolic*) OR ab:(phenolic*) OR mh:(Hydroxybenzoates) OR ti:(hydroxybenzo* or "p-hydroxybenzo*") OR ab:(hydroxybenzo* or "p-hydroxybenzo*") OR mh:("Gallic Acid") OR ti:(gallic* or gallate*) OR ab:(gallic* or gallate*) OR mh:("Protocatechuic acid") OR mh:("catechiuc acid") OR ti:("proto-catechuic*" or protocatechuic* or catechiuc*) OR ab:("proto-catechuic*" or protocatechuic* or catechiuc*) OR mh:("Vanillic Acid") OR ti:(vanillic*) OR ab:(vanillic*) OR mh:("ellagic acid") OR ti:(ellagic*) OR ab:(ellagic*) OR mh:("hydroxycinnamic acid") OR ti:(hydroxycinnam*) OR ab:(hydroxycinnam*) OR mh:("coumaric acid") OR ti:(coumar* or "p-coumar*") OR ab:(coumar* or "p-coumar*") OR mh:("Caffeic Acids") OR ti:(caffeic*) OR ab:(caffeic*) OR mh:("ferulic acids") OR ti:(ferulic*) OR ab:(ferulic*) OR mh:("sinapic acids") OR ti:(sinapic*) OR ab:(sinapic*) OR mh:("Chlorogenic Acid") OR ti:(chlorogenic*) OR ab:(chlorogenic*) OR mh:(Lignans) OR ti:(lignan*) OR ab:(lignan*) OR mh:(secoisolariciresinol) OR ti:(secoisolariciresinol*) OR ab:(secoisolariciresinol*) OR mh:(pinoresinol) OR ti:(pinoresinol*) OR ab:(pinoresinol*) OR mh:(lariciresinol) OR ti:(lariciresinol*) OR ab:(lariciresinol*) OR mh:(syrigaresinol) OR ti:(syrigaresinol*) OR ab:(syrigaresinol*) OR mh:(matairesinol) OR ti:(matairesinol*) OR ab:(matairesinol*) OR mh:(hydroxymatairesinol) OR ti:(hydroxymatairesinol*) OR ab:(hydroxymatairesinol*) OR mh:(sesamin) OR ti:(sesamin*) OR ab:(sesamin*) OR mh:(Stilbenes) OR ti:(stilbenoid* or stilbene* or stillbenoid* or stillbene*) OR ab:(stilbenoid* or stilbene* or stillbenoid* or stillbene*) OR mh:(Resveratrol) OR ti:(resveratrol) OR ab:(resveratrol) OR mh:(pterostilbene) OR ti:(pterostilben*) OR ab:(pterostilben*) OR mh:(Tannins) OR mh:("Hydrolyzable Tannins") OR ti:(tannin* or tannic) OR ab:(tannin* or tannic) OR mh:(gallotannin) OR mh:(ellagitannin) OR ti:(gallotannin* or ellagitannin*) OR ab:(gallotannin* or ellagitannin*) OR mh:("Camellia sinensis") OR mh:(Camellia) OR mh:(Tea) OR ti:("tea extract" or "green tea" or Camelia or sinensis) OR ab:("tea extract" or "green tea" or Camelia or sinensis) OR mh:(Curcumin) OR ti:(curcumin*) OR ab:(curcumin*) OR mh:(Vitis) OR mh:("Grape Seed Extract") OR ti:(grape or grapes or GSE or Vitis or vinifera or labrusca or vinifera) OR ab:(grape or grapes or GSE or Vitis or vinifera or labrusca or vinifera) OR mh:(Mangifera) OR ti:(mango or mangoes or mangifera*) OR ab:(mango or mangoes or mangifera*) OR mh:(mangiferin) OR ti:(mangiferin*) OR ab:(mangiferin*) OR mh:(Moringa) OR mh:("Moringa oleifera") OR ti:(Moringa or oleifera or drumstick or horseradish or "horse-radish" or "ben oil" or "benzolive") OR ab:(Moringa or oleifera or drumstick or horseradish or "horse-radish" or "ben oil" or "benzolive") OR mh:(Pomegranate) OR ti:(pomegranate* or Punica or granatum) OR ab:(pomegranate* or Punica or granatum) OR ti:(punicalagin*) OR ab:(punicalagin*) OR mh:(Scutellaria ) OR mh:("Scutellaria baicalensis") OR ti:(Scutellaria or baicalensis or skullcap or "Huang Qin") OR ab:(Scutellaria or baicalensis or skullcap or "Huang Qin") OR mh:(baicalein) OR mh:(baicalin) OR ti:(baicalin*) OR ab:(baicalin*) OR mh:("oroxylin A") OR ti:(oroxylin*) OR ab:(oroxylin*) OR mh:(Salvia) OR mh:("Salvia miltiorrhiza") OR ti:("red sage" or "Chinese sage" or "tan shen" or tanshen or "dan shen" or danshen or Salvia or miltiorrhiza) OR ab:("red sage" or "Chinese sage" or "tan shen" or tanshen or "dan shen" or danshen or Salvia or miltiorrhiza) OR mh:("salvianolic acid B") OR ti:(salvianolic*) OR ab:(salvianolic*) OR mh:(Silymarin) OR mh:(Silybum) OR ti:("milk thistle" or Silybum or marianum or silymarin) OR ab:("milk thistle" or Silybum or marianum or silymarin) OR mh:(silibinin) OR ti:(silibinin* or silybin*) OR ab:(silibinin* or silybin*) OR mh:(Brassica) OR mh:(Brassicaceae) OR ti:(Brassica* or Crucifer* or oleracea) OR ab:(Brassica* or Crucifer* or oleracea) OR ti:(broccoli or kale or "brussel sprout*") OR ab:(broccoli or kale or "brussel sprout*") OR mh:("Anethum graveolens") OR ti:(dill or dills or Anethum or graveolens) OR ab:(dill or dills or Anethum or graveolens) OR ti:(berry or berries) OR ab:(berry or berries) OR ti:(blueberr* or chokeberr* or cranberr* or gooseberr* or lingonberr* or whortleberr* or strawberr* or raspberr* or blackberr*or blackcurrant* or mulberr* or cloudberr*) OR ab:(blueberr* or chokeberr* or cranberr* or gooseberr* or lingonberr* or whortleberr* or strawberr* or raspberr* or blackberr* or blackcurrant* or mulberr* or cloudberr*) OR mh:(piptadenia) OR ti:(Piptadenia or stipulacea*) OR ab:(Piptadenia or stipulacea*) OR ti:("galetin-3,6-dimethyl ether" or fgal) OR ab:("galetin-3,6-dimethyl ether" or fgal) OR mh:(Magnolia) OR ti:(magnolia) OR ab:(magnolia) OR mh:(honokiol) OR ti:(honokiol* or houpa) OR ab:(honokiol* or houpa) OR mh:("Olive Oil") OR mh:(Olea) OR ti:(olea or europaea) OR ab:(olea or europaea) OR ti:("olive oil") OR ab:("olive oil") OR mh:(hydroxytyrosol) OR ti:(hydroxytyrosol*) OR ab:(hydroxytyrosol*) OR mh:(Citrus) OR ti:(citrus) OR ab:(citrus) OR mh:(nobiletin) OR ti:(nobiletin*) OR ab:(nobiletin*) OR mh:(diosmin) OR ti:(diosmin*) OR ab:(diosmin*) OR mh:(Chocolate) OR ti:(chocolate) OR ab:(chocolate) OR mh:(Wine) OR ti:(wine) OR ab:(wine) |

Note: The search components were combined into the full search strategy as (pregnancy OR preeclampsia) AND (polyphenols). Due to the interface’s restriction, the full search strategy was broken down into small searches.

**AMED (Allied and Complementary Medicine) <1985 to 11^th^ August, 2023>**

| 1 | (intake or ingest* or consum* or eat* or ate or drink* or drank).ti,ab,hw,sh. |
| --- | --- |
| 2 | pregnancy/ or labor obstetric/ |
| 3 | obstetrical care/ or prenatal care/ |
| 4 | pregnancy complications/ or obstetric labor complications/ |
| 5 | pregnan*.ti,hw,sh. |
| 6 | (maternal or maternity).ti,hw,sh. |
| 7 | ((pregnan* or maternal or maternity or obstetric* or childbirth or birth) adj2 outcome*).ab. |
| 8 | (prenatal or "pre-natal" or antenatal or "ante-natal").ti,hw,sh. |
| 9 | (peripartum or "peri-partum" or perinatal or "peri-natal").ti,hw,sh. |
| 10 | or/2-9 |
| 11 | pre eclampsia/ or pregnancy toxemias/ |
| 12 | (preeclamp* or "pre-eclamp*" or eclamp*).ti,hw,sh,ab. |
| 13 | (toxemi* or toxaemi*).ti,hw,sh,ab. |
| 14 | hellp.ti,hw,sh,ab. |
| 15 | ((fetal or foetal or fetus or intrauterine or "intra-uterine") adj2 ("growth restrict*" or "growth retard*" or "growth disorder*")).ti,hw,sh,ab. |
| 16 | Infant premature/ |
| 17 | ((preterm or "pre-term" or premature or "pre-mature" or immature) adj2 (labor or labour or birth or infant* or neonat* or babies or baby or child*)).ti,hw,sh,ab. |
| 18 | gestation* diabete*.ti,hw,sh,ab. |
| 19 | fetal death/ or fetal disease/ |
| 20 | ((fetus or fetal or foetus or foetal) adj2 (distress or hypoxia)).ti,ab,hw,sh. |
| 21 | or/11-20 |
| 22 | exp Plant extracts/ |
| 23 | exp Plants medicinal/ |
| 24 | exp Drugs chinese herbal/ |
| 25 | exp Traditional medicine chinese/ |
| 26 | exp Herbal drugs/ |
| 27 | exp Phytotherapy/ |
| 28 | exp Dietary supplements/ |
| 29 | phenols/ |
| 30 | polyphenol*.ti,hw,sh. |
| 31 | phenolic*.ti,hw,sh. |
| 32 | Bioflavonoids/ |
| 33 | flavonols/ |
| 34 | Quercetin/ |
| 35 | quercetin.ti,ab,hw,sh. |
| 36 | (rutin or rutoside* or sophorin).ti,ab,hw,sh. |
| 37 | kaempferol*.ti,ab,hw,sh. |
| 38 | myricetin*.ti,ab,hw,sh. |
| 39 | flavones/ |
| 40 | apigenin*.ti,ab,hw,sh. |
| 41 | luteolin*.ti,ab,hw,sh. |
| 42 | vitexin*.ti,ab,hw,sh. |
| 43 | Isoflavones/ |
| 44 | isoflavo*.ti,ab,hw,sh. |
| 45 | daidzein*.ti,ab,hw,sh. |
| 46 | genistein*.ti,ab,hw,sh. |
| 47 | glycitein*.ti,ab,hw,sh. |
| 48 | flavan*.ti,ab,hw,sh. |
| 49 | (catechin* or catechol or catechols).ti,ab,hw,sh. |
| 50 | epicatechin*.ti,ab,hw,sh. |
| 51 | (epigallocatechin* or ecgc).ti,ab,hw,sh. |
| 52 | hesperetin*.ti,ab,hw,sh. |
| 53 | hesperidin*.ti,ab,hw,sh. |
| 54 | naringenin*.ti,ab,hw,sh. |
| 55 | eriodictyol*.ti,ab,hw,sh. |
| 56 | proanthocyanid*.ti,ab,hw,sh. |
| 57 | anthocyani*.ti,ab,hw,sh. |
| 58 | cyanid*.ti,ab,hw,sh. |
| 59 | delphinid*.ti,ab,hw,sh. |
| 60 | malvedin*.ti,ab,hw,sh. |
| 61 | pelargonidin*.ti,ab,hw,sh. |
| 62 | phenolic*.ti,ab,hw,sh. |
| 63 | (hydroxybenzo* or "p-hydroxybenzo*").ti,ab,hw,sh. |
| 64 | (gallic* or gallate*).ti,ab,hw,sh. |
| 65 | ("proto-catechuic*" or protocatechuic* or catechiuc*).ti,ab,hw,sh. |
| 66 | vanillic*.ti,ab,hw,sh. |
| 67 | ellagic*.ti,ab,hw,sh. |
| 68 | hydroxycinnam*.ti,ab,hw,sh. |
| 69 | (coumar* or "p-coumar*").ti,ab,hw,sh. |
| 70 | caffeic*.ti,ab,hw,sh. |
| 71 | ferulic*.ti,ab,hw,sh. |
| 72 | sinapic*.ti,ab,hw,sh. |
| 73 | chlorogenic*.ti,ab,hw,sh. |
| 74 | lignans/ |
| 75 | lignan*.ti,ab,hw,sh. |
| 76 | secoisolariciresinol*.ti,ab,hw,sh. |
| 77 | pinoresinol*.ti,ab,hw,sh. |
| 78 | lariciresinol*.ti,ab,hw,sh. |
| 79 | syrigaresinol*.ti,ab,hw,sh. |
| 80 | matairesinol*.ti,ab,hw,sh. |
| 81 | hydroxymatairesinol*.ti,ab,hw,sh. |
| 82 | sesamin*.ti,ab,hw,sh. |
| 83 | Stilbenes/ |
| 84 | (stilbenoid* or stilbene* or stillbenoid* or stillbene*).ti,ab,hw,sh. |
| 85 | resveratrol.ti,ab,hw,sh. |
| 86 | pterostilben*.ti,ab,hw,sh. |
| 87 | tannins/ |
| 88 | (tannin* or tannic).ti,ab,hw,sh. |
| 89 | (gallotannin* or ellagitannin*).ti,ab,hw,sh. |
| 90 | camellia/ |
| 91 | tea/ |
| 92 | ((tea adj5 extract) or "green tea" or Camelia or sinensis).ti,ab,hw,sh. |
| 93 | Curcuma/ |
| 94 | curcumin*.ti,ab,hw,sh. |
| 95 | (grape or grapes or GSE or Vitis or vinifera or labrusca or vinifera).ti,ab,hw,sh. |
| 96 | Mangifera/ or "Mangifera indica extract"/ or mango/ |
| 97 | (mango or mangoes or mangifera*).ti,ab,hw,sh. |
| 98 | mangiferin*.ti,ab,hw,sh. |
| 99 | moringa/ or "Moringa oleifera extract"/ or horseradish/ |
| 100 | (Moringa or oleifera or drumstick or horseradish or "horse-radish" or "ben oil" or "benzolive").ti,ab,hw,sh. |
| 101 | Pomegranate/ or "pomegranate extract"/ or punica/ or "punica granatum extract"/ |
| 102 | (pomegranate* or Punica or granatum).ti,ab,hw,sh. |
| 103 | punicalagin*.ti,ab,hw,sh. |
| 104 | Scutellaria/ or "Scutellaria baicalensis extract"/ |
| 105 | (Scutellaria or baicalensis or skullcap or "Huang Qin").ti,ab,hw,sh. |
| 106 | baicalein/ or baicalin/ |
| 107 | baicalin*.ti,ab,hw,sh. |
| 108 | oroxylin*.ti,ab,hw,sh. |
| 109 | oroxylin A/ |
| 110 | Salvia/ or "Salvia miltiorrhiza extract"/ |
| 111 | ("red sage" or "Chinese sage" or "tan shen" or tanshen or "dan shen" or danshen or Salvia or miltiorrhiza).ti,ab,hw,sh. |
| 112 | salvianolic acid B/ |
| 113 | Salvianolic*.ti,ab,hw,sh. |
| 114 | Silymarin/ or Silybum/ |
| 115 | ("milk thistle" or Silybum or marianum or silymarin).ti,ab,hw,sh. |
| 116 | silibinin/ |
| 117 | (silibinin* or silybin*).ti,ab,hw,sh. |
| 118 | Brassica/ or "Brassica extract"/ |
| 119 | (Brassica* or Crucifer* or oleracea).ti,ab,hw,sh. |
| 120 | (broccoli or kale or "brussel sprout*").ti,ab,hw,sh. |
| 121 | Anethum/ or dill/ |
| 122 | (dill or dills or Anethum or graveolens).ti,ab,hw,sh. |
| 123 | (berry or berries).ti,ab,hw,sh. |
| 124 | (blueberr* or chokeberr* or cranberr* or gooseberr* or lingonberr* or whortleberr* or strawberr* or raspberr* or blackberr* or blackcurrant* or mulberr* or cloudberr*).ti,ab,hw,sh. |
| 125 | piptadenia/ |
| 126 | (Piptadenia or stipulacea*).ti,ab,hw,sh. |
| 127 | ("galetin-3,6-dimethyl ether" or fgal).ti,ab,hw,sh. |
| 128 | Magnolia extract/ or Magnolia/ |
| 129 | magnolia.ti,ab,hw,sh. |
| 130 | honokiol/ |
| 131 | (honokiol* or houpa).ti,ab,hw,sh. |
| 132 | olive oil/ |
| 133 | olive oil.ti,ab,hw,sh. |
| 134 | Olea/ or "olive tree"/ |
| 135 | (olea or europaea).ti,ab,hw,sh. |
| 136 | hydroxytyrosol/ |
| 137 | hydroxytyrosol*.ti,ab,hw,sh. |
| 138 | citrus aurantiifolia/ or citrus paradisi/ or citrus sinensis/ |
| 139 | citrus.ti,ab,hw,sh. |
| 140 | nobiletin/ |
| 141 | nobiletin*.ti,ab,hw,sh. |
| 142 | diosmin/ |
| 143 | diosmin*.ti,ab,hw,sh. |
| 144 | chocolate.ti,ab,hw,sh. |
| 145 | wine.ti,ab,hw,sh. |
| 146 | or/22-145 |
| 147 | ((mouse or rat or rats or mice or "in vitro") not (clinical or human*)).ti. |
| 148 | (editorial or "case report*" or "case stud*" or commentary).ti. |
| 149 | or/147-148 |
| 150 | ((10 or 21) and 146) not 149 |

**Cochrane CDSR and CENTRAL (last date of search: 11^th^ August, 2023)**

| 1 | TI (intake or ingest* or consum* or eat* or ate or drink* or drank) OR AB (intake or ingest* or consum* or eat* or ate or drink* or drank) OR SU (intake or ingest* or consum* or eat* or ate or drink* or drank) |
| --- | --- |
| 2 | MH "Pregnancy" |
| 3 | MH "Prenatal Care" OR MH "Prenatal Care (Iowa NIC)" |
| 4 | MH "Perinatal Care" |
| 5 | MH "Pregnancy Outcomes" |
| 6 | TI (pregnan*) OR SU (pregnan*) |
| 7 | TI (maternal or maternity) OR SU (maternal or maternity) |
| 8 | AB ((pregnan* or maternal or maternity or obstetric* or childbirth or birth) N2 outcome*) |
| 9 | TI (prenatal or "pre-natal" or antenatal or "ante-natal") OR SU (prenatal or "pre-natal" or antenatal or "ante-natal") |
| 10 | TI (peripartum or "peri-partum" or perinatal or "peri-natal") OR SU (peripartum or "peri-partum" or perinatal or "peri-natal") |
| 11 | S2 OR S3 OR S4 OR S5 OR S6 OR S7 OR S8 OR S9 OR S10 |
| 12 | MH "Pregnancy Complications, Cardiovascular" |
| 13 | MH "Pregnancy-Induced Hypertension" |
| 14 | MH "Eclampsia" |
| 15 | MH "Pre-Eclampsia" |
| 16 | TI (preeclamp* or "pre-eclamp*" or eclamp*) OR AB (preeclamp* or "pre-eclamp*" or eclamp*) OR SU (preeclamp* or "pre-eclamp*" or eclamp*) |
| 17 | TI (toxemi* or toxaemi*) OR AB (toxemi* or toxaemi*) OR SU (toxemi* or toxaemi*) |
| 18 | MH "HELLP Syndrome" |
| 19 | TI (hellp) OR AB (hellp) OR SU (hellp) |
| 20 | MH "Fetal Growth Retardation" |
| 21 | TI ((fetal or foetal or fetus or intrauterine or "intra-uterine") N2 ("growth restrict*" or "growth retard*" or "growth disorder*")) OR AB ((fetal or foetal or fetus or intrauterine or "intra-uterine") N2 ("growth restrict*" or "growth retard*" or "growth disorder*")) OR SU ((fetal or foetal or fetus or intrauterine or "intra-uterine") N2 ("growth restrict*" or "growth retard*" or "growth disorder*")) |
| 22 | MH "Labor, Premature" |
| 23 | TI ((preterm or "pre-term" or premature or "pre-mature" or immature) N2 (labor or labour or birth or infant* or neonat* or babies or baby or child*)) OR AB ((preterm or "pre-term" or premature or "pre-mature" or immature) N2 (labor or labour or birth or infant* or neonat* or babies or baby or child*)) OR SU ((preterm or "pre-term" or premature or "pre-mature" or immature) N2 (labor or labour or birth or infant* or neonat* or babies or baby or child*)) |
| 24 | MH "Diabetes Mellitus, Gestational" |
| 25 | MH "Pregnancy in Diabetes" |
| 26 | TI ("gestation* diabete*") OR AB ("gestation* diabete*") OR SU ("gestation* diabete*") |
| 27 | MH "Fetal Distress" OR MH "Fetal Diseases" |
| 28 | TI ((fetus or fetal or foetus or foetal) N2 (distress or hypoxia)) OR AB ((fetus or fetal or foetus or foetal) N2 (distress or hypoxia)) OR SU ((fetus or fetal or foetus or foetal) N2 (distress or hypoxia)) |
| 29 | S12 OR S13 OR S14 OR S15 OR S16 OR S17 OR S18 OR S19 OR S20 OR S21 OR S22 OR S23 OR S24 OR S25 OR S26 OR S27 OR S28 |
| 30 | MH "Polyphenols" |
| 31 | TI (polyphenol*) OR AB (polyphenol*) OR SU (polyphenol*) |
| 32 | MH "Flavonoids" |
| 33 | MH "Bioflavonoids" |
| 34 | MH "Flavonols" |
| 35 | TI (flavon*) OR AB (flavon*) OR SU (flavon*) |
| 36 | MH "Quercetin" |
| 37 | TI (quercetin) OR AB (quercetin) OR SU (quercetin) |
| 38 | MH "Rutin" |
| 39 | TI (rutin or rutoside* or sophorin) OR AB (rutin or rutoside* or sophorin) OR SU (rutin or rutoside* or sophorin) |
| 40 | TI (kaempferol*) OR AB (kaempferol*) OR SU (kaempferol*) |
| 41 | TI (myricetin*) OR AB (myricetin*) OR SU (myricetin*) |
| 42 | MH "Flavones" |
| 43 | TI (apigenin*) OR AB (apigenin*) OR SU (apigenin*) |
| 44 | TI (luteolin*) OR AB (luteolin*) OR SU (luteolin*) |
| 45 | TI (vitexin*) OR AB (vitexin*) OR SU (vitexin*) |
| 46 | MH "Isoflavones" |
| 47 | TI (isoflavo*) OR AB (isoflavo*) OR SU (isoflavo*) |
| 48 | TI (daidzein*) OR AB (daidzein*) OR SU (daidzein*) |
| 49 | MH "Genistein" |
| 50 | TI (genistein*) OR AB (genistein*) OR SU (genistein*) |
| 51 | TI (glycitein*) OR AB (glycitein*) OR SU (glycitein*) |
| 52 | TI (flavan*) OR AB (flavan*) OR SU (flavan*) |
| 53 | TI (catechin* or catechol or catechols) OR AB (catechin* or catechol or catechols) OR SU (catechin* or catechol or catechols) |
| 54 | TI (epicatechin*) OR AB (epicatechin*) OR SU (epicatechin*) |
| 55 | TI (epigallocatechin* or ecgc) OR AB (epigallocatechin* or ecgc) OR SU (epigallocatechin* or ecgc) |
| 56 | MH "Flavanones" |
| 57 | TI (hesperetin*) OR AB (hesperetin*) OR SU (hesperetin*) |
| 58 | TI (hesperidin*) OR AB (hesperidin*) OR SU (hesperidin*) |
| 59 | TI (naringenin*) OR AB (naringenin*) OR SU (naringenin*) |
| 60 | TI (eriodictyol*) OR AB (eriodictyol*) OR SU (eriodictyol*) |
| 61 | TI (proanthocyanid*) OR AB (proanthocyanid*) OR SU (proanthocyanid*) |
| 62 | TI (anthocyani*) OR AB (anthocyani*) OR SU (anthocyani*) |
| 63 | TI (cyanid*) OR AB (cyanid*) OR SU (cyanid*) |
| 64 | TI (delphinid*) OR AB (delphinid*) OR SU (delphinid*) |
| 65 | TI (malvedin*) OR AB (malvedin*) OR SU (malvedin*) |
| 66 | TI (pelargonidin*) OR AB (pelargonidin*) OR SU (pelargonidin*) |
| 67 | TI (phenolic*) OR AB (phenolic*) OR SU (phenolic*) |
| 68 | TI (hydroxybenzo* or "p-hydroxybenzo*") OR AB (hydroxybenzo* or "p-hydroxybenzo*") OR SU (hydroxybenzo* or "p-hydroxybenzo*") |
| 69 | TI (gallic* or gallate*) OR AB (gallic* or gallate*) OR SU (gallic* or gallate*) |
| 70 | TI ("proto-catechuic*" or protocatechuic* or catechiuc*) OR AB ("proto-catechuic*" or protocatechuic* or catechiuc*) OR SU ("proto-catechuic*" or protocatechuic* or catechiuc*) |
| 71 | TI (vanillic*) OR AB (vanillic*) OR SU (vanillic*) |
| 72 | TI (ellagic*) OR AB (ellagic*) OR SU (ellagic*) |
| 73 | TI (hydroxycinnam*) OR AB (hydroxycinnam*) OR SU (hydroxycinnam*) |
| 74 | TI (coumar* or "p-coumar*") OR AB (coumar* or "p-coumar*") OR SU (coumar* or "p-coumar*") |
| 75 | TI (caffeic*) OR AB (caffeic*) OR SU (caffeic*) |
| 76 | TI (ferulic*) OR AB (ferulic*) OR SU (ferulic*) |
| 77 | TI (sinapic*) OR AB (sinapic*) OR SU (sinapic*) |
| 78 | TI (chlorogenic*) OR AB (chlorogenic*) OR SU (chlorogenic*) |
| 79 | TI (lignan*) OR AB (lignan*) OR SU (lignan*) |
| 80 | MH "Lignans" |
| 81 | TI (secoisolariciresinol*) OR AB (secoisolariciresinol*) OR SU (secoisolariciresinol*) |
| 82 | TI (pinoresinol*) OR AB (pinoresinol*) OR SU (pinoresinol*) |
| 83 | TI (lariciresinol*) OR AB (lariciresinol*) OR SU (lariciresinol*) |
| 84 | TI (syrigaresinol*) OR AB (syrigaresinol*) OR SU (syrigaresinol*) |
| 85 | TI (matairesinol*) OR AB (matairesinol*) OR SU (matairesinol*) |
| 86 | TI (hydroxymatairesinol*) OR AB (hydroxymatairesinol*) OR SU (hydroxymatairesinol*) |
| 87 | TI (sesamin*) OR AB (sesamin*) OR SU (sesamin*) |
| 88 | MH "Stilbenes" |
| 89 | TI (stilbenoid* or stilbene* or stillbenoid* or stillbene*) OR AB (stilbenoid* or stilbene* or stillbenoid* or stillbene*) OR SU (stilbenoid* or stilbene* or stillbenoid* or stillbene*) |
| 90 | MH "Resveratrol" |
| 91 | TI (resveratrol) OR AB (resveratrol) OR SU (resveratrol) |
| 92 | TI (pterostilben*) OR AB (pterostilben*) OR SU (pterostilben*) |
| 93 | MH "Tannins" |
| 94 | TI (tannin* or tannic) OR AB (tannin* or tannic) OR SU (tannin* or tannic) |
| 95 | TI (gallotannin* or ellagitannin*) OR AB (gallotannin* or ellagitannin*) OR SU (gallotannin* or ellagitannin*) |
| 96 | (MH "Green Tea") |
| 97 | TI ((tea N5 extract) or "green tea" or Camelia or sinensis) OR AB ((tea N5 extract) or "green tea" or Camelia or sinensis) OR SU ((tea N5 extract) or "green tea" or Camelia or sinensis) |
| 98 | MH "Curcumin" |
| 99 | TI (curcumin*) OR AB (curcumin*) OR SU (curcumin*) |
| 100 | TI (turmeric or curcuma or longa) OR AB (turmeric or curcuma or longa) OR SU (turmeric or curcuma or longa) |
| 101 | MH "Grape Seed Extract" |
| 102 | MH "Grape" |
| 103 | TI (grape or grapes or GSE or Vitis or vinifera or labrusca or vinifera) OR AB (grape or grapes or GSE or Vitis or vinifera or labrusca or vinifera) OR SU (grape or grapes or GSE or Vitis or vinifera or labrusca or vinifera) |
| 104 | TI (mango or mangoes or mangifera*) OR AB (mango or mangoes or mangifera*) OR SU (mango or mangoes or mangifera*) |
| 105 | TI (mangiferin*) OR AB (mangiferin*) OR SU (mangiferin*) |
| 106 | MH "Moringa oleifera" |
| 107 | TI (Moringa or oleifera or drumstick or horseradish or "horse-radish" or "ben oil" or "benzolive") OR AB (Moringa or oleifera or drumstick or horseradish or "horse-radish" or "ben oil" or "benzolive") OR SU (Moringa or oleifera or drumstick or horseradish or "horse-radish" or "ben oil" or "benzolive") |
| 108 | MH "Pomegranate" |
| 109 | TI (pomegranate* or Punica or granatum) OR AB (pomegranate* or Punica or granatum) OR SU (pomegranate* or Punica or granatum) |
| 110 | TI (punicalagin*) OR AB (punicalagin*) OR SU (punicalagin*) |
| 111 | MH "Scutellaria baicalensis" |
| 112 | TI (Scutellaria or baicalensis or skullcap or "Huang Qin") OR AB (Scutellaria or baicalensis or skullcap or "Huang Qin") OR SU (Scutellaria or baicalensis or skullcap or "Huang Qin") |
| 113 | TI (baicalin*) OR AB (baicalin*) OR SU (baicalin*) |
| 114 | TI (oroxylin*) OR AB (oroxylin*) OR SU (oroxylin*) |
| 115 | MH "Salvia miltiorrhiza" |
| 116 | TI ("red sage" or "Chinese sage" or "tan shen" or tanshen or "dan shen" or danshen or Salvia or miltiorrhiza) OR AB ("red sage" or "Chinese sage" or "tan shen" or tanshen or "dan shen" or danshen or Salvia or miltiorrhiza) OR SU ("red sage" or "Chinese sage" or "tan shen" or tanshen or "dan shen" or danshen or Salvia or miltiorrhiza) |
| 117 | TI (Salvianolic*) OR AB (Salvianolic*) OR SU (Salvianolic*) |
| 118 | MH "Silymarin" |
| 119 | TI ("milk thistle" or Silybum or marianum or silymarin) OR AB ("milk thistle" or Silybum or marianum or silymarin) OR SU ("milk thistle" or Silybum or marianum or silymarin) |
| 120 | MH "Milk thistle" |
| 121 | TI (silibinin* or silybin*) OR AB (silibinin* or silybin*) OR SU (silibinin* or silybin*) |
| 122 | MH "Cruciferous Vegetables" |
| 123 | TI (Brassica* or Crucifer* or oleracea) OR AB (Brassica* or Crucifer* or oleracea) OR SU (Brassica* or Crucifer* or oleracea) |
| 124 | TI (broccoli or kale or "brussel sprout*") OR AB (broccoli or kale or "brussel sprout*") OR SU (broccoli or kale or "brussel sprout*") |
| 125 | TI (dill or dills or Anethum or graveolens) OR AB (dill or dills or Anethum or graveolens) OR SU (dill or dills or Anethum or graveolens) |
| 126 | MH "Berries+" |
| 127 | (TI (berry or berries) OR AB (berry or berries) OR SU (berry or berries)) |
| 128 | (TI (blueberr* or chokeberr* or cranberr* or gooseberr* or lingonberr* or whortleberr* or strawberr* or raspberr* or blackberr* or blackcurrant* or mulberr* or cloudberr*) OR AB (blueberr* or chokeberr* or cranberr* or gooseberr* or lingonberr* or whortleberr* or strawberr* or raspberr* or blackberr* or blackcurrant* or mulberr* or cloudberr*) OR SU (blueberr* or chokeberr* or cranberr* or gooseberr* or lingonberr* or whortleberr* or strawberr* or raspberr* or blackberr* or blackcurrant* or mulberr* or cloudberr*)) and S1 |
| 129 | TI (Piptadenia or stipulacea*) OR AB (Piptadenia or stipulacea*) OR SU (Piptadenia or stipulacea*) |
| 130 | TI ("galetin-3,6-dimethyl ether" or fgal) OR AB ("galetin-3,6-dimethyl ether" or fgal) OR SU ("galetin-3,6-dimethyl ether" or fgal) |
| 131 | TI (magnolia) OR AB (magnolia) OR SU (magnolia) |
| 132 | TI (honokiol* or houpa) OR AB (honokiol* or houpa) OR SU (honokiol* or houpa) |
| 133 | MH "Olive oil" |
| 134 | (TI ("olive oil") OR AB ("olive oil") OR SU ("olive oil")) |
| 135 | MH "Olive" |
| 136 | TI (olea or europaea) OR AB (olea or europaea) OR SU (olea or europaea) |
| 137 | TI (hydroxytyrosol*) OR AB (hydroxytyrosol*) OR SU (hydroxytyrosol*) |
| 138 | MH "Citrus+" |
| 139 | (TI (citrus) OR AB (citrus) OR SU (citrus)) |
| 140 | TI (nobiletin*) OR AB (nobiletin*) OR SU (nobiletin*) |
| 141 | TI (diosmin*) OR AB (diosmin*) OR SU (diosmin*) |
| 142 | MH "Cacao" |
| 143 | (TI (chocolate) OR AB (chocolate) OR SU (chocolate)) |
| 144 | MH "Wine" |
| 145 | (TI (wine) OR AB (wine) OR SU (wine)) |
| 146 | S30 OR S31 OR S32 OR S33 OR S34 OR S35 OR S36 OR S37 OR S38 OR S39 OR S40 OR S41 OR S42 OR S43 OR S44 OR S45 OR S46 OR S47 OR S48 OR S49 OR S50 OR S51 OR S52 OR S53 OR S54 OR S55 OR S56 OR S57 OR S58 OR S59 OR S60 OR S61 OR S62 OR S63 OR S64 OR S65 OR S66 OR S67 OR S68 OR S69 OR S70 OR S71 OR S72 OR S73 OR S74 OR S75 OR S76 OR S77 OR S78 OR S79 OR S80 OR S81 OR S82 OR S83 OR S84 OR S85 OR S86 OR S87 OR S88 OR S89 OR S90 OR S91 OR S92 OR S93 OR S94 OR S95 OR S96 OR S97 OR S98 OR S99 OR S100 OR S101 OR S102 OR S103 OR S104 OR S105 OR S106 OR S107 OR S108 OR S109 OR S110 OR S111 OR S112 OR S113 OR S114 OR S115 OR S116 OR S117 OR S118 OR S119 OR S120 OR S121 OR S122 OR S123 OR S124 OR S125 OR S126 OR S127 OR S128 OR S129 OR S130 OR S131 OR S132 OR S133 OR S134 OR S135 OR S136 OR S137 OR S138 OR S139 OR S140 OR S141 OR S142 OR S143 OR S144 OR S145 |
| 147 | TI ((mouse or rat or rats or mice or "in vitro") not (clinical or human*)) |
| 148 | TI (editorial or "case report*" or "case stud*" or commentary) |
| 149 | (MH "Animal studies" or MH "Animals") NOT (MH "Clinical research" or MH "Human") |
| 150 | PT "ANECDOTE" OR PT "BOOK" OR PT "BOOK CHAPTER" OR PT "CASE STUDY" OR PT "COMMENTARY" OR PT "EDITORIAL" OR PT "LETTER" OR PT "QUESTIONS & ANSWERS" OR PT "RESPONSE" OR PT "STANDARDS" OR PT "TEACHING MATERIALS" OR PT "WEBSITE" |
| 151 | S147 OR S148 OR S149 OR S150 |
| 152 | (S11 or S29) and S146 not S151 |

**International Clinical Trials Registry Platform <last date of search: 11^th^ August, 2023)**

| Pregnancy | pregnan* or prenatal or "pre-natal" or perinatal or "peri-natal" or peripartum or "peri-partum" or antenatal or "ante-natal" or preeclamp* or "pre-eclamp*" or eclamp* or hellp or toxemi* or toxaemi* or obstetric* or maternal or maternity or childbirth or birth or "fetal growth" or "foetal growth" or preterm or premature or "foetal distress" or "fetal distress" or "foetal hypoxia" or "fetal hypoxia" or "gestation* diabete*" |
| --- | --- |
| Polyphenols | polyphenol* or flavon* or quercetin or rutin or rutoside or sophorin or kaempferol* or myricetin* or benzoflavo* or apigenin* or luteolin* or vitexin* or isoflavo* or daidzein* or genistein* or glycitein* or flavan* or catechin* or catechol or catechols or epicatechin* or epigallocatechin* or ecgc or hesperidin* or hesperetin* or narigenin* or eriodictyol* or proanthocyanid* or anthocyani* or cyanid* or delphinid* or malvedin* or pelargonidin* or phenolic* or hydroxybenzo* or "p-hydroxybenzo*" or gallic* or gallate* or "proto-catechuic*" or protocatechuic* or catechiuc* or vanillic* or ellagic* or hydroxycinnam* or coumar* or "p-coumar*" or caffeic* or ferulic* or sinapic* or chlorogenic* or lignan* or secoisolariciresinol* or pinoresinol* or lariciresinol* or syrigaresinol* or matairesinol* hydroxymatairesinol* or sesamin* or stilbenoid* or stilbene* or stillbenoid* or stillbene* or resveratrol or pterostilben* or tannin* or tannic or gallotannin* or ellagitannin* or (tea adj5 extract) or "green tea" or Camelia or sinensis or curcumin* or turmeric or curcuma or longa or grape or grapes or GSE or Vitis or vinifera or labrusca or vinifera or mango or mangoes or mangifera* or mangiferin* or Moringa or oleifera or drumstick or horseradish or "horse-radish" or "ben oil" or "benzolive" or pomegranate* or Punica or granatum or punicalagin* or Scutellaria or baicalensis or skullcap or "Huang Qin" or baicalin* or oroxylin* or "red sage" or "Chinese sage" or "tan shen" or tanshen or "dan shen" or danshen or Salvia or miltiorrhiza or salvianolic* or "milk thistle" or Silybum or marianum or silymarin or silibinin* or silybin* or Brassica* or Crucifer* or oleracea or broccoli or kale or "brussel sprout*" or dill or dills or Anethum or graveolens or berry or berries or blueberr* or chokeberr* or cranberr* or gooseberr* or lingonberr* or whortleberr* or strawberr* or raspberr* or blackberr* or blackcurrant* or mulberr* or cloudberr* or Piptadenia or stipulacea* or "galetin-3,6-dimethyl ether" or fgal or magnolia or honokiol* or houpa or olea or europaea or "olive oil" or hydroxytyrosol* or citrus or nobiletin* or diosmin* or chocolate or wine |

Note: The search components were combined into the full search strategy as (pregnancy AND polyphenols) AND (polyphenols). Due to the interface’s restriction, the full search strategy was broken down into small searches.

**S4. List of outcomes for meta-analysis**

| **Category** | **Prevention Outcomes**  **(Participants without preeclampsia)** | **Treatment Outcomes**  **(Participants with preeclampsia)** |
| --- | --- | --- |
| **PRIMARY OUTCOMES** | | |
| Maternal | - Incidence of preeclampsia | - Maternal death - Incidence of eclampsia - Progression to severe preeclampsia, eclampsia and/or HELLP syndrome |
| Fetal & newborn | - Fetal loss - stillbirth (pregnancy loss from 20^th^ gestational weeks onwards) - miscarriage (pregnancy loss before 20^th^ gestational weeks) - intrapartum death (death of fetus during labor) - Neonatal death - Perinatal mortality (composite of fetal death and early neonatal death) - Neonatal mortality (death of liveborn neonate within 28 days of life) - Infant or childhood death, including sudden infant death syndrome - Gestational age at birth - Birth weight - Low birth weight (birth weight <2500g) - Small-for-gestational age (birth weight <10th percentile) - Admission to neonatal intensive care unit and/or respiratory support | Same as Prevention outcomes |
| **SECONDARY OUTCOMES** | | |
| Maternal | - Maternal complications, including but not limited to: - Stroke - Pulmonary edema - Cortical blindness - Retinal detachment - HELLP diagnosis - Acute kidney injury - Renal failure - Liver capsule hematoma/rupture - Raised liver enzymes - Proteinuria - Liver failure - Low platelets - Placental abruption - Postpartum hemorrhage - ICU admission - Intubation/mechanical ventilation (not for childbirth) - Any composite measure of maternal morbidity, including any of the above or otherwise defined by authors - Adverse effects, including but not limited to: - Mild: diarrhea, stomach pain, nausea & vomiting - Severe: hypotension, dizziness, nephropathy | Same as Prevention outcomes  and   - Preeclampsia severity - Clinical improvement (resolution or reduction of clinical symptoms, decrease in blood pressure) - Time to achieve clinical improvement - Time to next hypertensive crisis - Blood pressure (systolic, diastolic, mean arterial) - Increase in gestational length |
| Fetal & newborn | - Preterm birth (<37 weeks, <34 weeks, or <28 weeks of gestation) - Intrauterine growth restriction - Apgar score at 5min - Low Apgar score - Neonatal complications, including but not limited to: - Respiratory distress syndrome - Neonatal sepsis - Necrotizing enterocolitis - Retinopathy of prematurity - Neonatal asphyxia - Meconium-stained amniotic fluid - Fetal distress - Neonatal seizures - Any composite measure of neonatal morbidity, including any of the above or otherwise defined by authors - Congenital defects, including but not limited to: ductus arteriosus constriction, heart defects, neural tube defects, spina bifida. | Same as Prevention outcomes |
| Biomarkers | Maternal serum levels of:   - Soluble fms-like tyrosine kinase 1 (sFlt-1) - Pregnancy-associated plasma protein A (PAPP-A) - Placental growth factor (PIGF) - Soluble endoglin (sEng) - Endothelin-1 (ET-1 or PPET1) | Same as Prevention outcomes |

*Note: Where outcomes were not clearly defined by trial authors, clinical definitions provided in the brackets were used to determine whether the outcome was eligible for meta-analysis.*

**S5. List of excluded full-texts with reasons**

| **First author & year** | **Title** | **Reason for exclusion** |
| --- | --- | --- |
| Haghshenosabet 2022 | Association between pre-pregnancy food group intakes and hyperemesis gravidarum: a case-control study | Full texts not available |
| Heo 2016 | Effects of Korean herbal medicine on pregnancy outcomes of infertile women aged over 35: A retrospective study | Full texts not available |
| Izadpanah 2018 | Effect of grape seed powder on postpartum hemorrhage in vaginal delivery: a randomized controlled clinical trial | Full texts not available |
| Kang 2017 | Influence of oral probiotic and cranberry capsules on vagina flora in pregnant women with group B streptococcus colonization | Full texts not available |
| Laforet 1969 | Action of trioxyethylrutin on capillary resistance and venous disorders in pregnant women | Full texts not available |
| Liu 1994 | The effects of Salvia miltiorrhizae Bge and Ligustrazine on thromboxane A2 and prostacyclin in pregnancy induced hypertension | Full texts not available |
| Mutmaina 2021 | The effect of giving moringa honey towards hemoglobin levels and erythrocyte index in pregnant women with anemia at the Turikale & lau health center, maros regency | Full texts not available |
| Neuhauser 1975 | Fetal damage through coumarins | Full texts not available |
| Nurdin 2018 | The effect of moringa leaf extract and powder to haemoglobin concentration among pregnant women in jeneponto regency | Full texts not available |
| Rankin 2014 | Potential effects of chocolate on human pregnancy: a randomised controlled trial | Full texts not available |
| Serment 1979 | Trial of a mixture of rutoside with ascorbic acid and adenoside in pregnant women | Full texts not available |
| Sohn 1995 | Effectiveness of beta-hydroxyethylrutoside in patients with varicose veins in pregnancy | Full texts not available |
| Suboohi 2008 | Clinical efficacy of Joshanda Mulayyan' in the treatment of constipation during pregnancy | Full texts not available |
| Trillo 2011 | Effectiveness of raspberry leaf extract in labor induction | Full texts not available |
| Ushiroyama 2006 | Efficacy of the kampo medicine xiong-gui-jiao-AI-tang, a traditional herbal medicine, in the treatment of threatened abortion in early pregnancy | Full texts not available |
| Virtanen 1994 | Is children's or parents' coffee or tea consumption associated with the risk for type 1 diabetes mellitus in children? | Full texts not available |
| Vohra 2002 | Randomised clinical trial of raspberry leaf herb in pregnancy to determine the impact on labour and safety | Full texts not available |
| Wang 2006 | Research and discuss of Leishi Danshen Tablet in treating pregnancy-induced hypertension syndrome | Full texts not available |
| Westphal 2006 | Double-blind, placebo-controlled study of Fertilityblend: a nutritional supplement for improving fertility in women | Full texts not available |
| Fang 2016 | Effects of quercetin and melatonin in pregnant and gestational diabetic women | Ineligible control - Active control |
| Hadju 2020 | Moringa oleifera leaf powder supplementation improved the maternal health and birth weight: A randomised controlled trial in pregnant women | Ineligible control - Active control |
| Hastuti 2020 | The Effect of Moringa oleifera on Pregnant Women and Breastfeeding Mothers toward Social-personal Development of Children Aged 18–23 Months in Jeneponto, South Sulawesi | Ineligible control - Active control |
| Simoes-Wust 2018b | Two Randomised Clinical Trials on the Use of Bryophyllum pinnatum in Preterm Labour: Results after Early Discontinuation | Ineligible control - Active control |
| Suhartatik 2020 | Affect of moringa oleifera given against pregnant and breastfeeding mothers cortisol | Ineligible control - Active control |
| Suhartatik 2020 | The effect of moringa oleifera flour given for mothers breastfeeding against morbidity of baby ages 0-6 months in Jeneponto District | Ineligible control - Active control |
| Ulmy 2020 | Effect of moringa leaves during pregnancy on growth and morbidity in 0–5 months | Ineligible control - Active control |
| DRKS 2022 | Castor oil cocktail for induction of labour: a randomized, controlled, double blind study to investigate its efficacy | Ineligible control - Control also received polyphenols |
| Dunstan 2004 | The effect of supplementation with fish oil during pregnancy on breast milk immunoglobulin A, soluble CD14, cytokine levels and fatty acid composition | Ineligible control - No control group |
| Gares 1966 | Use of citroflavonoids in the prevention of complications due to fetomaternal blood incompatibility | Ineligible control - No control group |
| Giannola 1985 | A two-center study of the effects of silymarine in pregnant women and adults with the so-called minor liver insufficiency syndrome | Ineligible control - No control group |
| Kiguba 2016 | Herbal medicine use and linked suspected adverse drug reactions in a prospective cohort of Ugandan inpatients | Ineligible control - No control group |
| Nili 2021 | Evaluation of the effect of rosa damascena mill. Product on constipation during pregnancy: A single-arm clinical trial | Ineligible control - No control group |
| Nishat 2017 | A comparative study of polyherbal Unani formulations in gestational urinary tract infections | Ineligible control - No control group |
| Yosali 2022 | The Effect of Banana and Strawberry Juice in Increasing Hemoglobin Levels in Pregnant Women with Anemia | Ineligible control - No control group |
| Bisanz 2015 | Microbiota at Multiple Body Sites during Pregnancy in a Rural Tanzanian Population and Effects of Moringa-Supplemented Probiotic Yogurt | Ineligible intervention - Including non-polyphenols |
| Li 2010 | Study on treatment of early onset of severe pre-eclampsia by salviae injection and ligustrazine injection in combining with Western medicine | Ineligible intervention - Including non-polyphenols |
| NCT05393843 2022 | PREDIP2 - Prevention of Maternal and Fetal Metabolic Complications With Polyphenols and Omega-3 Fatty Acids Supplementation in Pregnant Women Affected by Gestational Diabetes on Diet Therapy: a Randomized, Double-blind Placebo Controlled Trial. | Ineligible intervention - Including non-polyphenols |
| Westphal 2004 | A Nutritional Supplement for Improving Fertility in Women: A Pilot Study | Ineligible intervention - Including non-polyphenols |
| Baghbahadorani 2016 | The impact of Silymarin on improvement of platelet abnormalities in patients with severe preeclampsia | Ineligible intervention - Intervention given postpartum |
| Baghbahadorani 2017 | The Impact of Silymarin on Improvement of Hepatic Abnormalities in Patients with Severe Preeclampsia: A Randomized Clinical Trial | Ineligible intervention - Intervention given postpartum |
| Belcaro 2014 | Pycnogenol R in postpartum symptomatic hemorrhoids | Ineligible intervention - Intervention given postpartum |
| Bolou 2021 | Acceptability and adherence to a Mediterranean diet in the postnatal period to prevent type 2 diabetes in women with gestational diabetes in the UK: a protocol for a single-arm feasibility study (MERIT) | Ineligible intervention - Intervention given postpartum |
| Fazel 2017 | Effects of Anethum graveolens L. (Dill) essential oil on the intensity of retained intestinal gas, flatulence and pain after cesarean section: A randomized, double-blind placebo-controlled trial | Ineligible intervention - Intervention given postpartum |
| Shabani 2021 | The Effects of Silymarin and N-Acetylcysteine on Liver and Kidney Dysfunction in Subjects with Severe Pre-eclampsia | Ineligible intervention - Intervention given postpartum |
| Bergstein 1975 | Clinical study on the efficacy of O-(beta-hydroxyethyl)rutoside (HR) in varicosis of pregnancy | Ineligible intervention - Mode of administration not eligible |
| Khojastehfard 2021 | The effect of rectal suppository of anethum graveolens on the postpartum hemorrhage rate: Evidence from a single-blind clinical trial study | Ineligible intervention - Mode of administration not eligible |
| Mohammadi 2022 | The Impact of Aromatherapy with Citrus Aurantium Essential Oil on Sleep Quality in Pregnant Women with Sleep Disorders: A Randomized Controlled Clinical Trial | Ineligible intervention - Mode of administration not eligible |
| Taavoni 2012 | A Survey of the Effects of Olive Oil and Saj Cream on Striae Gravidarum in the Second Trimester of Pregnancy | Ineligible intervention - Mode of administration not eligible |
| Zahra 2022 | The effects of rectal suppositories of Plantago major and Anetheum Graveolens on postpartum hemorrhage: a randomized triple blinded clinical trial | Ineligible intervention - Mode of administration not eligible |
| Ainehchi 2019 | The effectiveness of herbal mixture supplements with and without clomiphene citrate in comparison to clomiphene citrate on serum antioxidants and glycemic biomarkers in women with polycystic ovary syndrome willing to be pregnant: A randomized clinical trial | Ineligible intervention - More than three ingredients |
| Bradley 2020 | Can broad-spectrum multinutrients treat symptoms of antenatal depression and anxiety and improve infant development? Study protocol of a double blind, randomized, controlled trial (the 'NUTRIMUM' trial) | Ineligible intervention - More than three ingredients |
| CTRI 2017 | Ayurvedic management in preventing intra uterine growth retardation | Ineligible intervention - More than three ingredients |
| Han 2021 | Effect of Yiqi Buxue decoction on hemodynamic changes of the uterine artery and fetal umbilical artery and pregnancy outcomes in pregnant patients with pulmonary arterial hypertension | Ineligible intervention - More than three ingredients |
| Li 2021 | Clinical evaluation of Pinggan Yiqi Yangshen recipe combined with labetalol hydrochloride and magnesium sulfate in the treatment of PIH | Ineligible intervention - More than three ingredients |
| Qian 1991 | Prediction and prevention of hypertension syndrome of pregnancy | Ineligible intervention - More than three ingredients |
| Wang 2021 | Clinical effects of integrated traditional Chinese and western medicine in treating severe preeclampsia and its influence on maternal and infant outcomes after cesarean section under combined lumbar and epidural anesthesia | Ineligible intervention - More than three ingredients |
| Jahan 2022 | Use of herbal medicines during pregnancy in a group of Bangladeshi women | Ineligible intervention - No clear product |
| Liao 2020 | Traditional Chinese medicine treatment associated with female infertility in Taiwan: a population-based case-control study | Ineligible intervention - No clear product |
| DRKS00007620 2015 | Investigation of embryotoxic effects of maternal therapy with vitamin k antagonist phenprocoumon in pregnancy: the experience of the Embryotox- database - Phenprocoumon in pregnancy | Ineligible intervention - Not polyphenols |
| IRCT2016092729902N3 2016 | The effect of low Trans Fatty Acid content diet on adverse pregnancy outcomes | Ineligible intervention - Not polyphenols |
| Olsen 2000 | Randomised clinical trials of fish oil supplementation in high risk pregnancies. Fish Oil Trials In Pregnancy (FOTIP) Team | Ineligible intervention - Not polyphenols |
| Soldavini 2022 | Maternal AA/EPA Ratio and Triglycerides as Potential Biomarkers of Patients at Major Risk for Pharmacological Therapy in Gestational Diabetes | Ineligible intervention - Not polyphenols |
| Sorensen 1993 | Effects of fish oil supplementation in the third trimester of pregnancy on prostacyclin and thromboxane production | Ineligible intervention - Not polyphenols |
| Sorensen 1994 | Effects of fish oil supplementation in late pregnancy on blood lipids, serum urate, coagulation and fibrinolysis. A randomised controlled study | Ineligible intervention - Not polyphenols |
| van Houwelingen 1995 | Essential fatty acid status in neonates after fish-oil supplementation during late pregnancy | Ineligible intervention - Not polyphenols |
| Capra 1972 | Use of flavoxate in the treatment of threatened abortion and threatened premature labor | Ineligible intervention - Pharmaceutical products |
| Hurault-Delarue 2017 | Exposure to troxerutin during pregnancy: Risk of congenital anomalies for the fetus? | Ineligible intervention - Pharmaceutical products |
| Laforet 1971 | Study of a new esculetol derivative in obstetrical gynecology | Ineligible intervention - Pharmaceutical products |
| Milchev 2008 | Using phlebodia in pregnant women during preterm delivery | Ineligible intervention - Pharmaceutical products |
| Murff 2021 | Investigating N-3 Fatty Acids to prevent Neonatal Tobacco-related outcomeS (INFANTS): study protocol for a double-blind, randomized, placebo-controlled parallel clinical trial of n-3 polyunsaturated fatty acids in pregnant smokers | Ineligible intervention - Pharmaceutical products |
| dela Torre 2019 | Effectiveness of Following Mediterranean Diet Recommendations in the Real World in the Incidence of Gestational Diabetes Mellitus (GDM) and Adverse Maternal-Foetal Outcomes: A Prospective, Universal, Interventional Study with a Single Group. The St Carlos | Ineligible intervention - Polyphenols as food/drink |
| Hillesund 2014 | Associations of adherence to the New Nordic Diet with risk of preeclampsia and preterm delivery in the Norwegian Mother and Child Cohort Study (MoBa) | Ineligible intervention - Polyphenols as food/drink |
| Inder 2023 | Maternal pomegranate juice provides fetal neuroprotection for intrauterine growth restriction | Ineligible intervention - Polyphenols as food/drink |
| Jochum 2017 | Mothers' Consumption of Soy Drink But Not Black Tea Increases the Flavonoid Content of Term Breast Milk: a Pilot Randomized, Controlled Intervention Study | Ineligible intervention - Polyphenols as food/drink |
| Kaminski 1978 | Alcohol consumption in pregnant women and the outcome of pregnancy | Ineligible intervention - Polyphenols as food/drink |
| Kaminski 1981 | Moderate alcohol use and pregnancy outcome | Ineligible intervention - Polyphenols as food/drink |
| Klebanoff 2009 | Maternal serum theobromine and the development of preeclampsia | Ineligible intervention - Polyphenols as food/drink |
| Kuzma 1982 | Maternal drinking behavior and decreased intrauterine growth | Ineligible intervention - Polyphenols as food/drink |
| Maijaliisa 2011 | Maternal food consumption during pregnancy and risk of asthma and allergic outcomes in the offspring | Ineligible intervention - Polyphenols as food/drink |
| Makelarski 2013 | Periconceptional maternal alcohol consumption and neural tube defects | Ineligible intervention - Polyphenols as food/drink |
| Marinho 2016 | Fetal ductus arteriosus constriction and maternal consumption of polyphenol-rich foods in late pregnancy: A 6 year experience in a portuguese tertiary-care hospital | Ineligible intervention - Polyphenols as food/drink |
| Matthews 2019 | Longitudinal effect of 20-year infancy-onset dietary intervention on food consumption and nutrient intake: the randomized controlled STRIP study | Ineligible intervention - Polyphenols as food/drink |
| Mikkelsen 2013 | Alcohol consumption and time to pregnancy: A Danish prospective cohort study | Ineligible intervention - Polyphenols as food/drink |
| NCT05694520 2023 | A Pragmatic Randomized Controlled Trial of Health Impact of Pistachios on Women With Gestational Diabetes Mellitus | Ineligible intervention - Polyphenols as food/drink |
| Paladine 2004 | Consuming less than 4 alcoholic drinks per week does not increase risk of pre-term delivery | Ineligible intervention - Polyphenols as food/drink |
| Virtanen 2011 | Maternal food consumption during pregnancy and risk of advanced Î²-cell autoimmunity in the offspring | Ineligible intervention - Polyphenols as food/drink |
| Zielinsky 2021 | Improvement in fetal pulmonary hypertension and maturity after reversal of ductal constriction: prospective cohort study | Ineligible intervention - Polyphenols as food/drink |
| IRCT20201209049666N 2021 | The Effect of Citrus Aurantium distillate on Anxiety and Sleep Disorder in mothers with neonate admitted to neonatal intensive care unit | Ineligible population - Infants |
| Mustapa 2020 | The effect of moringa oleifera to hemoglobin levels of preconception women in the health center tibawa district tibawa, gorontalo | Ineligible population - Non-pregnant women |
| Rakesh 2017 | A randomized active controlled clinical study to evaluate efficacy and safety of resveratrol as an adjuvant therapy in patients with hypertension | Ineligible population - Non-pregnant women |
| Salinas-Osornio 2021 | Capacidad antioxidante total de la dieta de las mujeres gestantes de la Comunidad de Madrid | Ineligible population - Non-pregnant women |
| Seifi 2018 | The effect of Rosa (L. Rosa canina) on the incidence of urinary tract infection in the puerperium: a randomized placebo-controlled trial | Ineligible population - Non-pregnant women |
| Huang 2022 | Dose-Effect Relationship of Chitosan and Danshen Combined Injection for Fallopian Tube Recanalization | Ineligible population - Women seeking treatment for infertility |
| NCT 2021 | Efficacy and Safety of Gushen Antai Pill on Ongoing Pregnancy Rate in Women With Normal Ovarian Reserve Undergoing IVF-ET | Ineligible population - Women seeking treatment for infertility |
| Vaez 2023 | Quercetin and polycystic ovary syndrome; inflammation, hormonal parameters and pregnancy outcome: A randomized clinical trial. | Ineligible population - Women seeking treatment for infertility |
| Wu 2019 | Intervention of Modified Yupingfeng San Combined with Aspirin on D-dimer， PagT Levels of RSA with Seronegative APS | Ineligible population - Women seeking treatment for infertility |
| Zhai 2009 | Clinical study on Salvia injection (freeze-dried) in the intervention treatment of the oviduct obstructive infertility | Ineligible population - Women seeking treatment for infertility |
| Dieckmann 1949 | Capillary fragility and the use of rutin in toxemias of pregnancy | Ineligible study design - Case series |
| Ozturk 2018 | Pregnancy outcomes in psychiatric patients treated with Passiflora incarnata | Ineligible study design - Case series |
| Harun 2023 | The Effect of Preconcenption Moringa Leaf Extract Supplementation on Cotinine Serum Levels in Pregnant Women Exposed to Tobacco Smoke | Ineligible study design - Cohort study |
| Rahma 2023 | The Effect of Moringa Leaf Extract Intervention Since Preconception Period on the Prevention of Oxidative Stress in Pregnant Women and Adverse Pregnancy Outcomes | Ineligible study design - Cohort study |
| Williford 2023 | Maternal dietary caffeine consumption and risk of birth defects in the National Birth Defects Prevention Study, 1997-2011. | Ineligible study design - Cohort study |
| Zhu 2023 | Prenatal exposures to isoflavones and neurobehavioral development in children at 2 and 4 years of age: A birth cohort study. | Ineligible study design - Cohort study |
| Derbo 2023 | The Effect of Fresh Moringa Leaf Consumption During Pregnancy on Maternal Hemoglobin Level in Southern Ethiopia: Multilevel Analysis of a Comparative Cross-Sectional Study. | Ineligible study design - Cross-sectional study |
| Nordeng 2011 | Use of herbal drugs during pregnancy among 600 Norwegian women in relation to concurrent use of conventional drugs and pregnancy outcomes | Ineligible study design - Cross-sectional study |
| Andreoli 1957 | Effects of stilbenes on the histaminolytic activity of blood in pregnant women | Ineligible study design - In vitro study |
| ACTRN12618001655235 2018 | Investigating the pharmacokinetic profile of broccoli sprout extract in women with preeclampsia. | Ineligible study design - Non-randomised CT |
| ACTRN12623000591651 2023 | Assessment of maternal-fetal transfer of sulforaphane and metabolites | Ineligible study design - Non-randomised CT |
| ACTRN12623000592640 2023 | Comparison of broccoli sprout extracts in healthy pregnant women | Ineligible study design - Non-randomised CT |
| Chen 2020 | Effects of magnesium sulfate combined with compound danshen injection on pregnancy outcome, vascular endothelia function, liver and kidney function in patients with EOSPE | Ineligible study design - Non-randomised CT |
| Fen 2012 | Analysis of the perinatal outcome of the early severe preeclampsia women treated with Compound Danshen Injection and low molecular weight heparin | Ineligible study design - Non-randomised CT |
| Jing 2019 | Effect of self-made traditional Chinese medicine decoction on pregnancy outcome in patients with hypertensive disorder complicating pregnancy | Ineligible study design - Non-randomised CT |
| Nur 2020 | The effect of moringa leaf extracton increasing hemoglobin and bodyweight in post-disaster pregnant women | Ineligible study design - Non-randomised CT |
| Tong 2012 | Clinical effectiveness assessment of compound danshen injection on early onset severe preeclampsia patients | Ineligible study design - Non-randomised CT |
| Tong 2015 | Effect of Low Molecular Weight Heparin Calcium Combined Compound Danshen Injection on Perinatal Outcomes of Nephrotic Syndrome Patients with Early Onset Severe Pre-eclampsia | Ineligible study design - Non-randomised CT |
| Wang 2003 | Ligustrazine and Salvia miltiorrhiza injection solution in complementary therapy of pregnancy-induced hypertension: clinical analysis of 60 cases | Ineligible study design - Non-randomised CT |
| Zhou 2022 | The effect of a traditional Chinese quadri-combination therapy and its component quercetin on recurrent spontaneous abortion: A clinical trial, network pharmacology and experiments-based study. | Ineligible study design - Non-randomised CT |

**S6. Ongoing trials and protocols without result**

| Trial registration number / First author | Year of publication | Title of trial / protocol | Intervention | Status / period of recruitment | Population | Eligible age / gestational age | List of outcomes eligible for review |
| --- | --- | --- | --- | --- | --- | --- | --- |
| Trials targeting preeclampsia | |  |  |  |  |  |  |
| [ACTRN 12618000216213](https://anzctr.org.au/Trial/Registration/TrialReview.aspx?id=373991) | 2021 | Prolong: a double-blind randomized placebo-controlled trial of broccoli sprout extract in women with early onset preeclampsia | Broccoli sprout extract, *Brassica oleracea* | Not yet recruiting  Jun 2023 – not specified | Pregnant women with early-onset preeclampsia | ≥18 years / 24+0 to 33+6 weeks | - Composite maternal morbidity (maternal death, eclampsia, HELLP syndrome and other pregnancy complications) - Composite neonatal morbidity (neonatal death, 5-min Apgar score <7, NICU admission and other neonatal complications) - Maternal serum levels of sFlt-1, soluble endoglin and placental growth factor - Preeclampsia severity, defined as escalation of antihypertensive therapy, increase in SBP and DBP, DIC, and severe renal, hepatic or hematological involvement, - Stillbirth - Gestational age at birth - Low birth weight (<5th percentile) |
| Trials targeting pregnant women in general | | | | | | | |
| [NCT 02425436](https://clinicaltrials.gov/ct2/show/NCT05364008) | 2015 | Role of Ginkgo Biloba Extract in IUGR | Ginkgo extract, *Ginkgo biloba* | Completed  Jan 2023 - Sep 2024 | Pregnant women with IUGR | 20-35 years / 28-30 weeks | none |
| [ACTRN 12622000173796](https://anzctr.org.au/Trial/Registration/TrialReview.aspx?id=383394) | 2022 | CO-Sprout: A double-blinded randomized control trial of broccoli sprout powder supplementation for pregnant women with COVID-19 on the rate of hospital admission | Broccoli sprout extract, *Brassica oleracea* | Recruiting  Aug 2022 - Dec 2023 | Pregnant, unvaccinated women with positive COVID-19 tests | ≥18 years /  20-36 weeks | - Maternal death - Composite severe neonatal morbidity (intrauterine fetal death, neonatal death, NICU admission and other neonatal complications) - Gestational age at birth |
| [IRCT 20210926052594N1](http://en.irct.ir/trial/59048) | 2021 | Citrus Aurantium and anxiety in the last month of pregnancy | Bitter orange blossom extract, *Citrus aurantium* | Completed  Oct 2021 - Nov 2021 | Pregnant women | 18-35 years / >36 weeks | none |
| [NCT 01584323](https://clinicaltrials.gov/ct2/show/NCT01584323) | 2012 | Pomegranate to reduce maternal and fetal oxidative stress and improve outcome in pregnancies complicated with preterm premature rupture of the membranes | Pomegranate, *Punica granatum* | Recruitment likely completed  Apr 2012 - May 2015 | Pregnant women with PPROM | 17-45 years / >24 weeks | Apgar score |
| [NCT 01818180](https://clinicaltrials.gov/ct2/show/NCT01818180) | 2013 | Effect of URELL (Cranberry Vaccinium Macrocarpon) Consumption on the Prevalence of Recurrent Urinary Tract Infection and Asymptomatic Bacteriuria During Pregnancy. | Cranberry supplement, *Vaccinium macrocarpon* | Terminated  May 2010 - Sep 2012 | Pregnant women with UTI | 18-40 years /  >4 months | none |
| [Silió Salas et al.](https://dialnet.unirioja.es/servlet/articulo?codigo=6266572) | 2015 | Effectiveness of urine infection prevention treatment with cranberry in pregnant women | Cranberry extraction, *Vaccinium macrocarpon* | Not specified | Pregnant women | ≥18 years /  <12 weeks | - Preeclampsia - Preterm birth |
| [Amador-Mulero et al.](https://www.researchgate.net/publication/286578978_Effectiveness_of_red_cranberries_ingestion_on_urinary_tract_infections_in_pregnant_women) | 2014 | Effectiveness of red cranberries ingestion on urinary tract infections in pregnant women | Cranberry extraction, *Vaccinium macrocarpon* | Not specified | Primigravida | Not specified | none |

**Abbreviation: DIC: disseminated intravascular coagulation; HELLP: hemolysis, elevated liver enzymes and low platelets; NICU: neonatal intensive care unit; PGF: placental growth factor; SBP/DBP: systolic/diastolic blood pressure; sFlt-1: soluble fms-like tyrosine kinase-1*

**S8. Flowchart of study screening and selection**

**
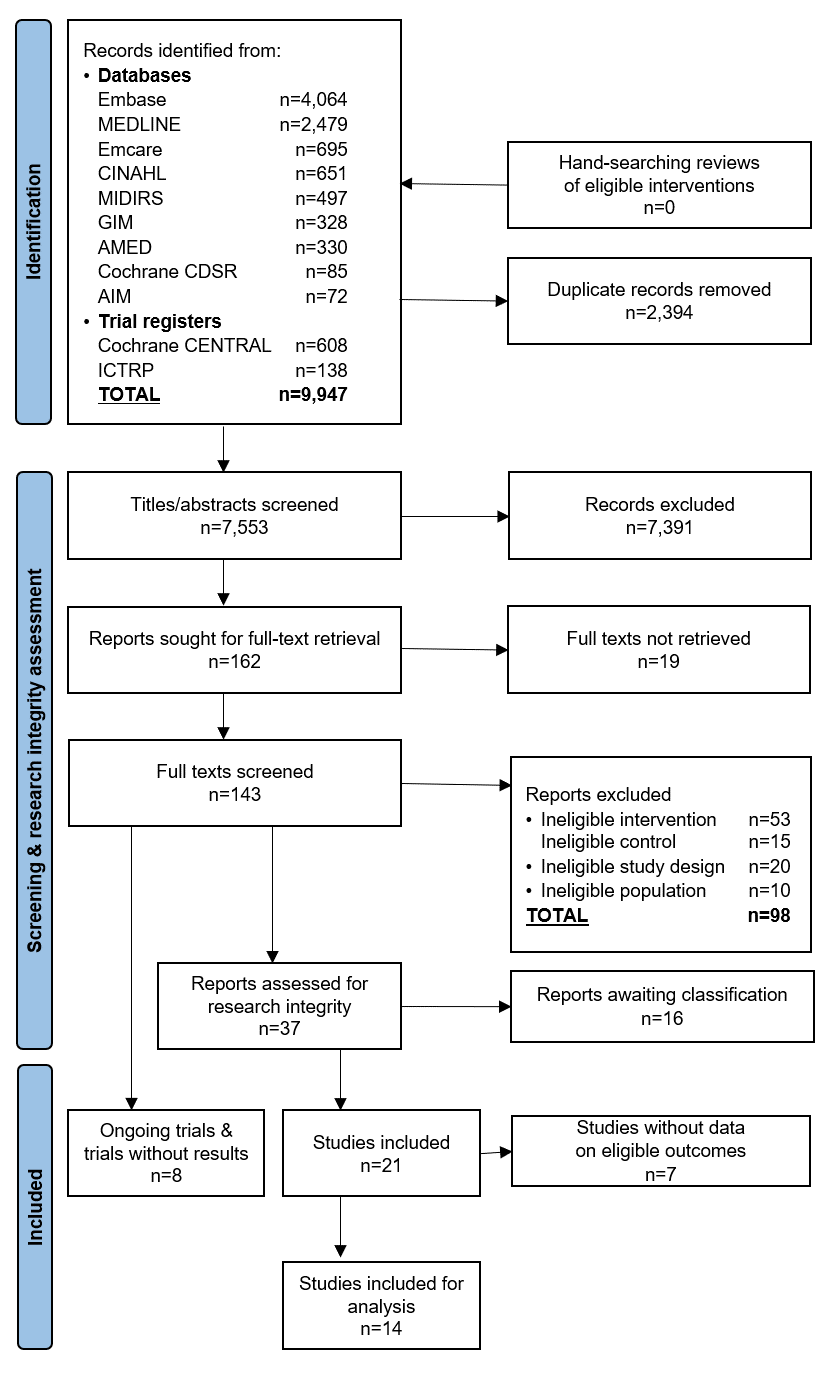
**

**S9. Study characteristics of trials not included in analysis**

**9.1. Trials awaiting classification after research integrity assessment**

| Study | Country (income group) | Funding | Participants | Baseline gestational age (weeks) | Pre-existing HDP | Risk factors of participants | Numbers randomized (intervention: control) | Intervention group | Control group | Duration |
| --- | --- | --- | --- | --- | --- | --- | --- | --- | --- | --- |
| Andira 2020 (9) | Indonesia (LMIC) | Not reported | Pregnant women | 20-32 weeks [range] | N | Anemia | 42 (21:21) | 1 capsule of 500mg *Moringa oleifera* daily, orally as extract | Placebo orally, once daily | 60 days |
| Arundhana 2018 (10) | Indonesia (LMIC) | Not reported | Pregnant women | I1: 17;  I2: 18;  C: 17 [mean] | N | Not reported | 453 (301:152) | 500mg *M. oleifera* daily, orally as extract or powder | 50mg iron/0.2mg folic acid orally daily | 90 days or 12 weeks |
| Basri 2021 (11) | Indonesia (LMIC) | Government | Pregnant women | Not reported | N | Not reported | 328 (112:114:102) | 500mg *M. oleifera* daily, orally as extract or powder | 60mg iron/0.2mg folic acid orally daily | 90 days |
| Chen 2003 (12) | China (UMIC) | Government | Pregnancy with PIH | I: 36;  C: 36 [mean] | Y | Not reported | 125 (75:50) | 8mL of danshen (*Salvia miltiorrhiza*) in 500mL of 5% glucose for infusion once daily, dosage not reported | 60mL of 25% magnesium sulfate injection in 1000mL of 5% glucose solution for infusion, 1-2g/h and 20-25g/24h | 7-10 days |
| Hastuty 2020 (13) | Indonesia (LMIC) | Not reported | Pregnant women | I: 27;  C: 26 [mean] | N | Anemia | 42 (21:21) | *M. oleifera* 250-500mg + 60mg iron once daily | Placebo + 60mg iron once daily | 60 days |
| Li 2009 (14) | China (UMIC) | Not reported | Pregnancy with IUGR | I: 25;  C: 26 [mean] | N | IUGR | 106 (53:53) | 20g of danshen (*S. miltiorrhiza*) in 500mL of 10% glucose solution for infusion + 500mL compound amino acids injection | 500mL compound amino acids injection | 7 days x 3 courses |
| Mandasari 2020 (15) | Indonesia (LMIC) | Not reported | Pregnant women | I: 27;  C: 26 [mean] | N | Anemia | 42 (21:21) | 1 capsule of 500mg *Moringa oleifera* daily, orally as extract | 1 iron tablet (unknown dose) once daily | 60 days |
| Miraj 2016 (16) | Iran (LMIC) | Not reported | Pregnancy with severe preeclampsia | 35-42 weeks [range] | Y | Blood pressure >140/90mmHg | 60 (30:30) | 70mg of silymarin, 3h and 24h after termination of pregnancy | Placebo, 3h and 24h after termination of pregnancy | 24 hours |
| Nadimin 2019 (17) | Indonesia (LMIC) | Other | Pregnant women | Not reported | N | None | 70 (35:35) | 800mg *M. oleifera* x 2 capsules daily | 60mg iron/0.25mg folic acid x 1 tablet daily | 12 weeks |
| Nadimin 2020 (18) | Indonesia (LMIC) | Other | Pregnant women | Not reported | N | None | 70 (35:35) | 800mg *M. oleifera* x 2 capsules daily | 60mg iron/0.25mg folic acid x 1 tablet daily | 12 weeks |
| Pawłowicz 2000 (19) | Spain (HIC) | Not-for-profit & academic; For-profit | Pregnancy with hypertension-induced IUGR | Not reported | Y | IUGR | 105 (50:55) | 1 tab of 100mg anthocyanins orally, 3 times daily | Placebo orally, 3 times daily | 2 months |
| Shen 2020 (20) | China (UMIC) | Government; Not-for-profit & academic | Pregnancy with IUGR | I: 32+2;  C: 34+3 [median] | N | None | 38 (19:19) | 20g danshen (*S. miltiorrhiza*), ATP 40 mg and CoA 200mg in 500mL of 5% glucose for infusion once daily | ATP 40 mg and CoA 200mg in 500mL of 5% glucose for infusion once daily | 7 days |
| Yang 2018 | China (UMIC) | No funding (confirmed by author) | Pregnancy with severe preeclampsia | Early onset: I: 32, C: 31 Late onset: I: 37, C: 38 [mean] | Y | History of preeclampsia/ eclampsia | 500 (250:250) | 10-20mL of Danshen (*S. miltiorrhiza*) in 250mL of 5% glucose solution for infusion Magnesium sulfate 5g in 20mL of 5% glucose solution for infusion for 30min, then 15g in 500mL of 5% glucose solution for infusion at 1-2g/h; total 25-30g daily | Magnesium sulfate 5g in 20mL of 5% glucose solution for infusion for 30min, then 15g in 500mL of 5% glucose solution for infusion at 1-2g/h; total 25-30g daily | 10 days |
| Zakiah 2020 (21) | Indonesia (LMIC) | Not reported | Pregnant women | 20-32 weeks [range] | N | Anemia | 30 (15:15) | 1 capsule of 500mg *Moringa oleifera* daily, orally as extract | Placebo orally, once daily | 60 days |
| Zhao 2021 (22) | China (UMIC) | Not reported | Pregnancy with PIH | I: 36; C: 35 [mean] | Y | Not reported | 80 (40:40) | 10-20mL of danshen (*S. miltiorrhiza*)* in 100-500mL of 5% glucose for infusion once daily Magnesium sulfate 4-10mL in 5% glucose solution for infusion once daily | Magnesium sulfate 4-10mL in 5% glucose solution for infusion once daily | 10 days |
| Zhou 2022 (23) | China (UMIC) | Not reported | Pregnancy with severe preeclampsia | I: 30; C: 30 [mean] | Y | None | 60 (30:30) | 16mL of danshen (Salvia miltiorrhiza) in 250mL of 5% glucose solution for infusion daily 5g magnesium sulfate in 20mL of 5% glucose solution for infusion 30min, then 15g in 500mL of 5% glucose solution for 2h, max 30g in 24h | 5g magnesium sulfate in 20mL of 5% glucose solution for infusion 30min, then 15g in 500mL of 5% glucose solution for 2h, max 30g in 24h | 7 days |

*Abbreviations: ATP: adenosine triphosphate; CoA: co-enzyme A; HDP: hypertensive disorders of pregnancy; IUGR: intrauterine growth restriction; LMIC / UMIC / HIC: lower-middle / upper-middle / high income country*

**9.2. Trials without data on eligible outcomes**

| Study | Country (income group) | Funding | Participants | Baseline gestational age (weeks) | Pre-existing HDP | Risk factors of participants | Numbers randomized (intervention: control) | Intervention group | Control group | Duration |
| --- | --- | --- | --- | --- | --- | --- | --- | --- | --- | --- |
| El Aal 2017 (24) | Egypt (LMIC) | Not reported | Pregnancy with IUGR | I: 32;  C: 31 [mean] | N | IUGR | 226 (110:106) | *Ginkgo biloba* extract (Tebonina Forte 40mg) 2 tabs daily | Placebo 2 tabs daily | 6 weeks |
| Chu 2008 (25) | China (UMIC) | Not reported | Pregnancy with oligohydramnios | I: 32;  C: 21 [mean] | N | Oligohydramnios | 84 (43:41) | 30mL of danshen (*Salvia miltiorrhiza*) in 500mL of 5% glucose solution for infusion over 4-6h, once daily 10mg dexamethasone once daily if <34 weeks of gestation | 500mL of 5% glucose for infusion once daily 10mg dexamethasone once daily if <34 weeks of gestation | 3 days |
| Fadinie 2019 (26) | Indonesia (LMIC) | No funding | Pregnancy with preeclampsia | At term | Y | History of preeclampsia/ eclampsia | 47 (23:24) | 100mg curcumin orally | Placebo | Not reported |
| Fadinine 2020 (27) | Indonesia (LMIC) | No funding | Pregnancy with preeclampsia | At term | Y | History of preeclampsia/ eclampsia | 46 (23:23) | 100mg curcumin orally | Placebo | Not reported |
| Hekmatzadeh 2014 (28) | Iran (LMIC) | Not-for-profit & academic | Pregnant women in labor | I: 38; C: 39 [mean] | N | None | 105 (52:53) | 10g dill seeds (*Anethum graveolens*) in 100 cc of boiled water, consumed orally | Routine care | Once during active labor |
| Ma 2018 (29) | China (UMIC) | Not reported | Pregnancy with scarred uterus | Preconception | N | Not reported | 78 (46:32) | 10mg resveratrol orally once daily | Placebo, orally daily | 3 months |
| Sulistyowati 2022 (30) | Indonesia (LMIC) | No funding | Pregnant women with hypertension | Not reported | Y | Hypertension | 20 (10:10) | Nano-extracts of Moringa oleifera 500mg/day | Not reported | Not reported |

*Abbreviations: HDP: hypertensive disorders of pregnancy; IUGR: intrauterine growth restriction; LMIC / UMIC / HIC: lower-middle / upper-middle / high income country*

**S10. Characteristics of included trials**

| Study | Country (income group) | Participants | Gestational age | Numbers randomized (intervention : control) | Intervention group | Control group | Duration of treatments |  |
| --- | --- | --- | --- | --- | --- | --- | --- | --- |
| A. Women without preeclampsia | | | | | | | | |
| (i) Danshen (*Salvia miltiorrhiza*) injection | | | | | | | | |
| Fang 2009 (35) | China (UMIC) | Pregnancy with IHCOP | Treatment initiation: 34 weeks  Baseline: I: 34; C: 34 weeks [mean] | 128 (72:56) | 1g danshen (*Salvia miltiorrhiza*)* in 500mL of 10% glucose for infusion once daily + ursodeoxycholic acid 15mg/kg/day, divided in 3 oral doses daily | 500mL of 10% glucose solution for infusion once daily + ursodeoxycholic acid 15mg/kg/day, divided in 3 oral doses daily | 14 days |  |
| Lin 2010 (37) | China (UMIC) | Pregnancy with risk factors (age >=40, socio-economically disadvantaged, family history of HDP, heavy physical labor) | Treatment initiation: 14-16 weeks  Baseline: NR | 4,814 (1,607:3,207) | 60mg danshen (*S. miltiorrhiza*), 0.3g vitamin C, 30mg vitamin E, orally 3 times daily | 0.3g vitamin C, 30mg vitamin E, orally 3 times daily | Approx. 20 weeks (from 14-16 to 34-36 gestational weeks) |  |
| Tan 2000 (43) | China (UMIC) | Pregnancy with IUGR | Treatment initiation: NR  Baseline: 32-41 weeks [range] | 25 (14:11) | 14mL of danshen (*S. miltiorrhiza*) in 500mL of dextran solution for infusion | No intervention | 10 days |  |
| (ii) Epigallocatechin gallate extract | | | | | | | | |
| Zhang 2017 (45) | China (UMIC) | Pregnancy with diabetes, including GDM | Treatment initiation: after 29 weeks  Baseline: NR | 404 (202:202) | 500mg ECGC orally daily | Placebo | Not reported |  |
| (iii) Resveratrol supplement | | | | | | | | |
| Malvasi 2017 (38) | Italy (HIC) | Overweight pregnant women | Treatment initiation: NR  Baseline: 24-28 weeks | 69 (35:34) | 80mg resveratrol 80mg, 200mg myo-inositol, 500mg D-chiro-inositol per tablet; dosage and frequency not reported | 200mg myo-inositol, 500mg D-chiro-inositol per tablet; dosage and frequency not reported | Not reported |  |
| (iv) *Bryophyllum pinnatum* extract | | | | | | | | |
| Simões-Wüst 2018a (41) | Switzerland (HIC) | Pregnancy with risk factors for preterm birth | Treatment initiation: After 26 weeks  Baseline: I: 28; C: 28 weeks [mean] | 26 (13:13) | *Bryophyllum pinnatum* (33% tincture, equivalent to 40mg dried B. pinnatum matter per 1g tincture), 20 drops 5 times daily Standard tocolytics as needed | Placebo (1% ferric chloride solution) 20 drops 5 times daily Standard tocolytics as needed | Not reported |  |
| Simões-Wüst 2018b (41) | Switzerland (HIC) | Pregnancy with premature labor (Bishop score <5) | Treatment initiation: NR  Baseline: I: 29; C: 28 [mean] | 27 (14:13) | 1 tablet of *B. pinnatum* (35mg dried *B. pinnatum* matter in each 350mg tablet) every 15min during first hour, then 2 tablets every 6hr | Nifedipine 1x10mg capsule every 15 min during the first 1hr and 15min, followed by 1x60mg or 1x30mg tablet every 12 over the next 48h | 49 hours |  |
| (v) Raspberry (*Rubus idaeus*) extract | | | | | | | | |
| Simpson 2001 (42) | Australia (HIC) | Pregnancy with nulliparity | Treatment initiation: 32 weeks  Baseline: At term | 192 (96:96) | 2 tablets of 1.2g raspberry (*Rubus idaeus*) leaf extract (400 mg of 3:1 extract) daily | Placebo x 2 tablets daily | From 32^nd^ gestational week until labor |  |
| (vi) Cranberry (*Vaccinium macrocarpon*) extract | | | | | | | | |
| Wing 2015 (44) | USA (HIC) | Uncomplicated pregnancy | Treatment initiation: 12-16 weeks  Baseline: NR | 49 (24:25) | 16.25mg proanthocyanidine x 2 capsules daily | Placebo x 2 capsules daily | From 12-16 gestational weeks until delivery |  |
| B. Women with pre-existing preeclampsia or other HDPs | | | | | | | | |
| (i) Danshen (*Salvia miltiorrhiza*) injection | | | | | | | | |
| Lai 2009 (36) | China (UMIC) | Pregnancy with PIH | Treatment initiation: NR  Baseline: I: 37; C: 37 weeks [mean] | 54 (27:27) | 15mL of danshen (*S. miltiorrhiza*) in 500mL of 50g/L glucose solution for infusion once daily, 30-40 drops/min 20-25g magnesium sulfate per 24h | 20-25g magnesium sulfate per 24h | 5 days |  |
| Shao 2014 (39) | China (UMIC) | Pregnancy with preeclampsia | Treatment initiation: 26-34 weeks  Baseline: NR | 69 (33:36) | 20mL of danshen (*S. miltiorrhiza*) in 250mL of 5% glucose solution for infusion once daily Magnesium sulfate IV 2g/h, <30g per day + low molecular weight heparin 100U once daily | Magnesium sulfate IV 2g/h, <30g per day + low molecular weight heparin 100U once daily | 7 days + another 7 days if needed |  |
| Zheng 2015 (46) | China (UMIC) | Pregnancy with preeclampsia | Treatment initiation: NR  Baseline: I: 36; C: 36 weeks [mean] | 704 (352:352) | 1-1.5g of danshen (*S. miltiorrhiza*) in 500mL of 5% glucose solution for infusion once daily Magnesium sulfate 5g injection for 5-10min on 1st day, then 10g infusion at 1-2g/h daily Nifedipine 10mg orally 3 times daily | Magnesium sulfate 5g injection for 5-10min on 1st day, then 10g infusion at 1-2g/h daily Nifedipine 10mg orally 3 times daily | 3 days |  |
| (ii) Epigallocatechin gallate extract | | | | | | | | |
| Shi 2018 (40) | China (UMIC) | Pregnancy with severe preeclampsia | Treatment initiation: NR  Baseline: I: 38; C: 37 weeks [mean] | 304 (148:156) | 100mg ECGC + nifedipine 10mg orally every 15min, up to 5 doses | Placebo + 10mg nifedipine 10mg orally every 15min, up to 5 doses | Until blood pressure ≤150/100mmHg |  |
| (iii) Resveratrol supplement | | | | | | | | |
| Ding 2017 (34) | China (UMIC) | Pregnancy with severe preeclampsia | Treatment initiation: NR  Baseline: I: 35; C: 33 weeks [mean] | 349 (174:175) | 50mg resveratrol + 10mg nifedipine every 15min, up to 5 oral doses per day | Placebo + 10mg nifedipine every 15min, up to 5 oral doses per day | Until blood pressure ≤150/100mmHg |  |

Abbreviations: ECGC: epigallocatechin gallate; GDM: gestational diabetes mellitus; HDP: hypertensive disorders of pregnancy; HIC/UMIC/LMIC: high/upper-middle/lower-middle income; ICOP: intrahepatic cholestasis of pregnancy; IUGR: intrauterine growth restriction; MO: Moringa oleifera; NR: not reported; PIH: pregnancy-induced hypertension; USA: United States of America

** It is possible the compound danshen injection used in many trials also contains another ingredient called jiangxiang (Dalbergiae odoriferae), although we were unable to clarify with the* corresponding *authors of those trials.*

**S12. Summary of results – side effects**

| **Outcome** | | **Results from meta-analysis**^†^ | | **No. of participants (studies)** | | **Reference** | | **Quality of evidence (GRADE)** |  |
| --- | --- | --- | --- | --- | --- | --- | --- | --- | --- |
| **1. Danshen (*Salvia miltiorrhiza*)** | | | | | | | | | |
| **(i) Women with pre-existing preeclampsia or other HDPs** | | | | | | | | |  |
| Rash | RR 9.00 (0.49 to 166.54) ⇧ | | 704  (1 RCT) | | (46) | | ⊖︀⊕⊕⊖︀ VERY LOW | |  |
| **2. Epigallocatechin gallate extract** | | | | | | | | | |
| **(i) Women with pre-existing preeclampsia or other HDPs** | | | | | | | | |  |
| Chest pain | RR 1.05 (0.22 to 5.14) ⇧ | | 304  (1 RCT) | | (40) | | ⊖︀⊕⊕⊖︀ LOW | |  |
| Dizziness | RR 1.41 (0.32 to 6.17) ⇧ | | 304  (1 RCT) | | (40) | | ⊖︀⊕⊕⊖︀ LOW | |  |
| Headache | RR 0.84 (0.23 to 3.08) ⇩ | | 304  (1 RCT) | | (40) | | ⊖︀⊕⊕⊖︀ LOW | |  |
| Nausea | RR 1.19 (0.47 to 2.99) ⇧ | | 304  (1 RCT) | | (40) | | ⊖︀⊕⊕⊖︀ LOW | |  |
| Shortness of breath | Zero events in both groups | | 304  (1 RCT) | | (40) | | - | |  |
| Tachycardia | RR 1.05 (0.31 to 3.57) ⇧ | | 304  (1 RCT) | | (40) | | ⊖︀⊕⊕⊖︀ LOW | |  |
| Vomiting | RR 0.60 (0.18 to 2.02) ⇩ | | 304  (1 RCT) | | (40) | | ⊖︀⊕⊕⊖︀ LOW | |  |
| Hypotension | RR 0.34 (0.01 to 8.17) ⇩ | | 304  (1 RCT) | | (40) | | ⊖︀⊕⊕⊖︀ LOW | |  |
| **3. Resveratrol supplement** | | | | | | | | |  |
| **(i) Women with pre-existing preeclampsia or other HDPs** | | | | | | | | |  |
| Chest pain | RR 1.01 (0.14 to 7.06) ⇧ | | 349  (1 RCT) | | (34) | | ⊖︀⊕⊕⊖︀ LOW | |  |
| Dizziness | RR 0.50 (0.05 to 5.50) ⇩ | | 349  (1 RCT) | | (34) | | ⊖︀⊕⊕⊖︀ LOW | |  |
| Headache | RR 1.01 (0.21 to 4.91) ⇧ | | 349  (1 RCT) | | (34) | | ⊖︀⊕⊕⊖︀ LOW | |  |
| Nausea | RR 1.01 (0.39 to 2.62) ⇧ | | 349  (1 RCT) | | (34) | | ⊖︀⊕⊕⊖︀ LOW | |  |
| Shortness of breath | RR 0.34 (0.01 to 8.17) ⇩ | | 349  (1 RCT) | | (34) | | ⊖︀⊕⊕⊖︀ LOW | |  |
| Tachycardia | RR 1.51 (0.26 to 8.92) ⇧ | | 349  (1 RCT) | | (34) | | ⊖︀⊕⊕⊖︀ LOW | |  |
| Vomiting | RR 0.67 (0.24 to 1.84) ⇩ | | 349 | | (34) | | ⊖︀⊕⊕⊖︀ LOW | |  |
| Hypotension | RR 0.35 (0.01 to 8.56) ⇩ | | (1 RCT) | | (34) | | ⊖︀⊕⊕⊖︀ LOW | |  |
| **4. *Bryophyllum pinnatum* extract** | | | | | | | | | |
| **(i) Women without preeclampsia** | | | | | | | | | |
| Dizziness | RR 0.19 (0.01 to 3.54) ⇩ | | 27  (1 RCT) | | (41) | | ⊖︀⊕⊖︀⊖︀ VERY LOW | |  |
| Headache | RR 0.07 (0.00 to 1.15) ⇩ | | 27  (1 RCT) | | (41) | | ⊖︀⊕⊖︀⊖︀ VERY LOW | |  |
| Vomiting | RR 0.31 (0.01 to 6.98) ⇩ | | 29  (1 RCT) | | (41) | | ⊖︀⊕⊖︀⊖︀ VERY LOW | |  |
| **5. Raspberry (*Rubus idaeus*) extract** | | | | | | | | | |
| **(i) Women without preeclampsia** | | | | | | | | | |
| Bloating | RR 3.00 (0.12 to 72.73) ⇧ | | 192  (1 RCT) | | (42) | | ⊖︀⊕⊖︀⊖︀ VERY LOW | |  |
| Constipation | RR 9.00 (0.49 to 164.90) ⇧ | | 192  (1 RCT) | | (42) | | ⊖︀⊕⊖︀⊖︀ VERY LOW | |  |
| Diarrhea | RR 1.12 (0.45 to 2.79) ⇧ | | 192  (1 RCT) | | (42) | | ⊖︀⊕⊖︀⊖︀ VERY LOW | |  |
| Dizziness | RR 3.00 (0.12 to 72.73) ⇧ | | 192  (1 RCT) | | (42) | | ⊖︀⊕⊖︀⊖︀ VERY LOW | |  |
| Headache | RR 1.00 (0.06 to 15.76) | | 192  (1 RCT) | | (42) | | ⊖︀⊕⊖︀⊖︀ VERY LOW | |  |
| Heartburn | RR 3.00 (0.12 to 72.73) ⇧ | | 192  (1 RCT) | | (42) | | ⊖︀⊕⊖︀⊖︀ VERY LOW | |  |
| Nausea | RR 0.73 (0.31 to 1.73) ⇩ | | 192  (1 RCT) | | (42) | | ⊖︀⊕⊖︀⊖︀ VERY LOW | |  |
| Rash | RR 0.33 (0.01 to 8.08) ⇩ | | 192  (1 RCT) | | (42) | | ⊖︀⊕⊖︀⊖︀ VERY LOW | |  |
| Uterine tightening | RR 5.00 (0.24 to 102.79) ⇧ | | 192  (1 RCT) | | (42) | | ⊖︀⊕⊖︀⊖︀ VERY LOW | |  |
| Vomiting | RR 2.00 (0.38 to 10.66) ⇧ | | 192  (1 RCT) | | (42) | | ⊖︀⊕⊖︀⊖︀ VERY LOW | |  |
| **6. Cranberry (Vaccinium macrocarpon) extract** | | | | | | | | | |
| **(i) Women without preeclampsia** | | | | | | | | | |
| Constipation | RR 0.65 (0.19 to 2.22) ⇩ | | 39  (1 RCT) | | (44) | | ⊖︀⊕⊖︀⊖︀ VERY LOW | |  |
| Diarrhea | RR 3.86 (0.17 to 89.03) ⇧ | | 39  (1 RCT) | | (44) | | ⊖︀⊕⊖︀⊖︀ VERY LOW | |  |
| Heartburn | RR 2.59 (0.54 to 12.50) ⇧ | | 39  (1 RCT) | | (44) | | ⊖︀⊕⊖︀⊖︀ VERY LOW | |  |
| Loss of appetite | RR 1.29 (0.30 to 5.63) ⇧ | | 39  (1 RCT) | | (44) | | ⊖︀⊕⊖︀⊖︀ VERY LOW | |  |
| Nausea | RR 1.62 (0.51 to 5.12) ⇩ | | 39  (1 RCT) | | (44) | | ⊖︀⊕⊖︀⊖︀ VERY LOW | |  |
| Stomach pain | RR 3.86 (0.17 to 89.03) ⇧ | | 39  (1 RCT) | | (44) | | ⊖︀⊕⊖︀⊖︀ VERY LOW | |  |
| Vomiting | RR 1.29 (0.38 to 4.44) ⇧ | | 39  (1 RCT) | | (44) | | ⊖︀⊕⊖︀⊖︀ VERY LOW | |  |

**S13. Forest plots**

- 1. **Systolic blood pressure (mmHg)**

**
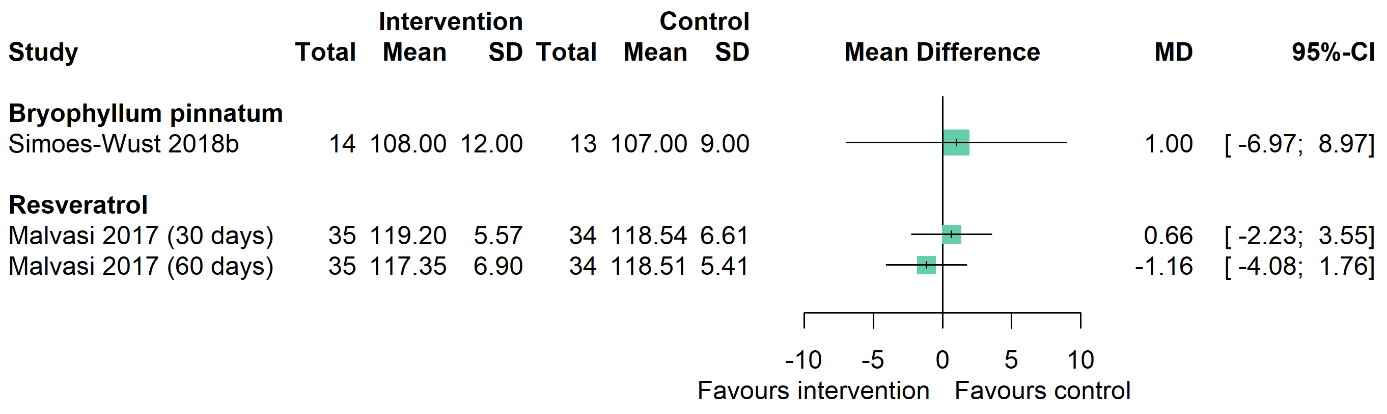
**

- 1. **Diastolic blood pressure (mmHg)**

**
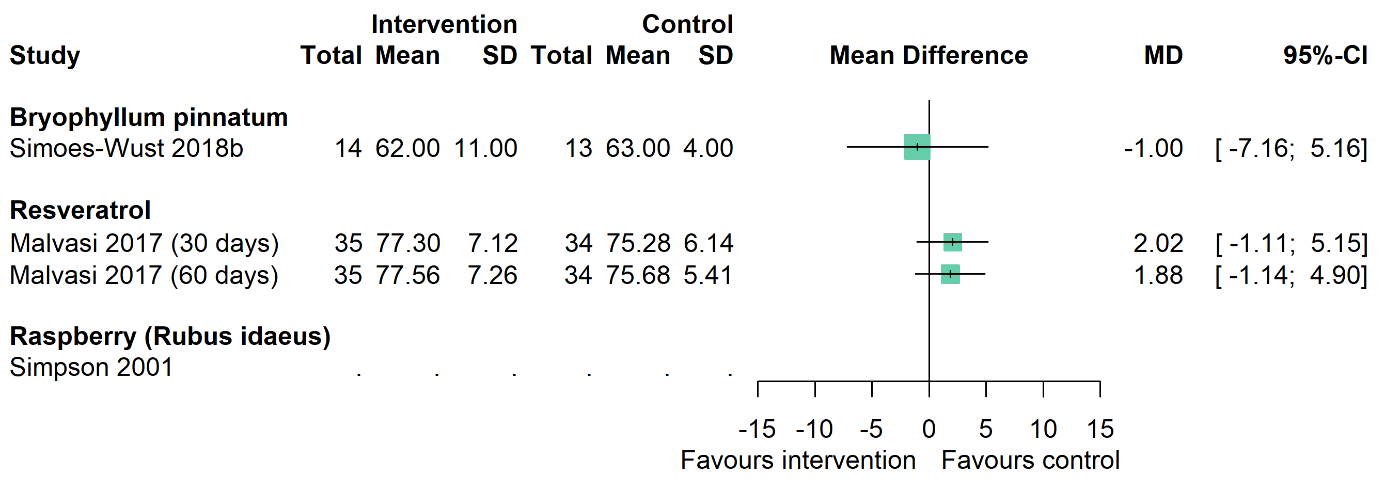
**

- 1. **Time needed to control blood pressure (min)**

**
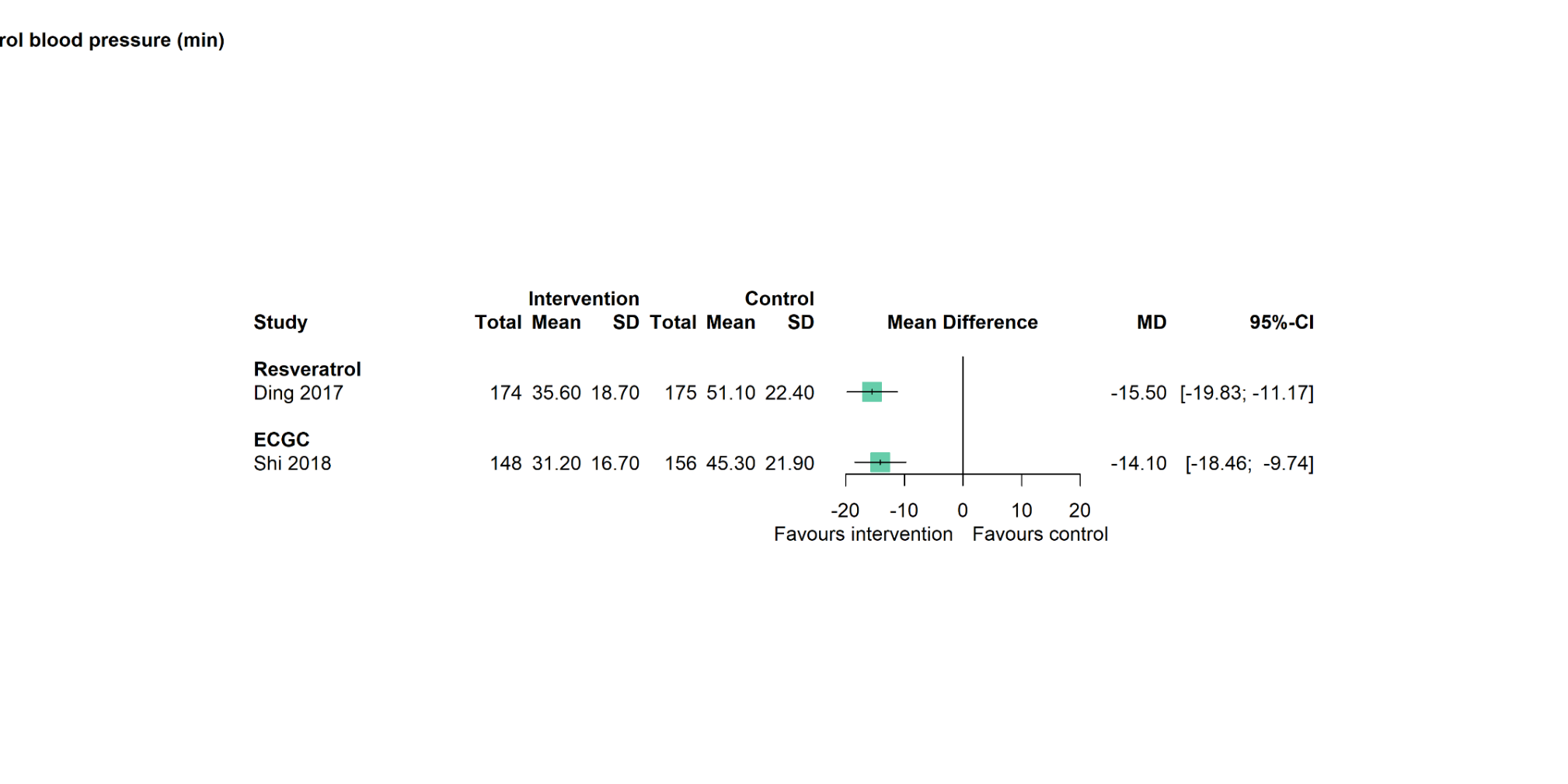
**

- 1. **Time to next hypertensive crisis (hr)**

**
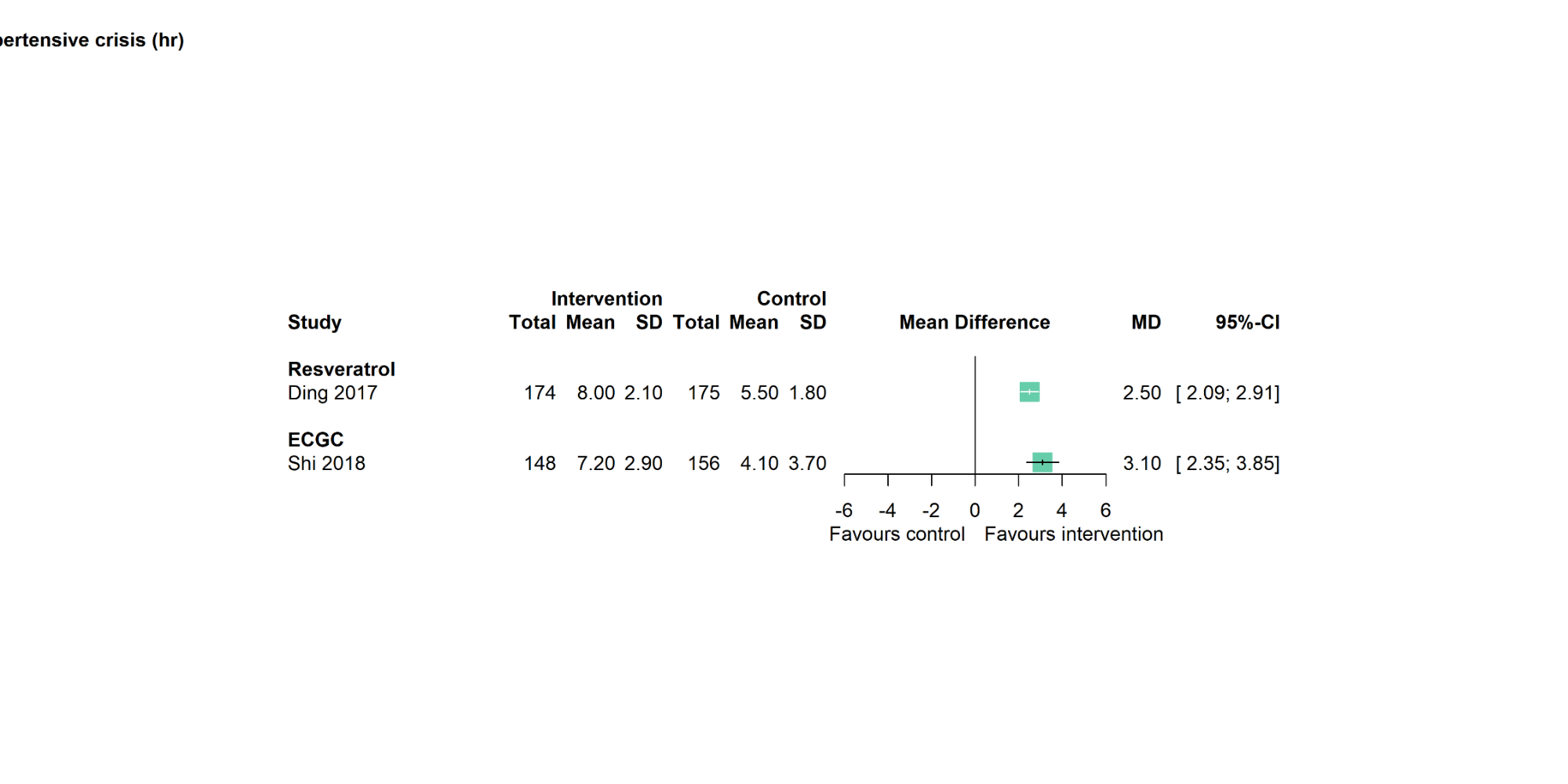
**

- 1. **Preeclampsia**

**
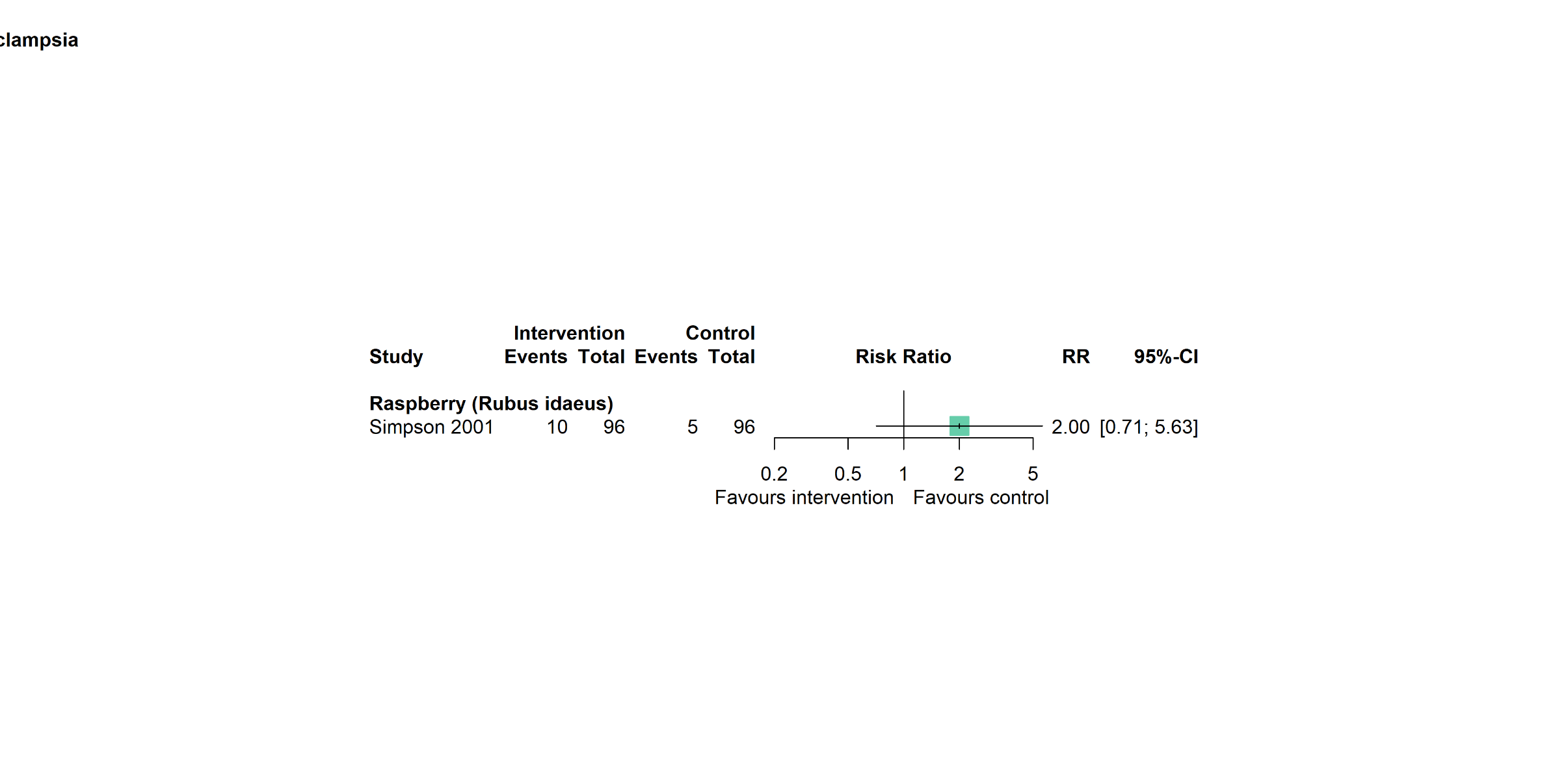
**

- 1. **Hypertensive disorders of pregnancy**

**
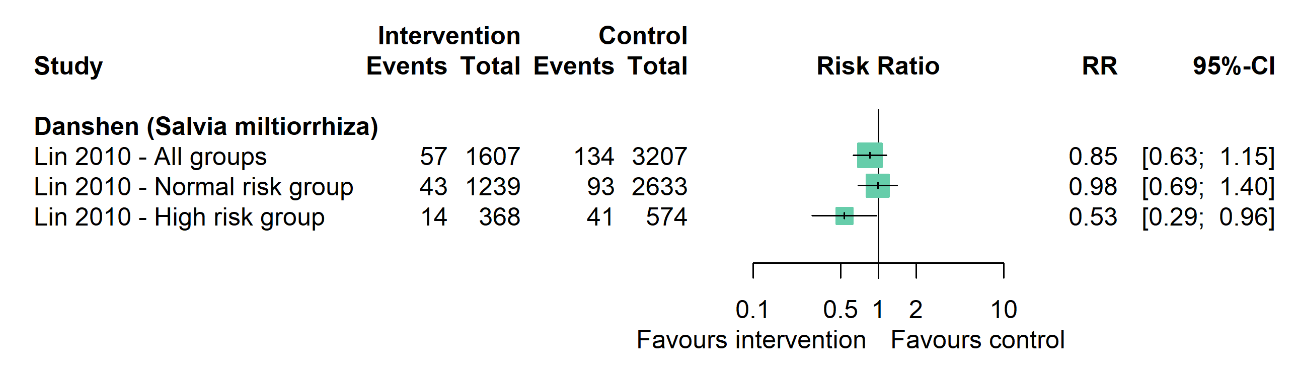
**

- 1. **Elevated transaminases (ALT/AST)**

**
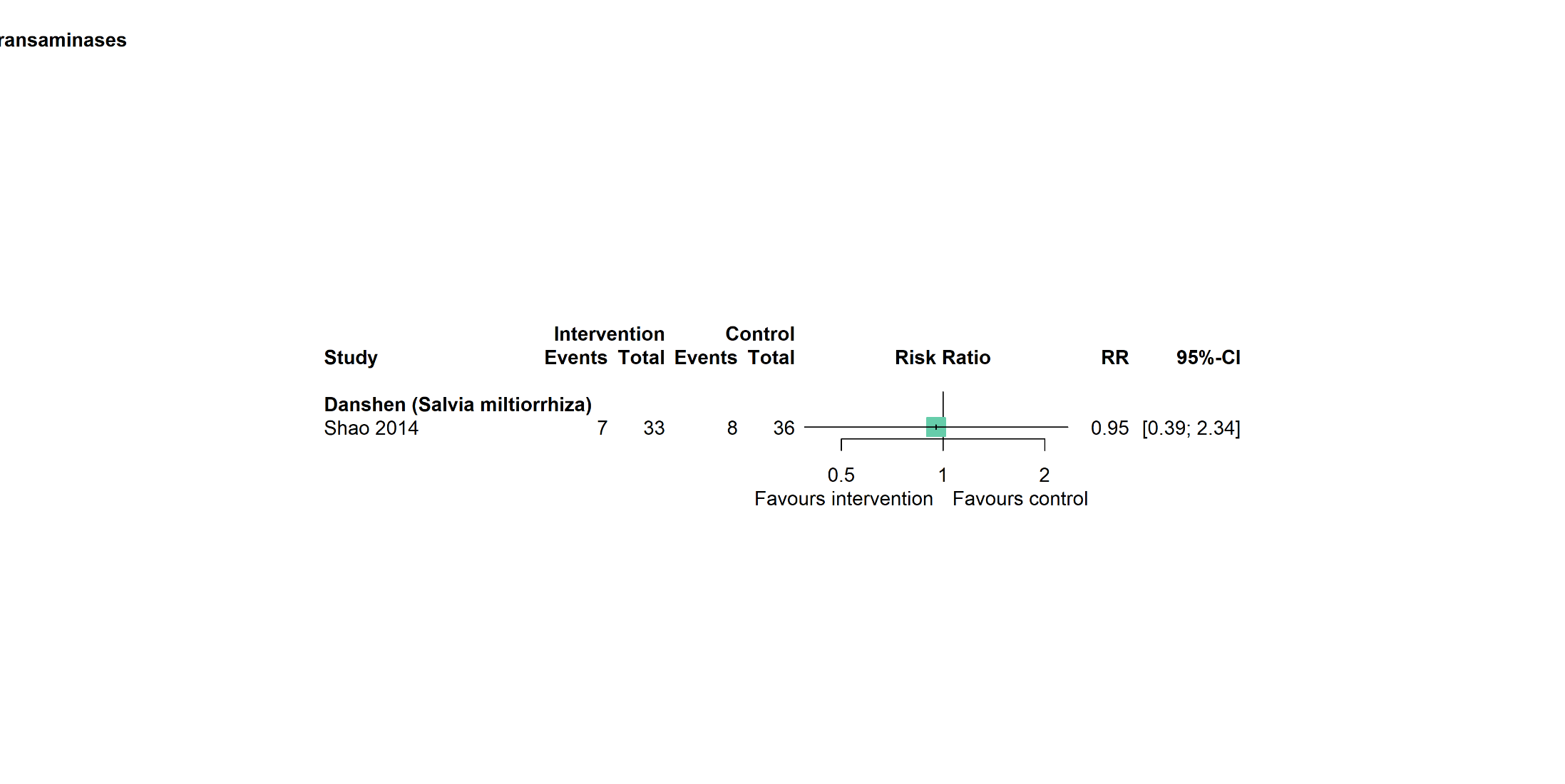
**

- 1. **Placental abruption**

**
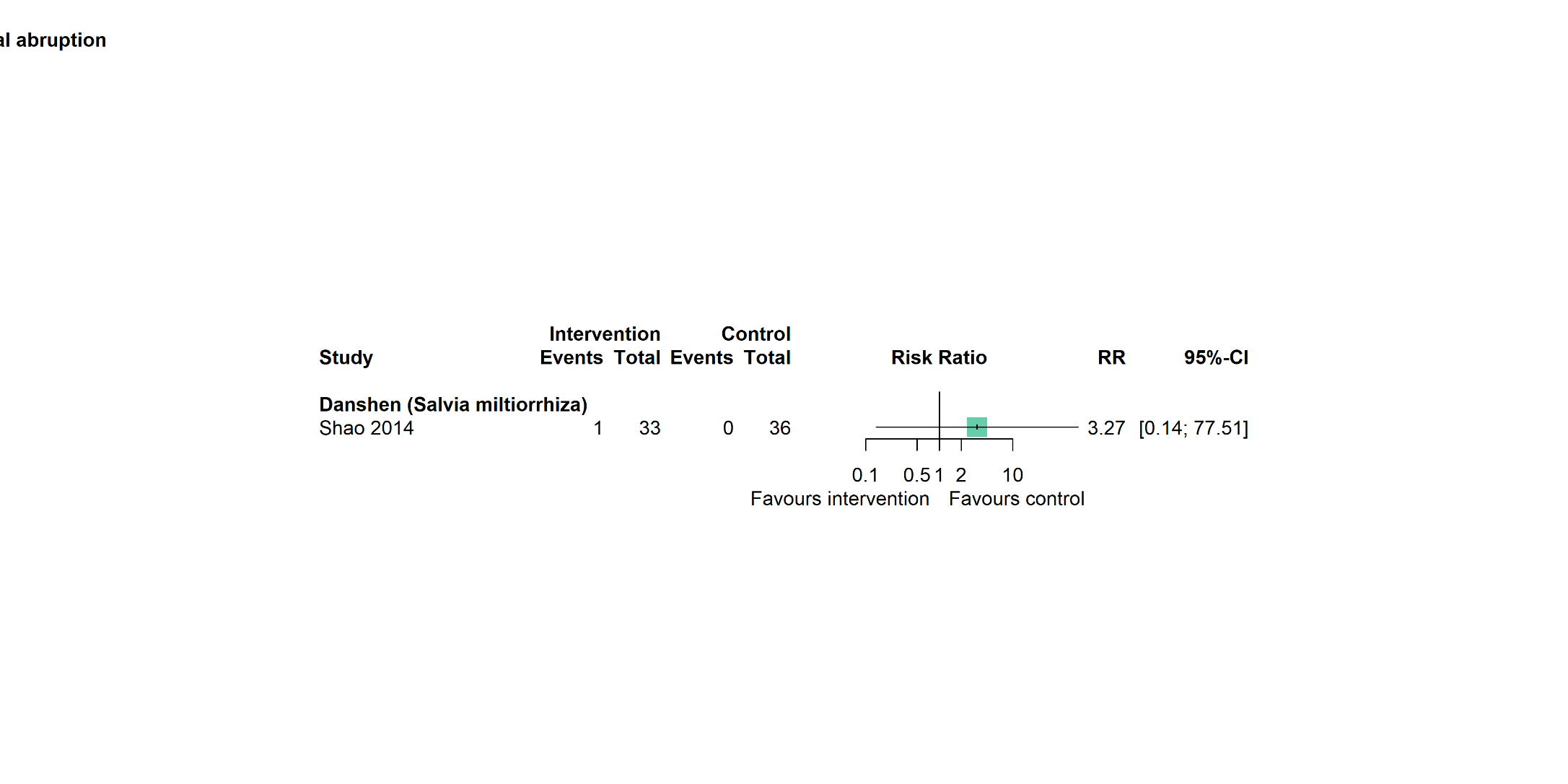
**

- 1. **Postpartum hemorrhage**

**
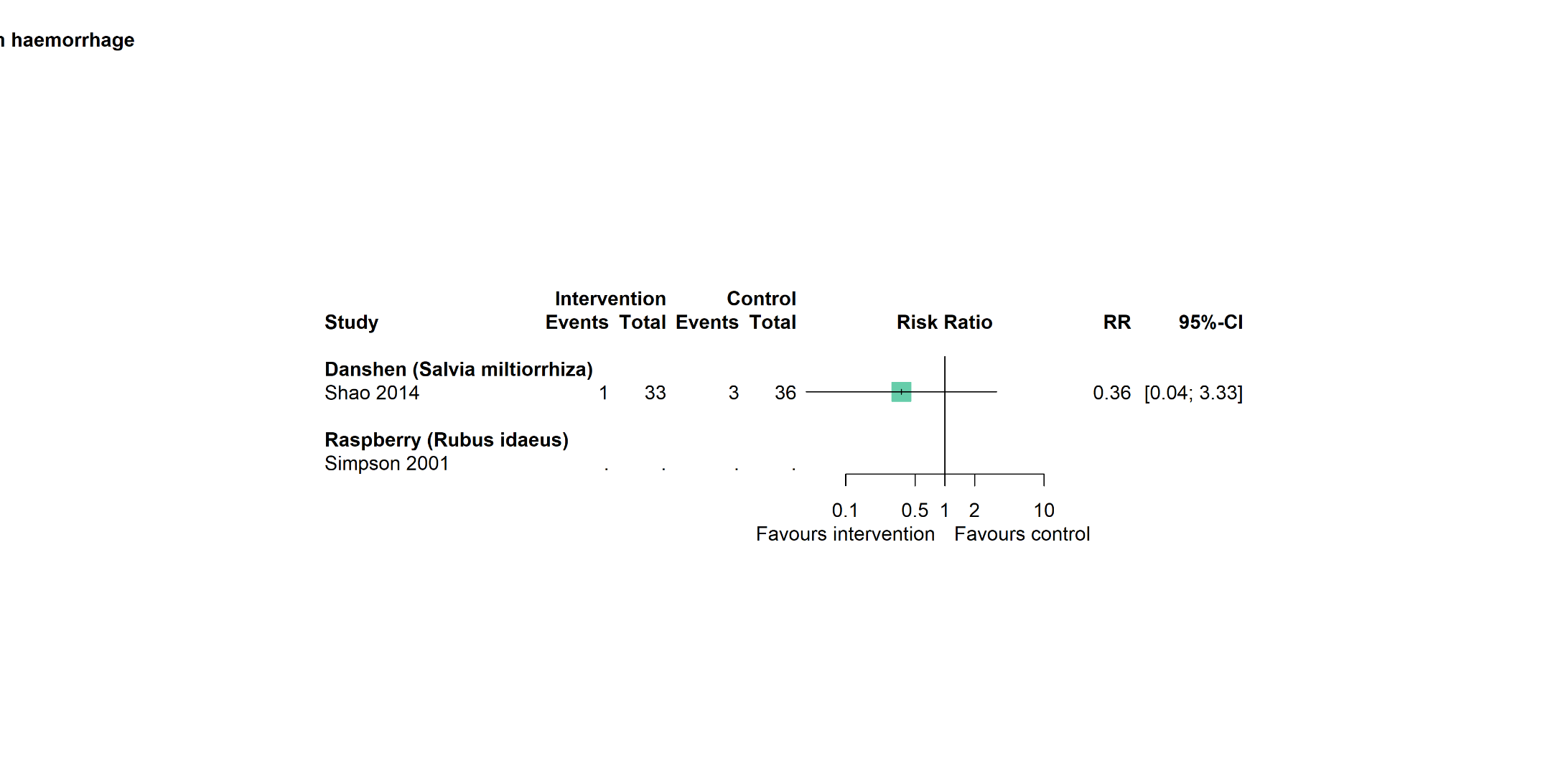
**

- 1. **Amount of blood loss (mL)**

No forest plot for this outcome. The only data is for **Raspberry (Rubus idaeus)** [Simpson 2001], for which only the effect estimate was available (MD -11.70 [-141.49 to 118.09], 148 women).

- 1. **Apgar score [5-min] (points)**

**
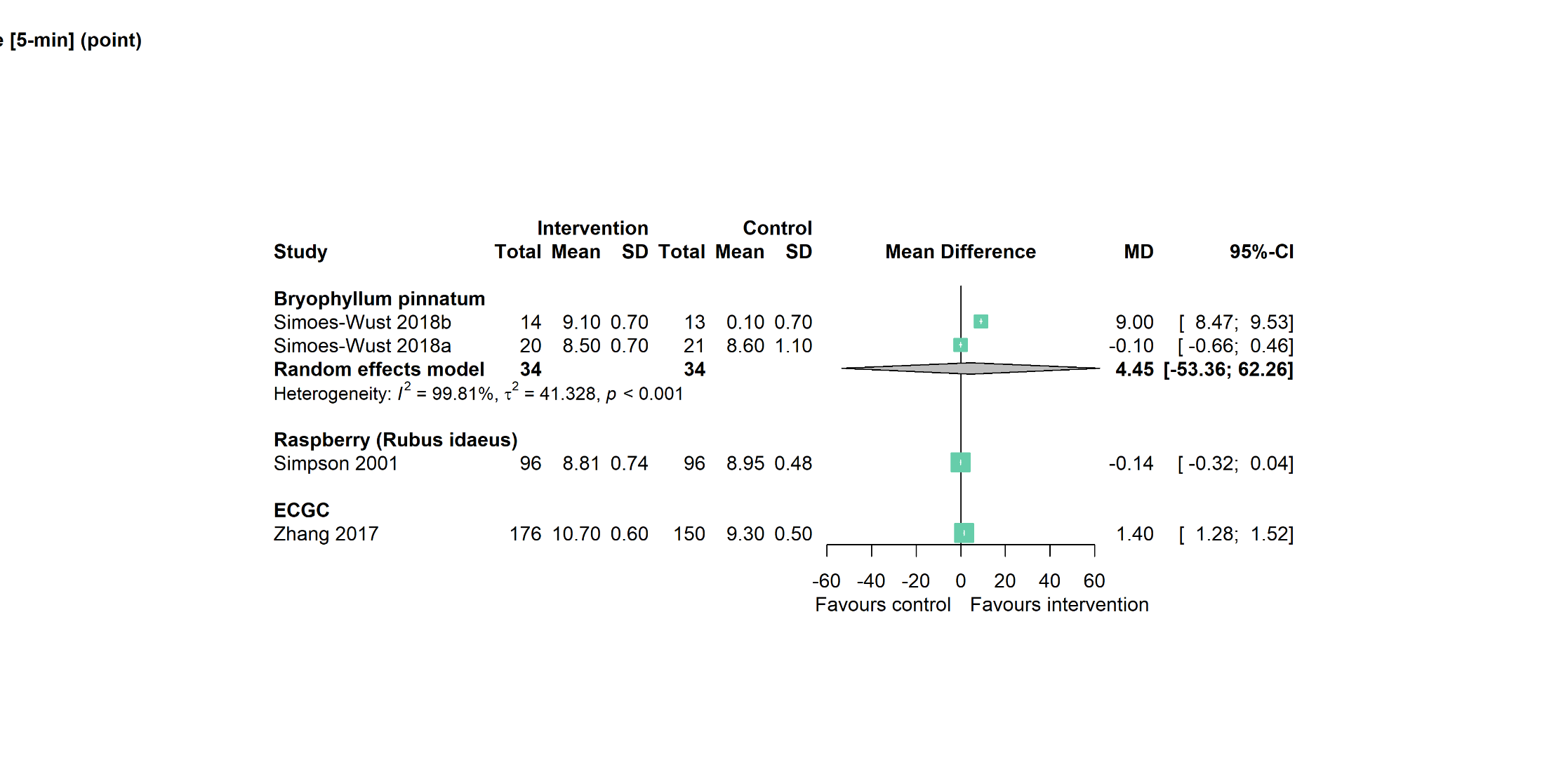
**

- 1. **High Apgar scores**

**
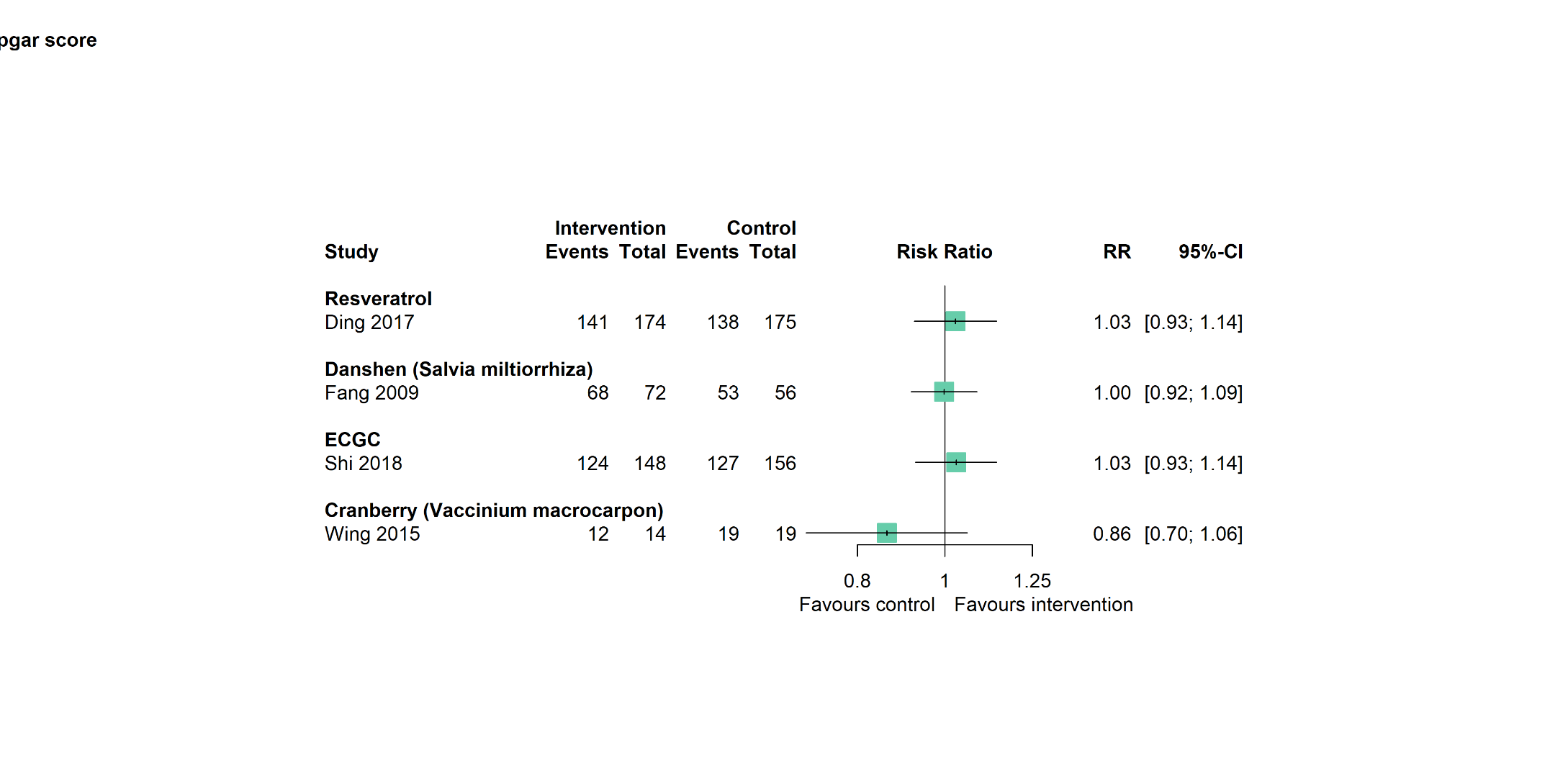
**

- 1. **Birth weight (g)**


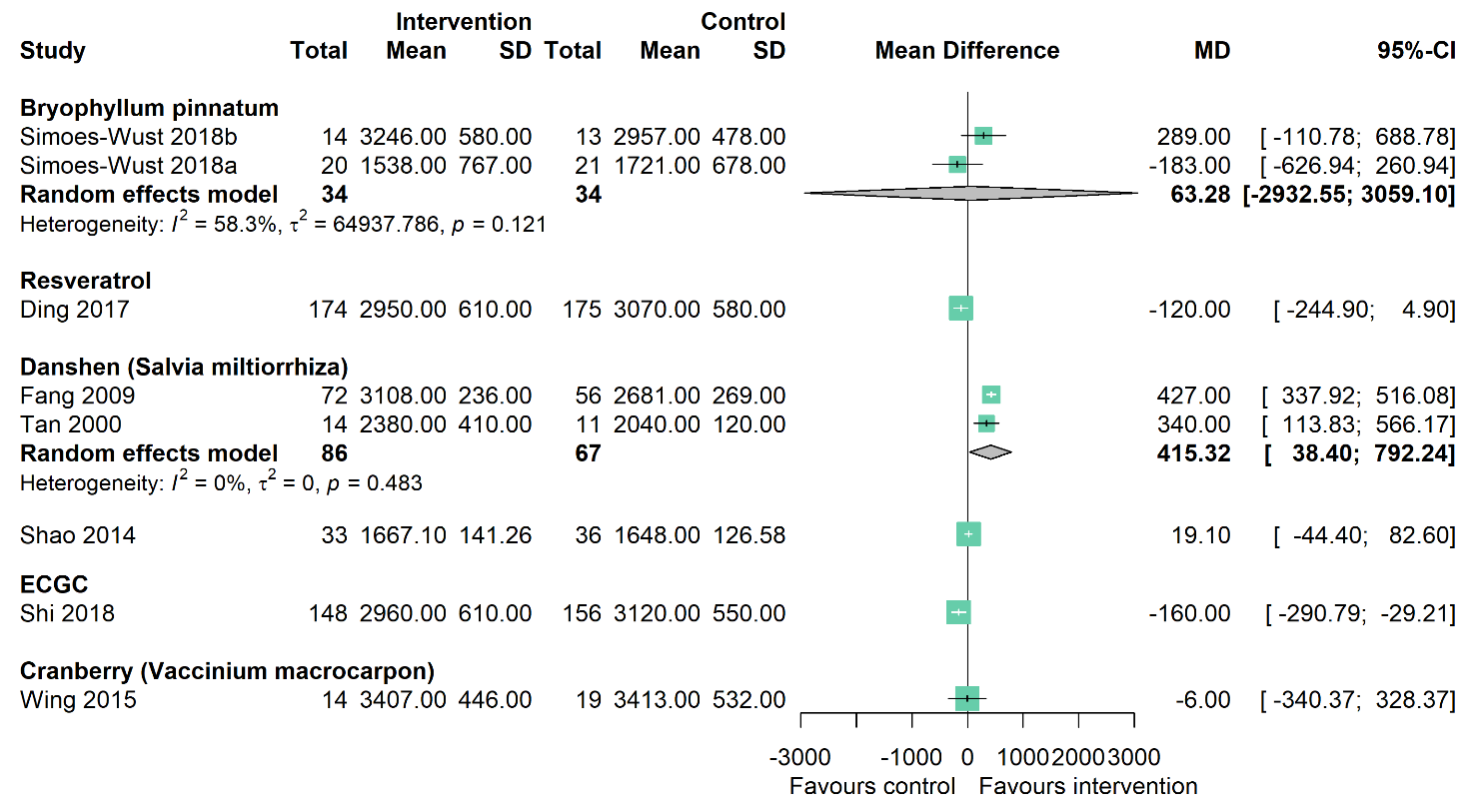


Data for **Raspberry (Rubus idaeus)** [Simpson 2001] was not included on forest plot as only the effect estimate was available (MD = -44.27 [-161.47 to 72.93], 192 women).

- 1. **Low birth weight (birth weight <2,500g)**

**
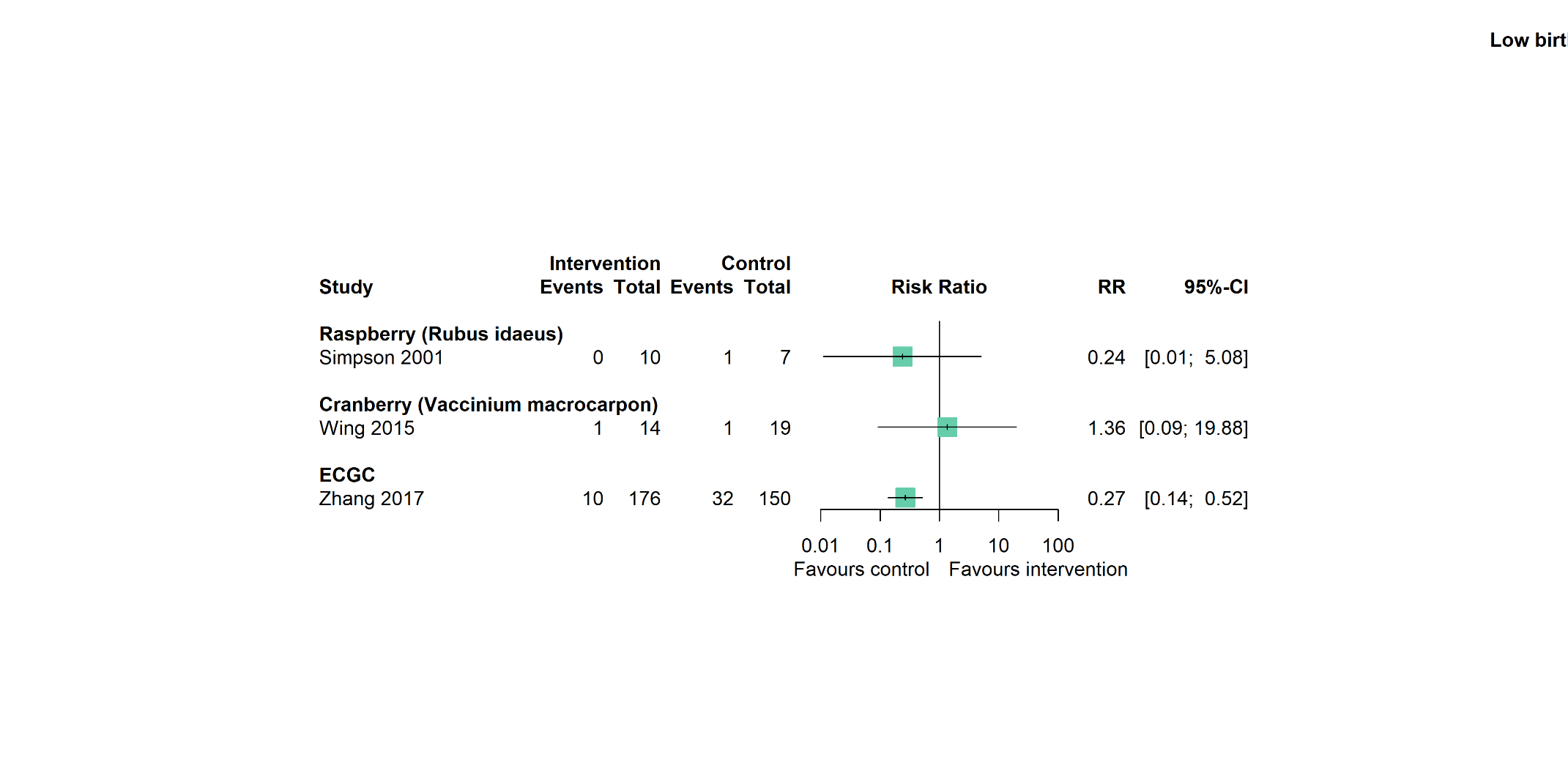
**

- 1. **Macrosomia**

**
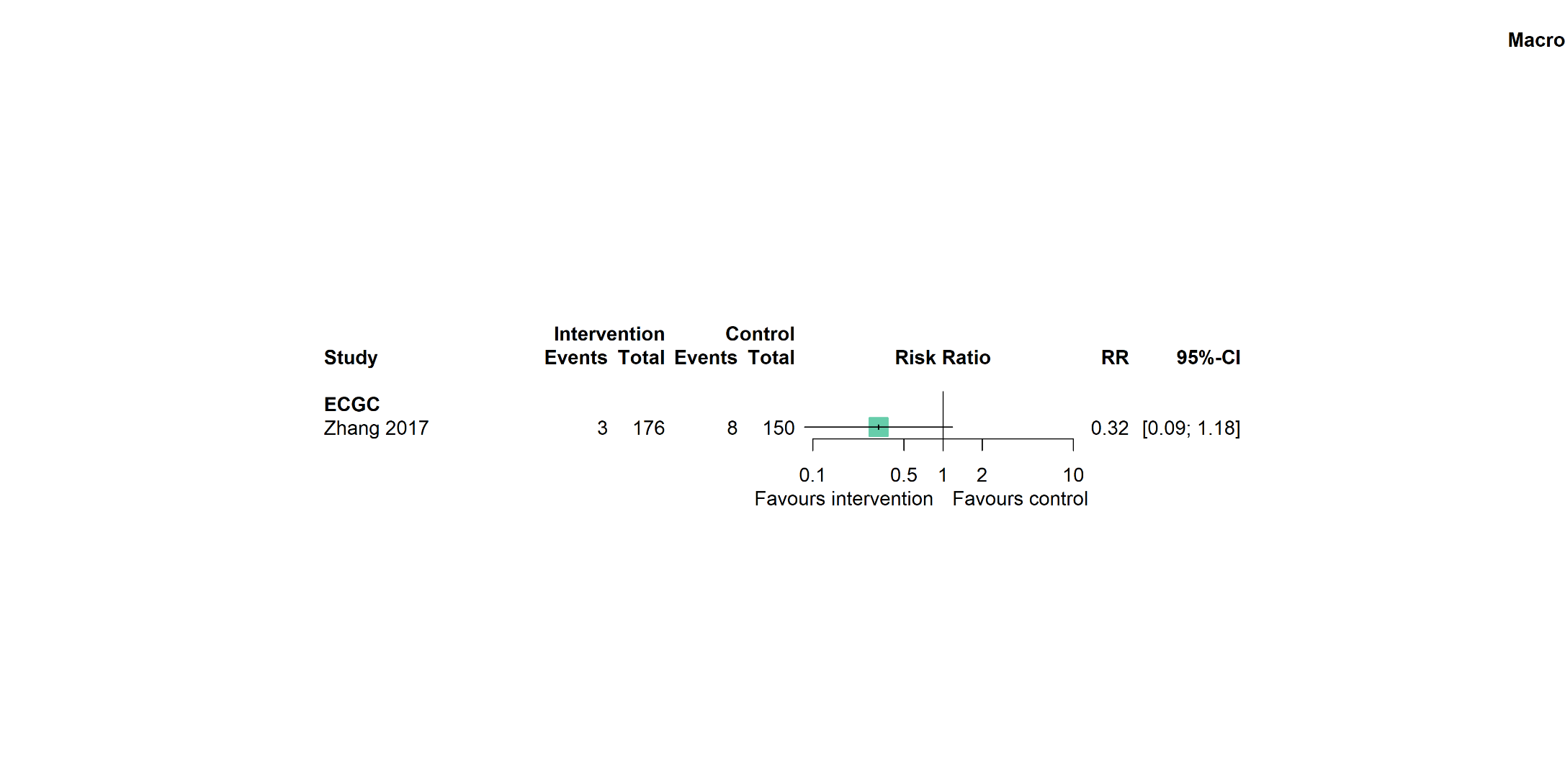
**

- 1. **Fetal distress**

**
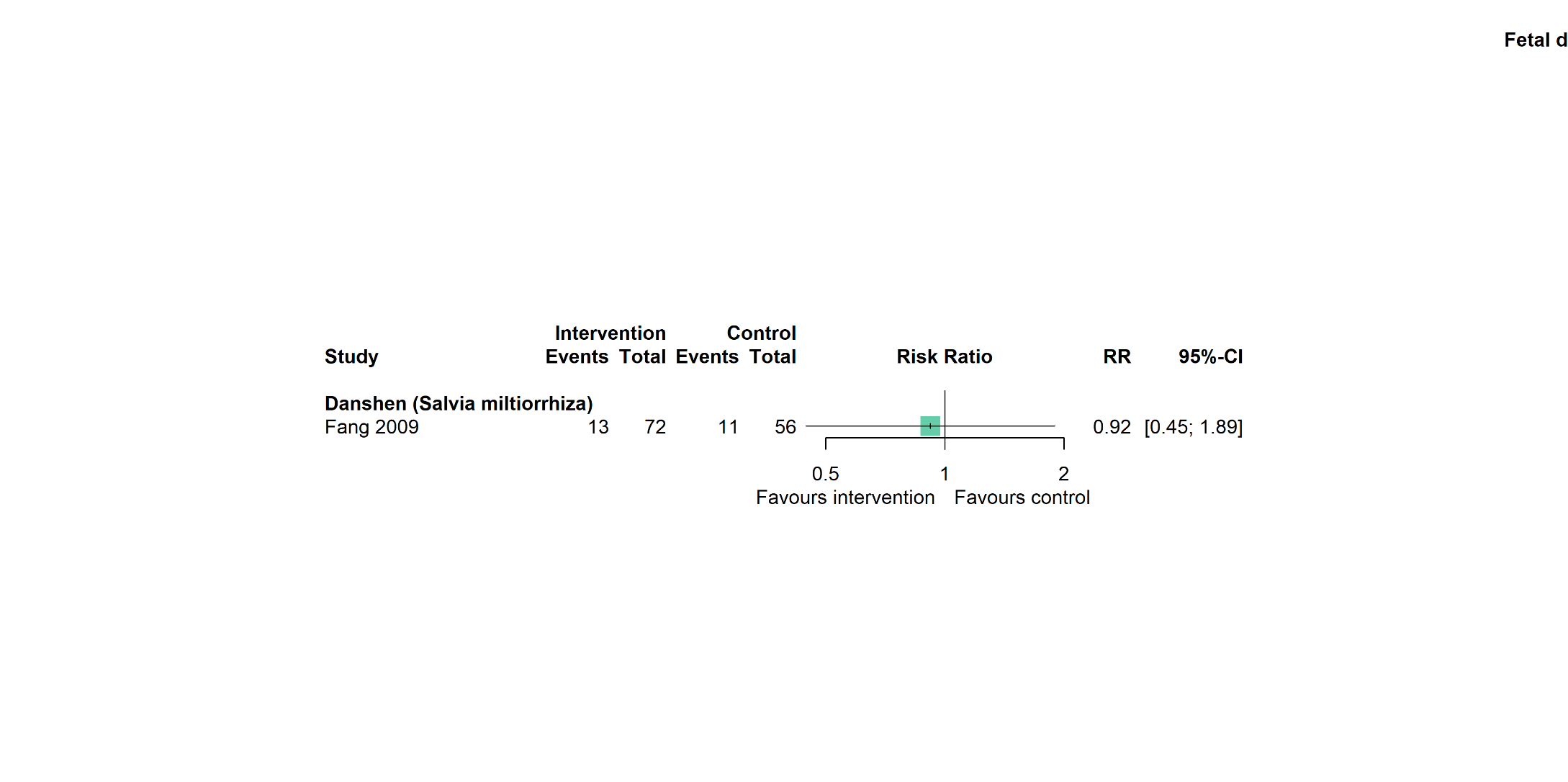
**

- 1. **Gestational age at birth (weeks)**

**
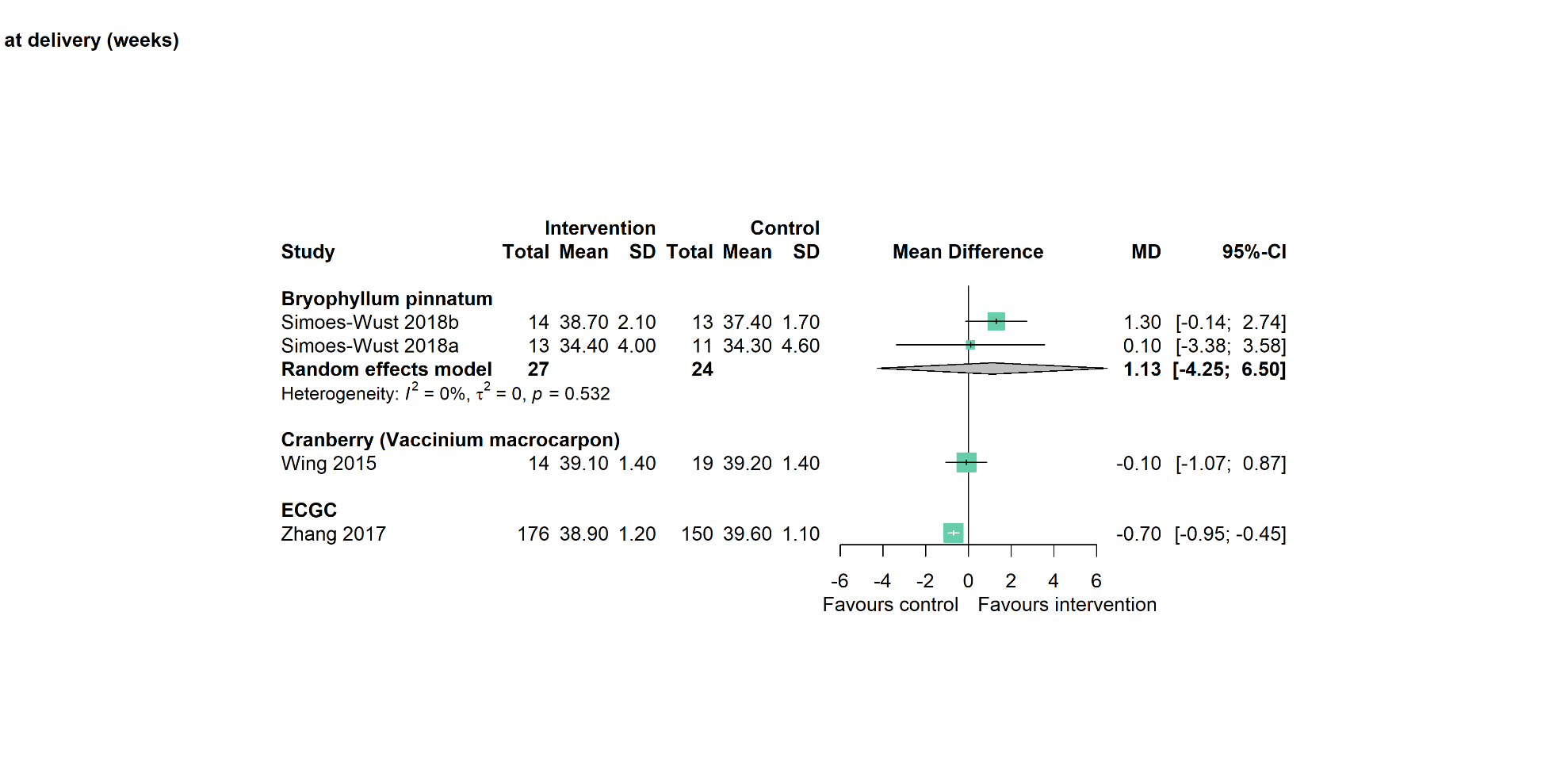
**

Data for **Raspberry (Rubus idaeus)** [Simpson 2001] was not included on forest plot as only the effect estimate was available (MD = -0.11 [-0.44 to 0.22], 192 women).

- 1. **Preterm birth**

**
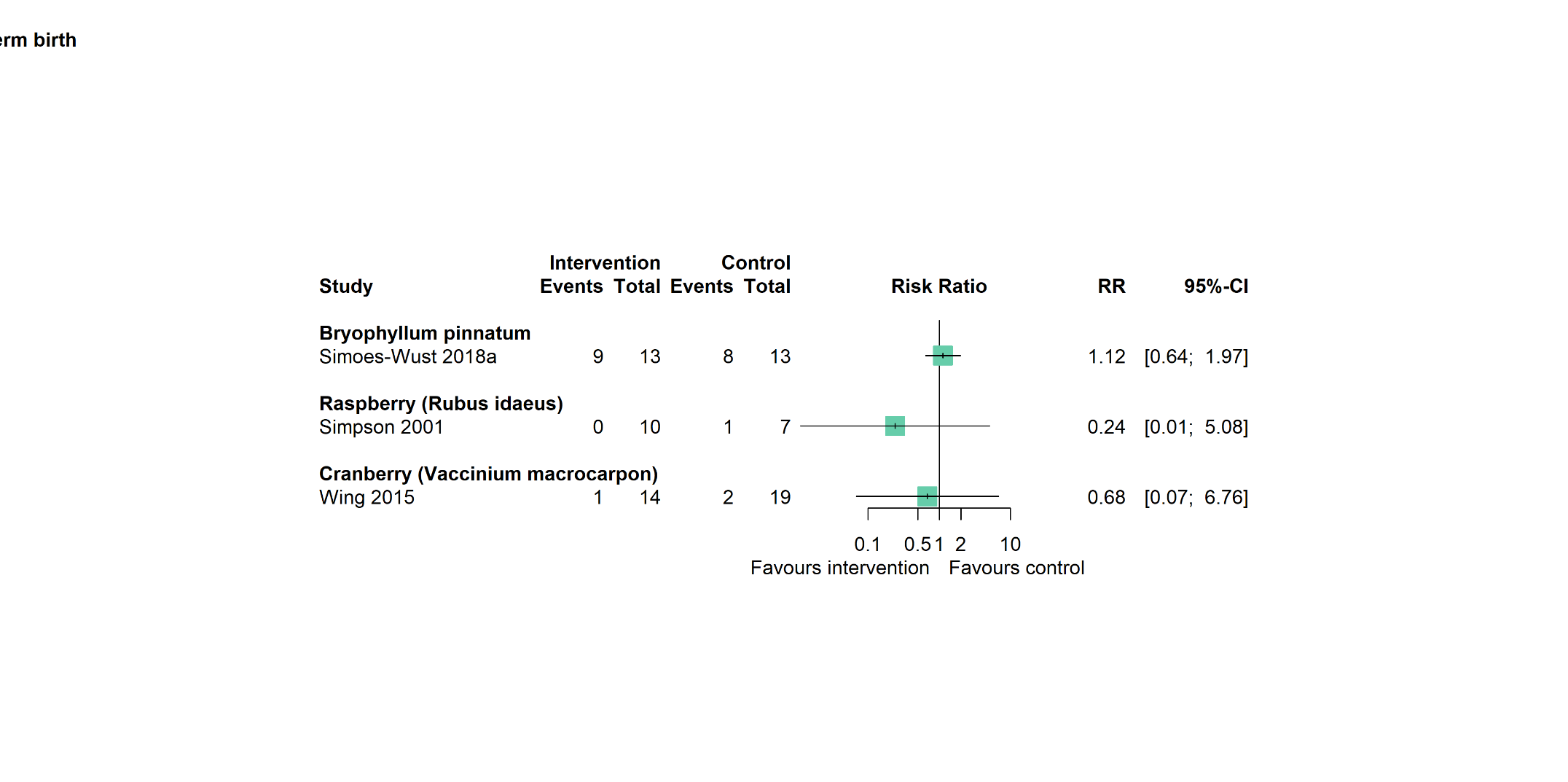
**

- 1. **Meconium staining of amniotic fluid**

**
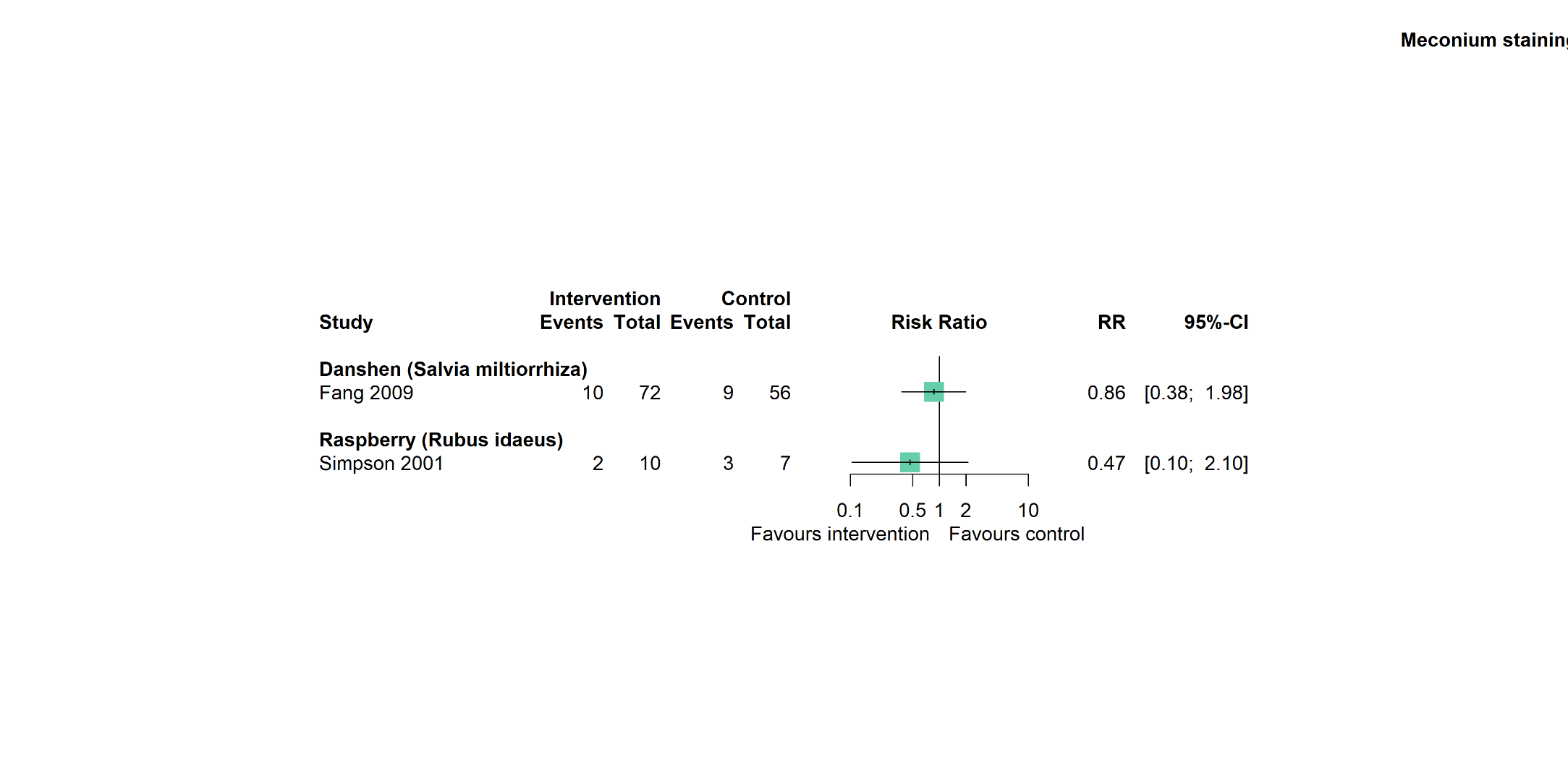
**

- 1. **Neonatal asphyxia**

**
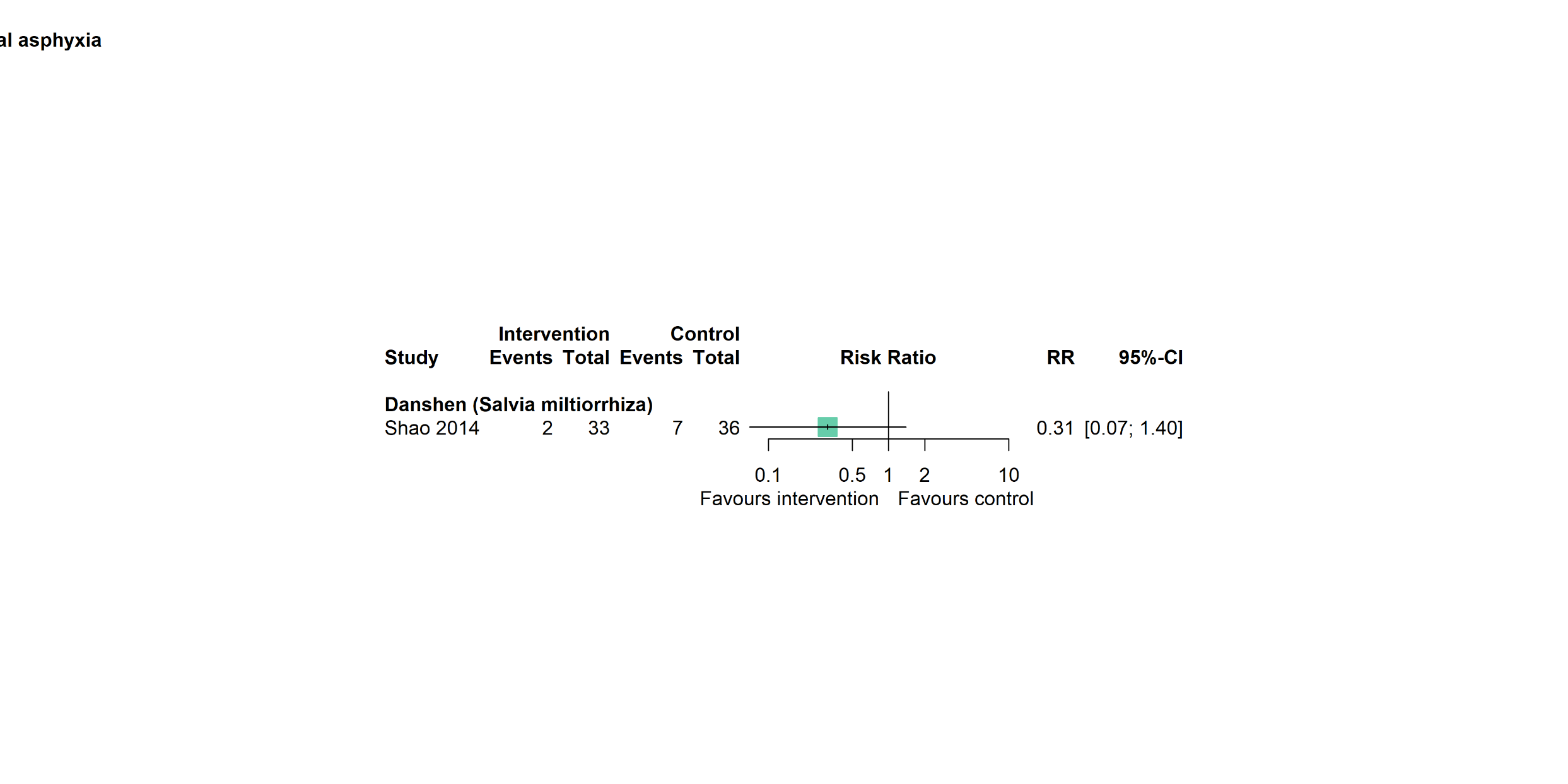
**

- 1. **Neonatal hypoglycemia**

**
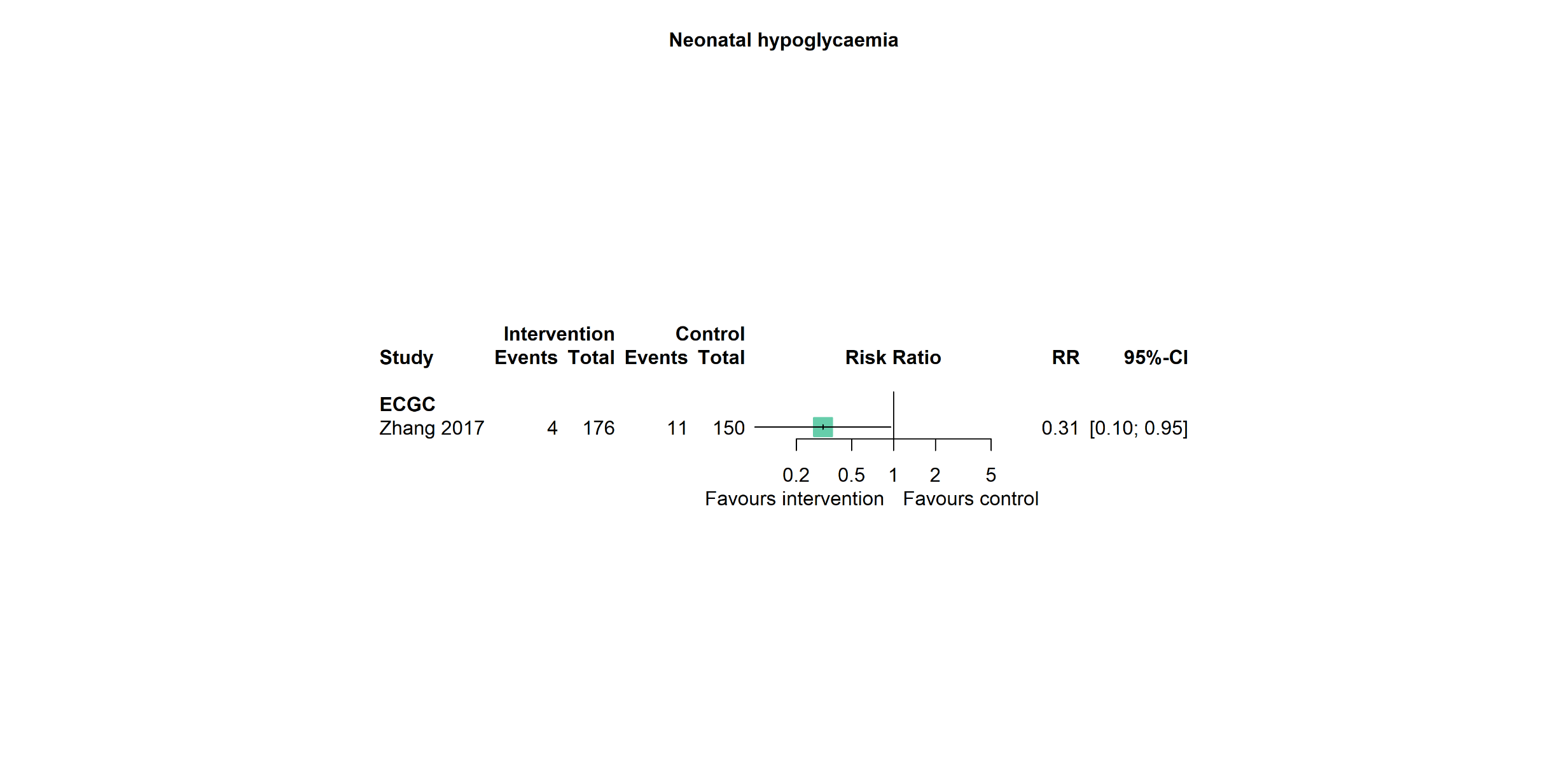
**

- 1. **NICU admission**

**
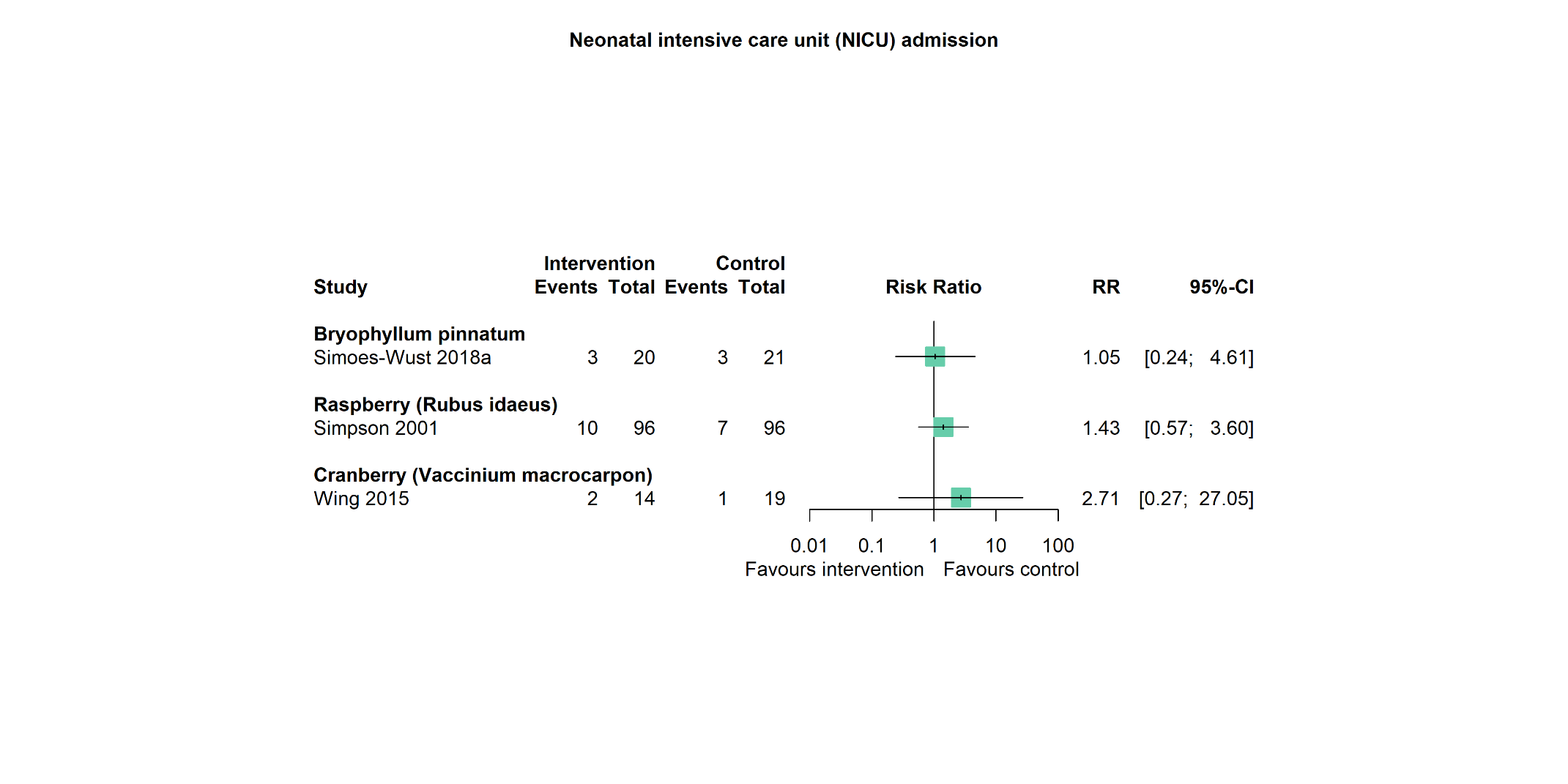
**

- 1. **Neonatal respiratory distress**

**
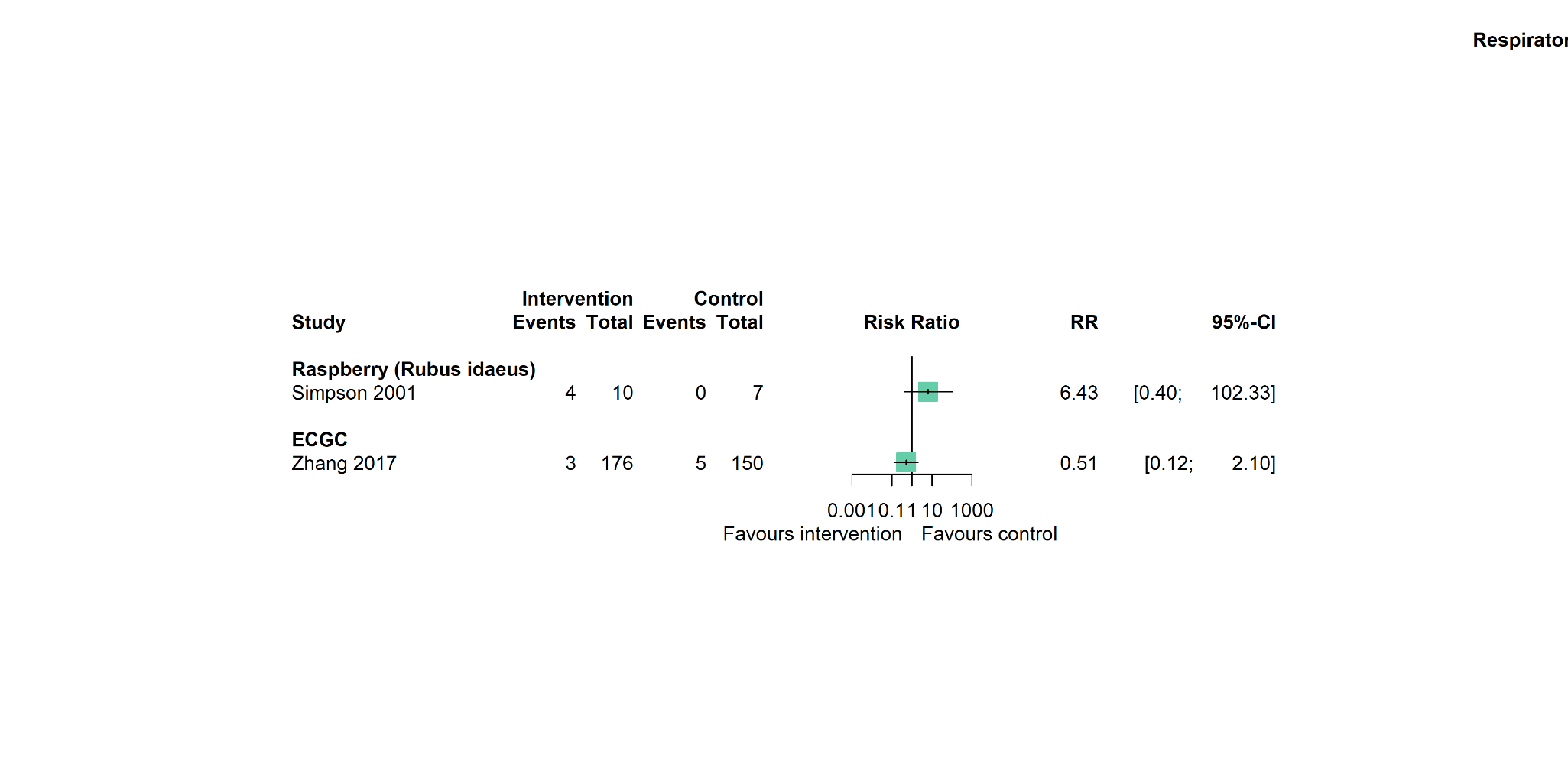
**

- 1. **Side effects – Bloating**

**
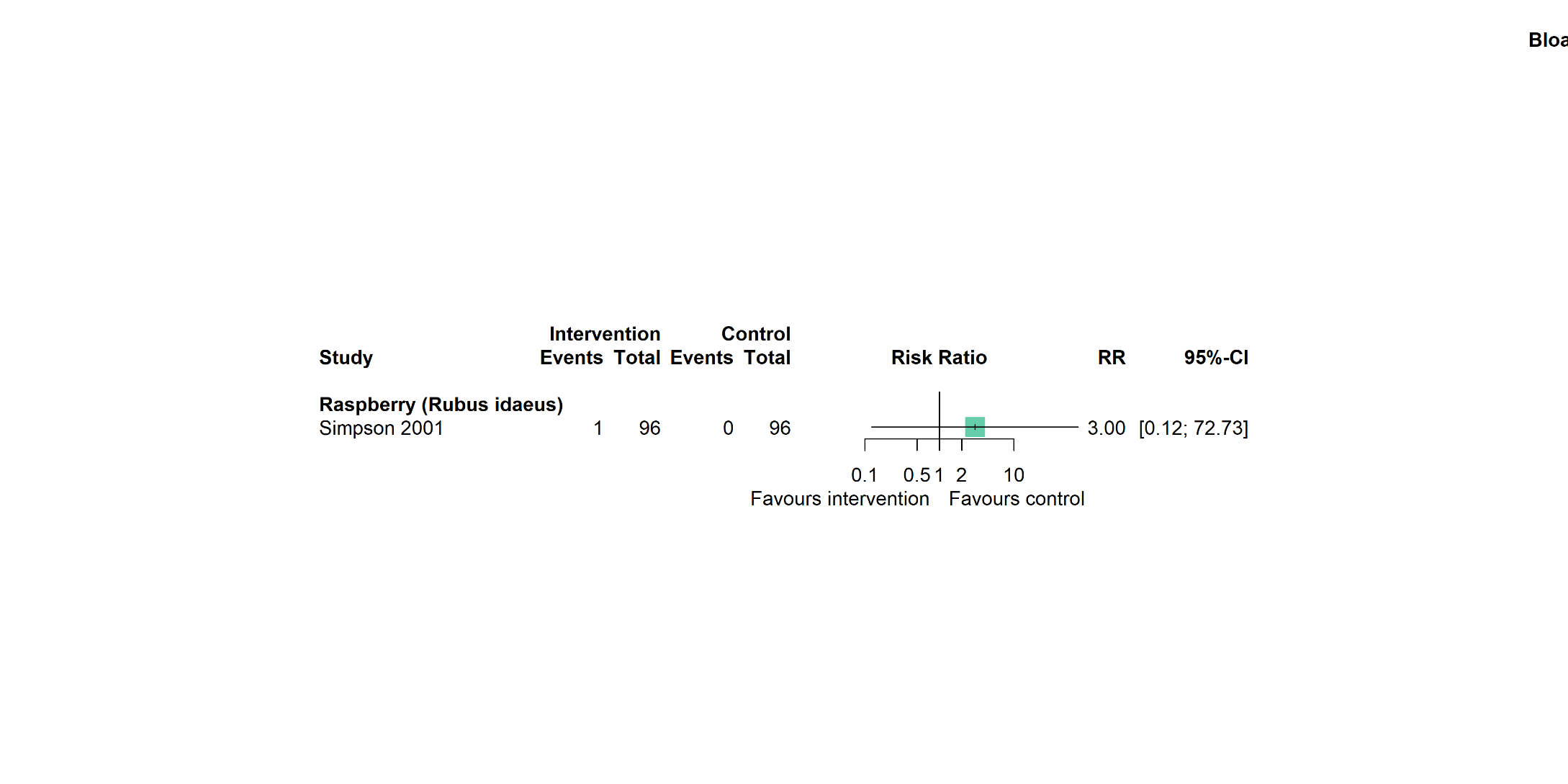
**

- 1. **Side effects – Chest pain**

**
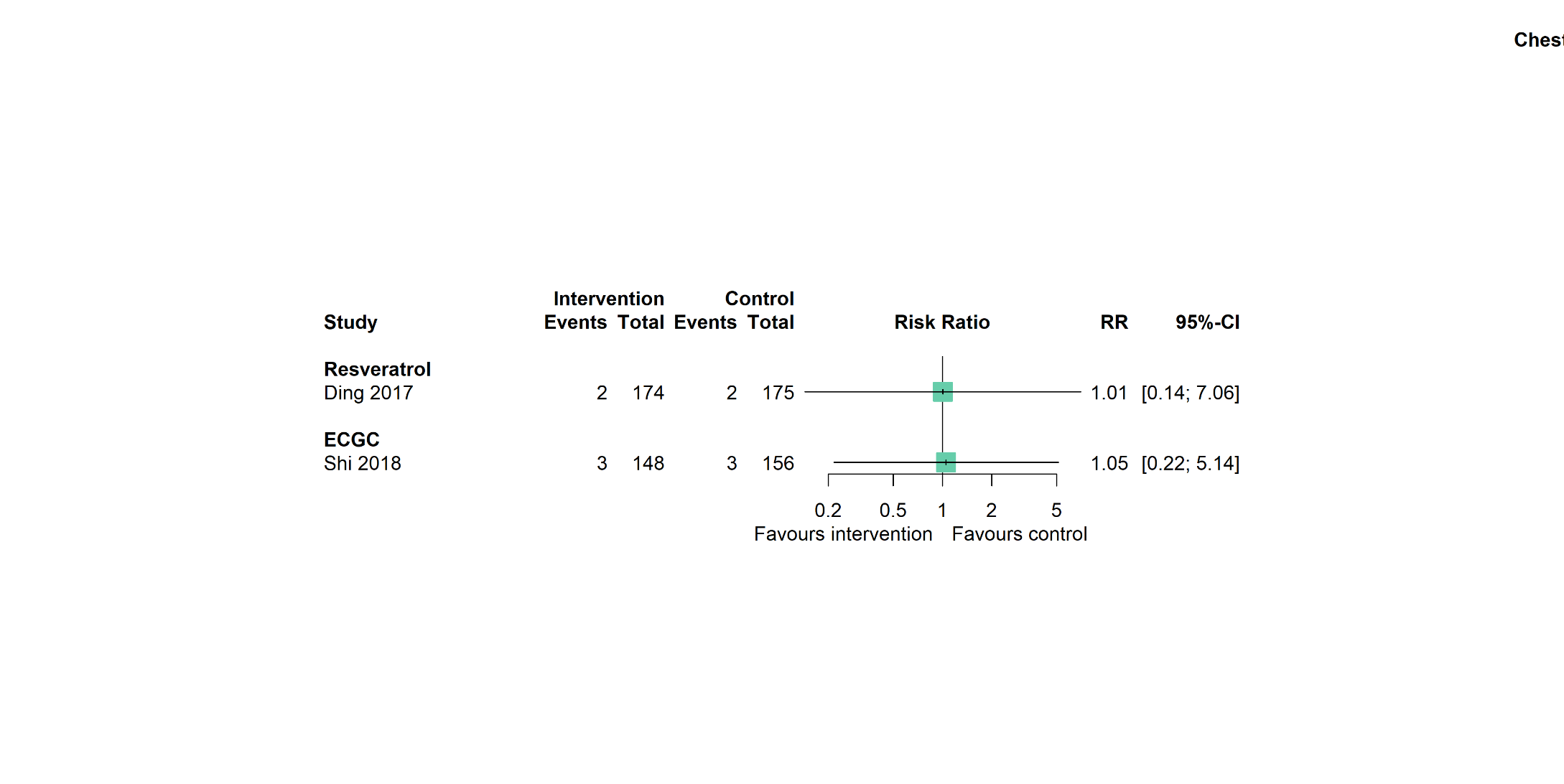
**

- 1. **Side effects – Constipation**

**
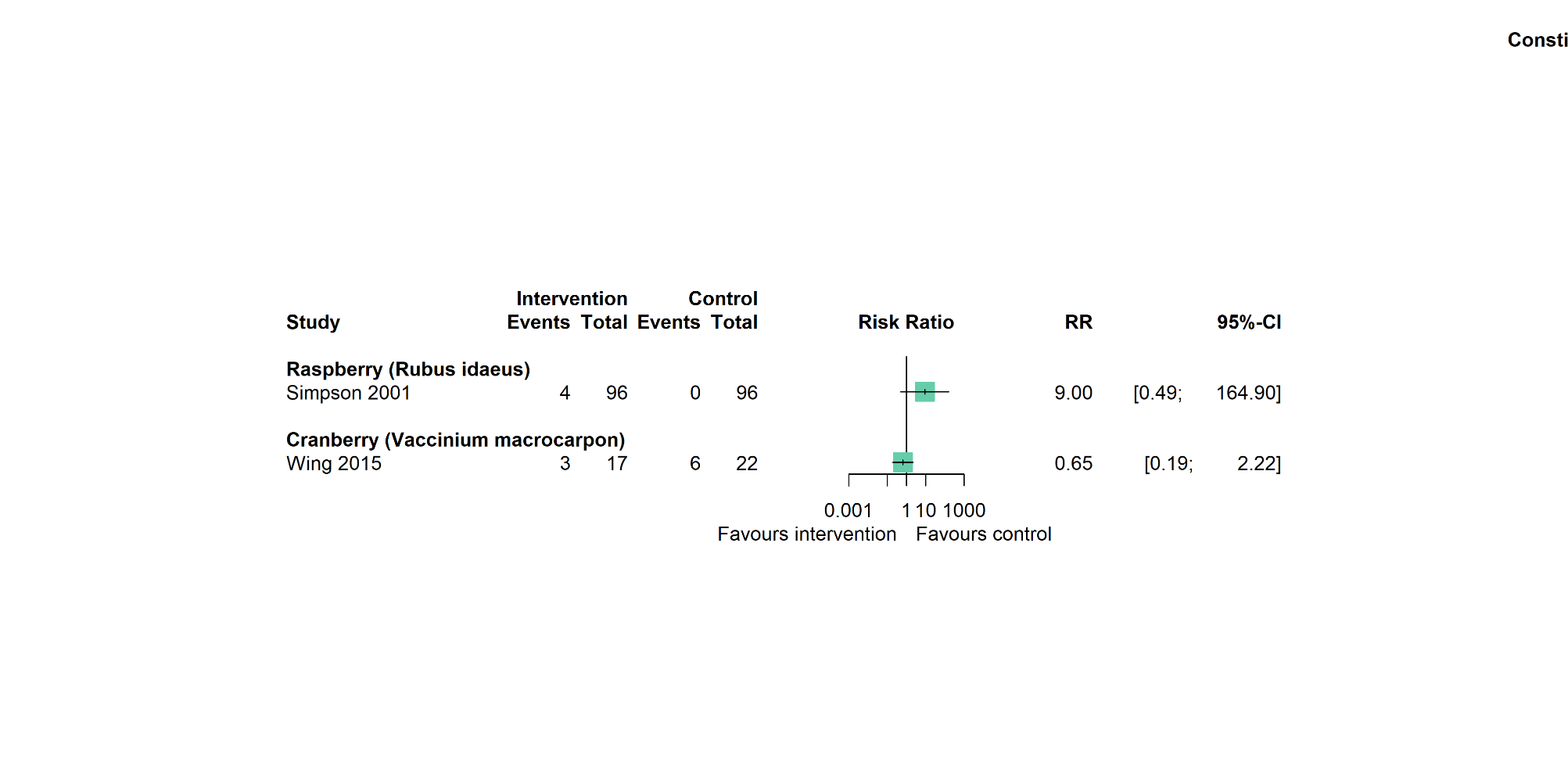
**

- 1. **Side effects – Diarrhea**

**
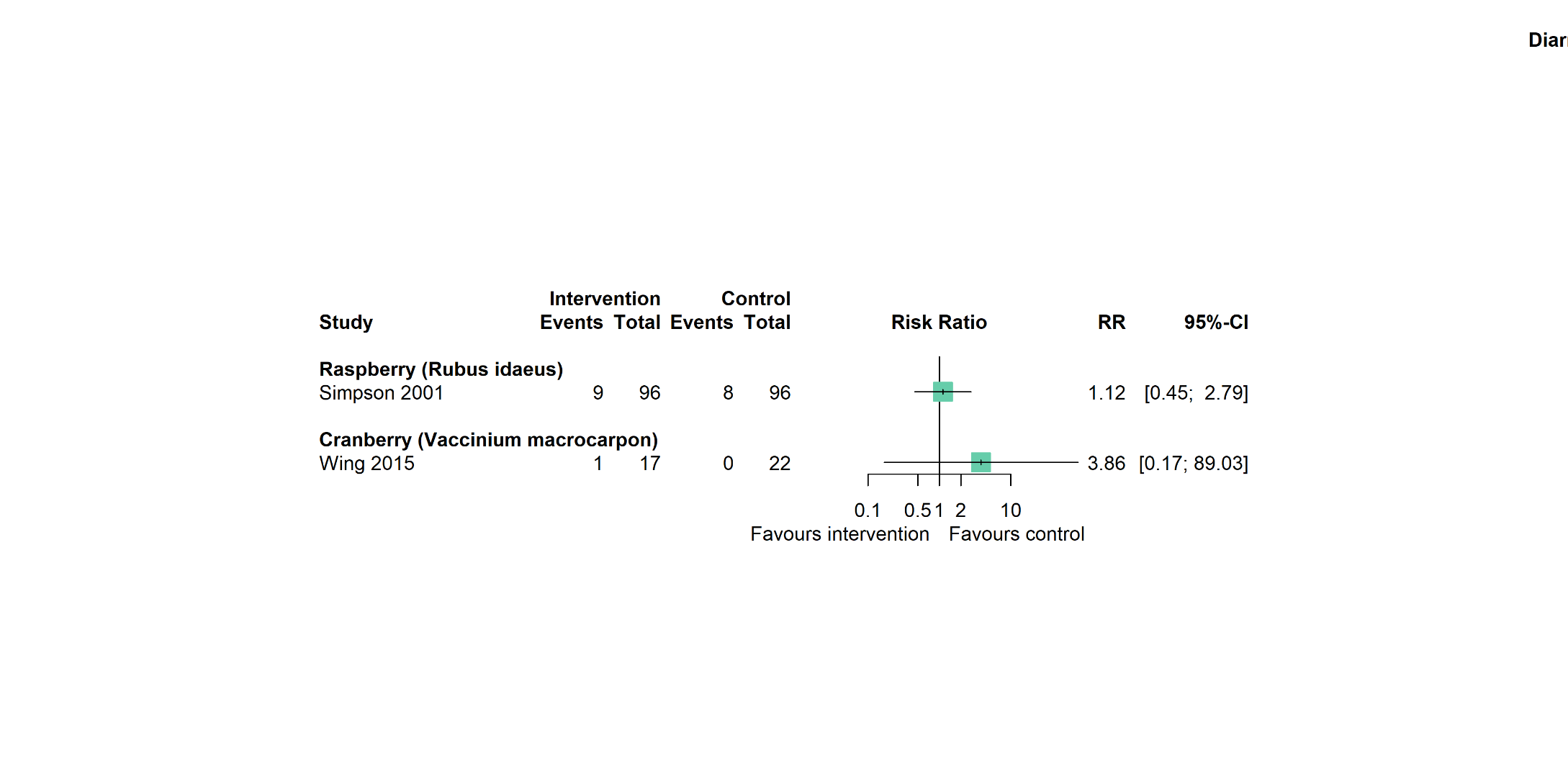
**

- 1. **Side effects – Dizziness**

**
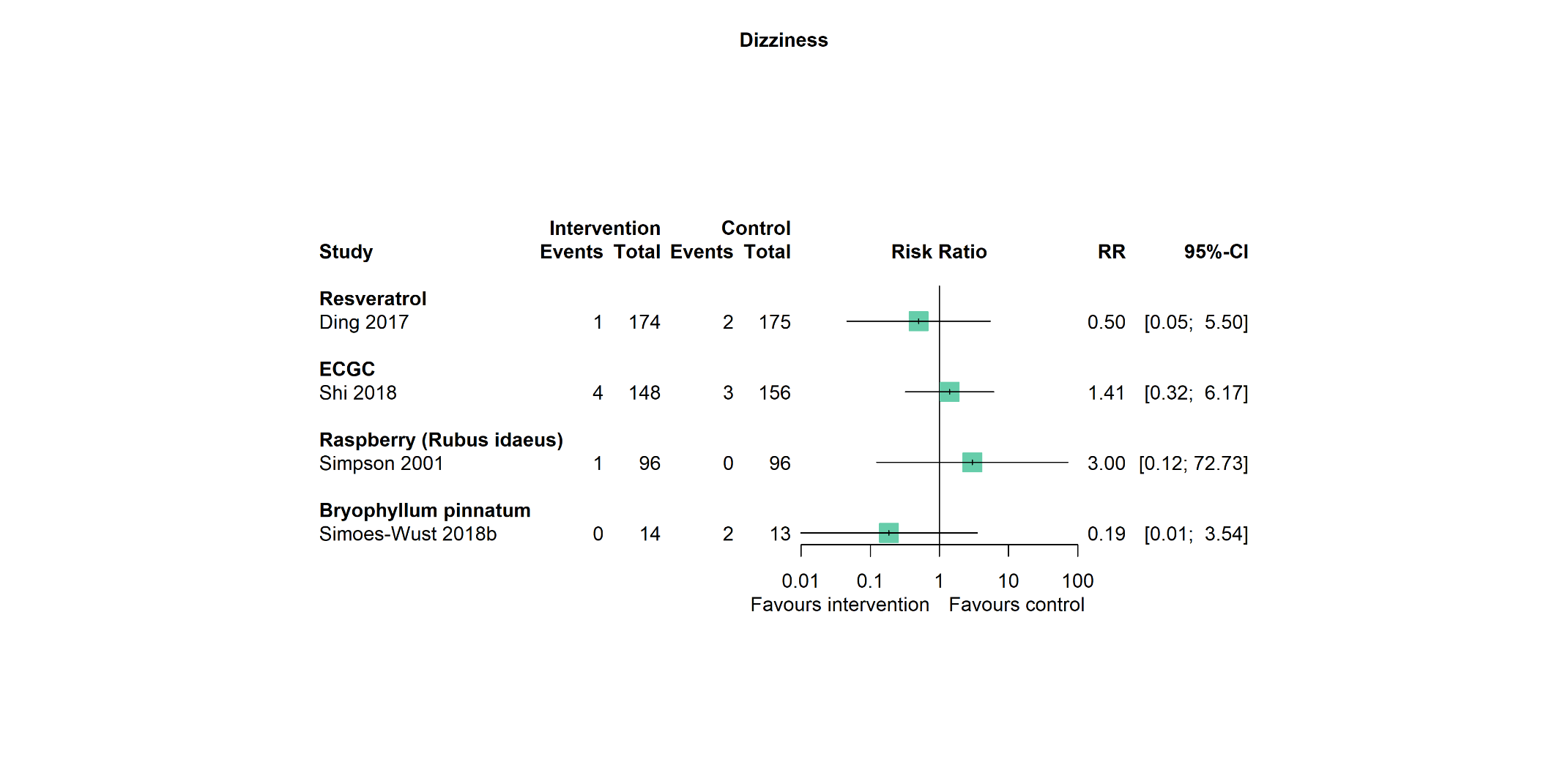
**

- 1. **Side effects – Headache**

**
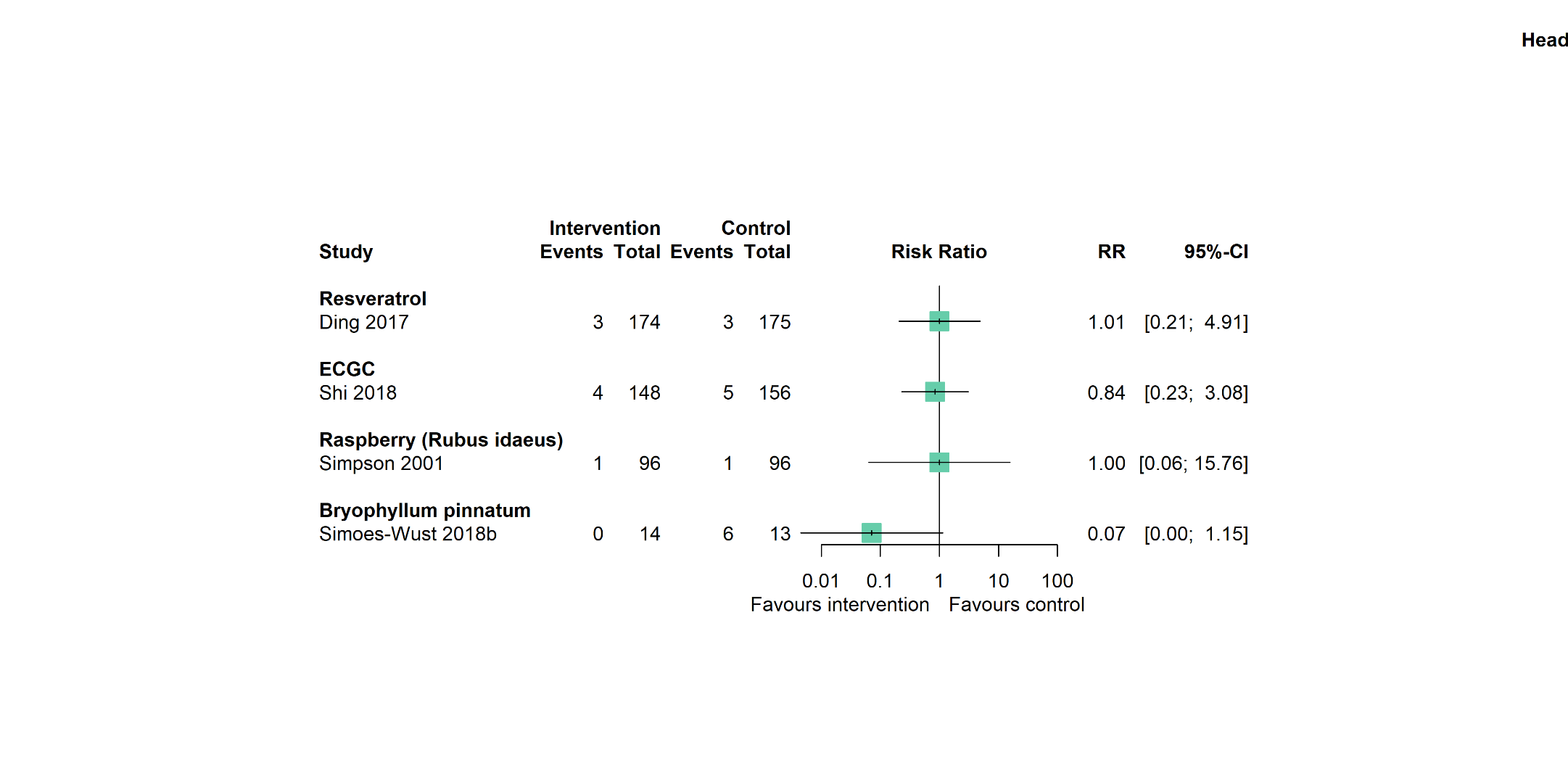
**

- 1. **Side effects – Heartburn**

**
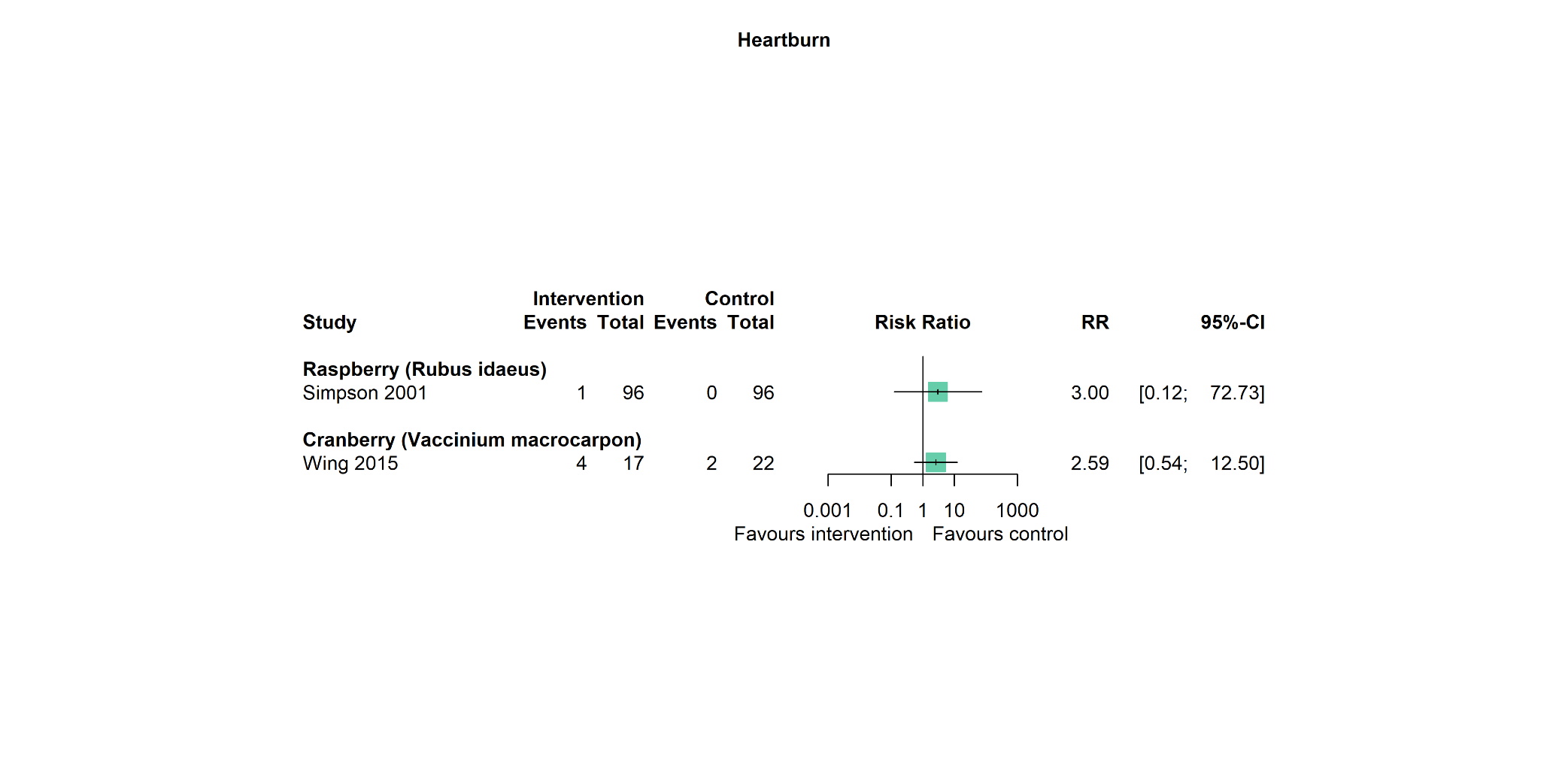
**

- 1. **Side effects – Loss of appetite**

**
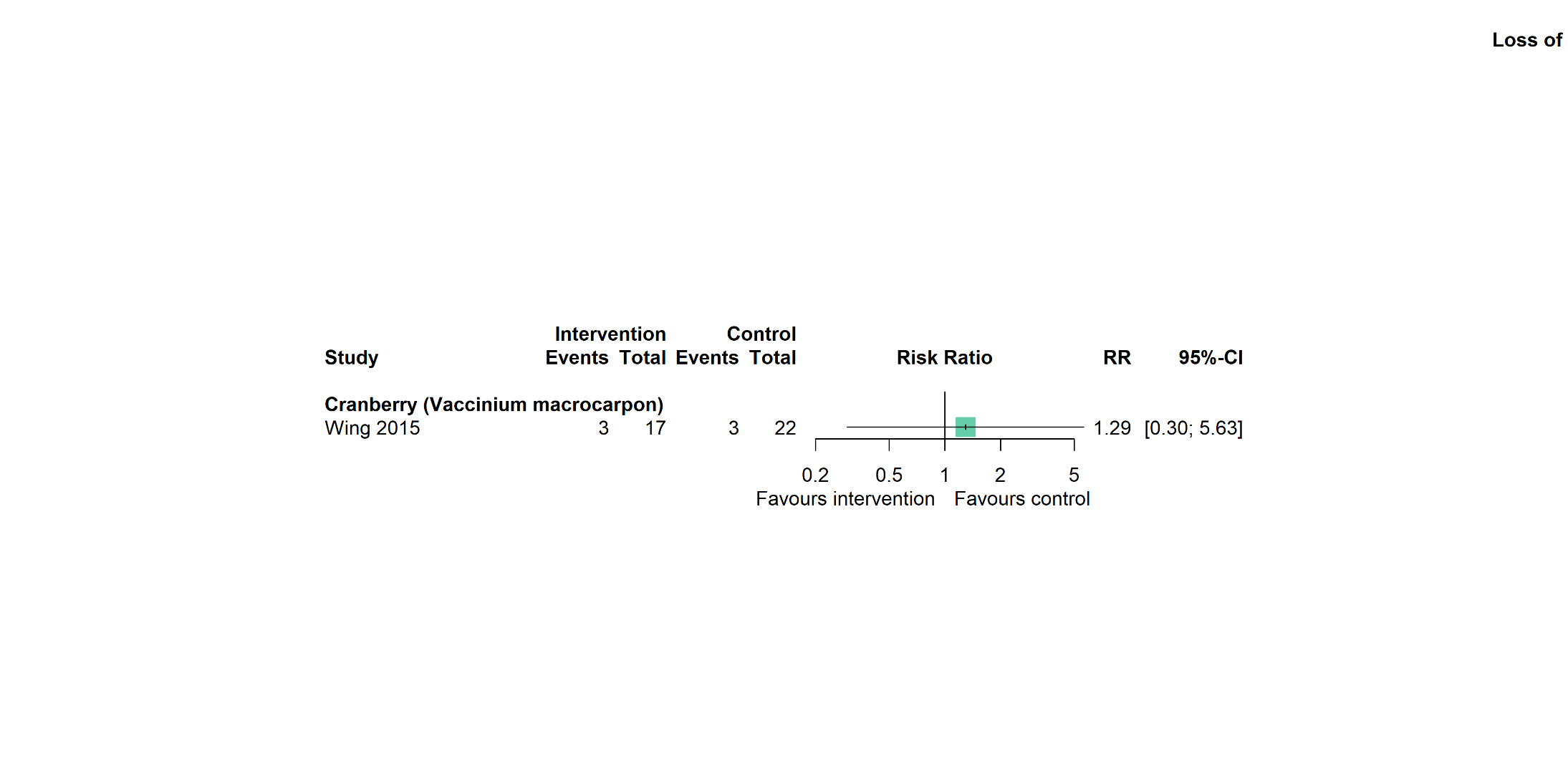
**

- 1. **Side effects – Nausea**

**
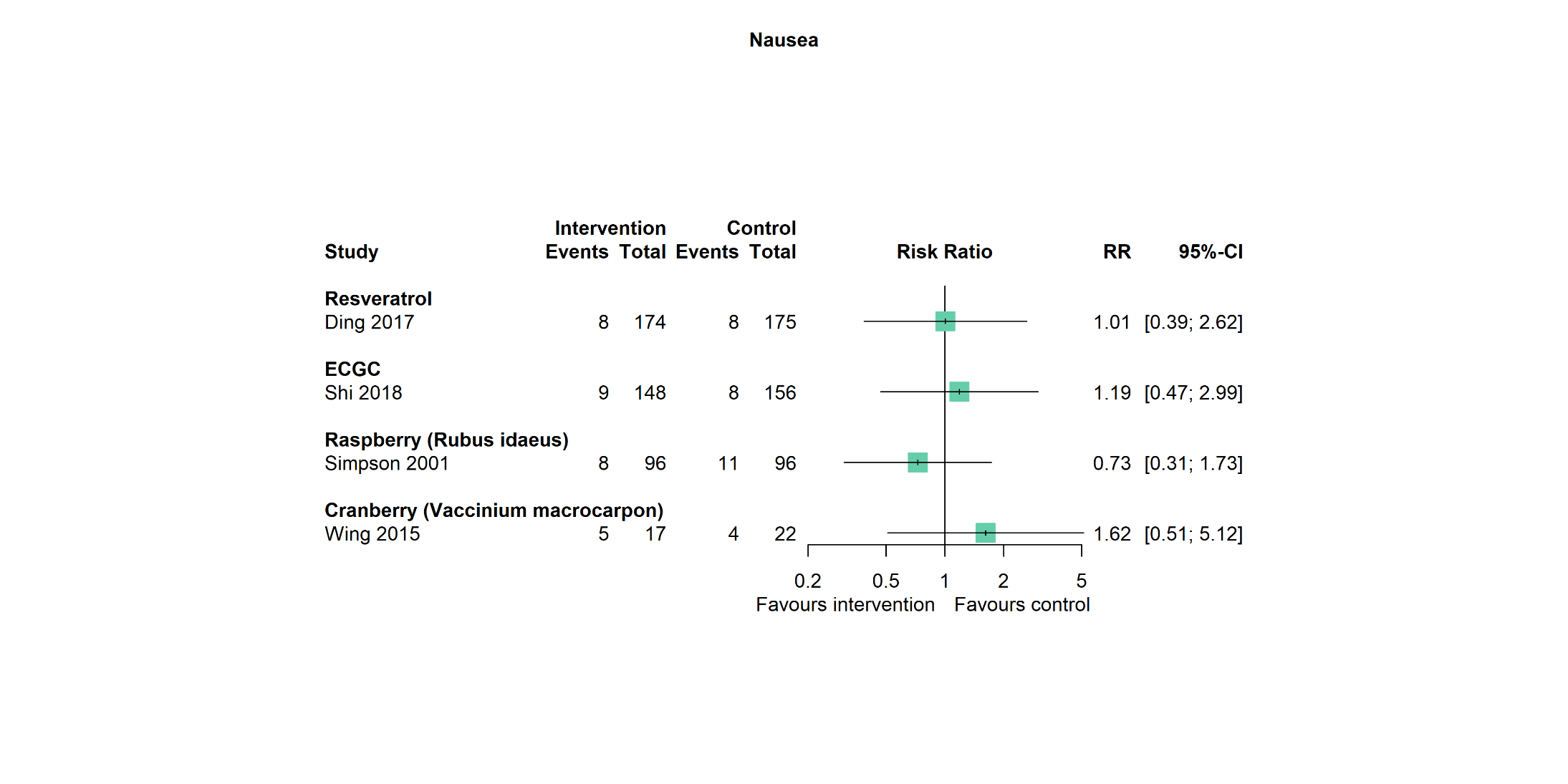
**

- 1. **Side effects – Rash**

**
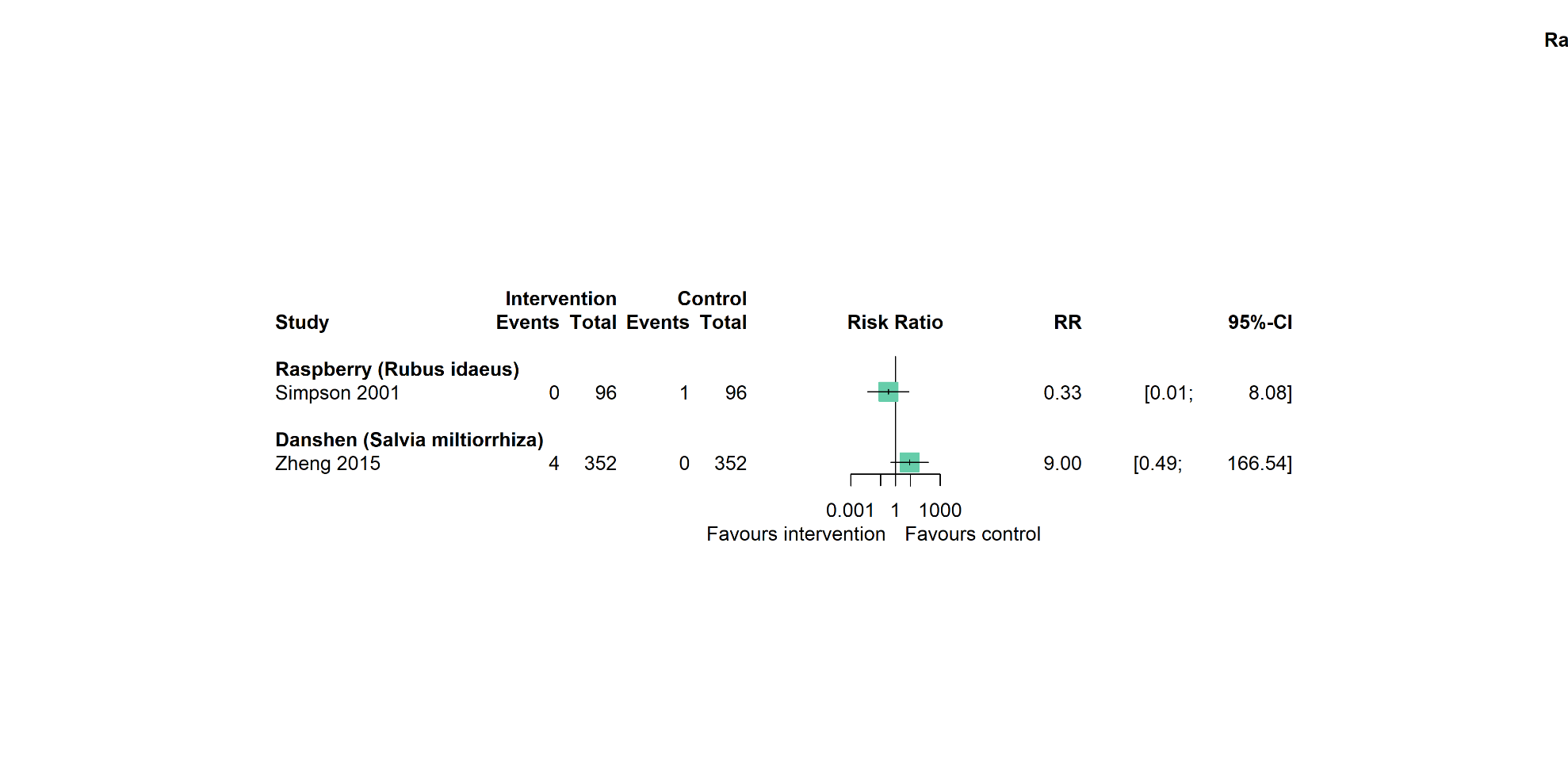
**

- 1. **Side effects – Shortness of breath**

**
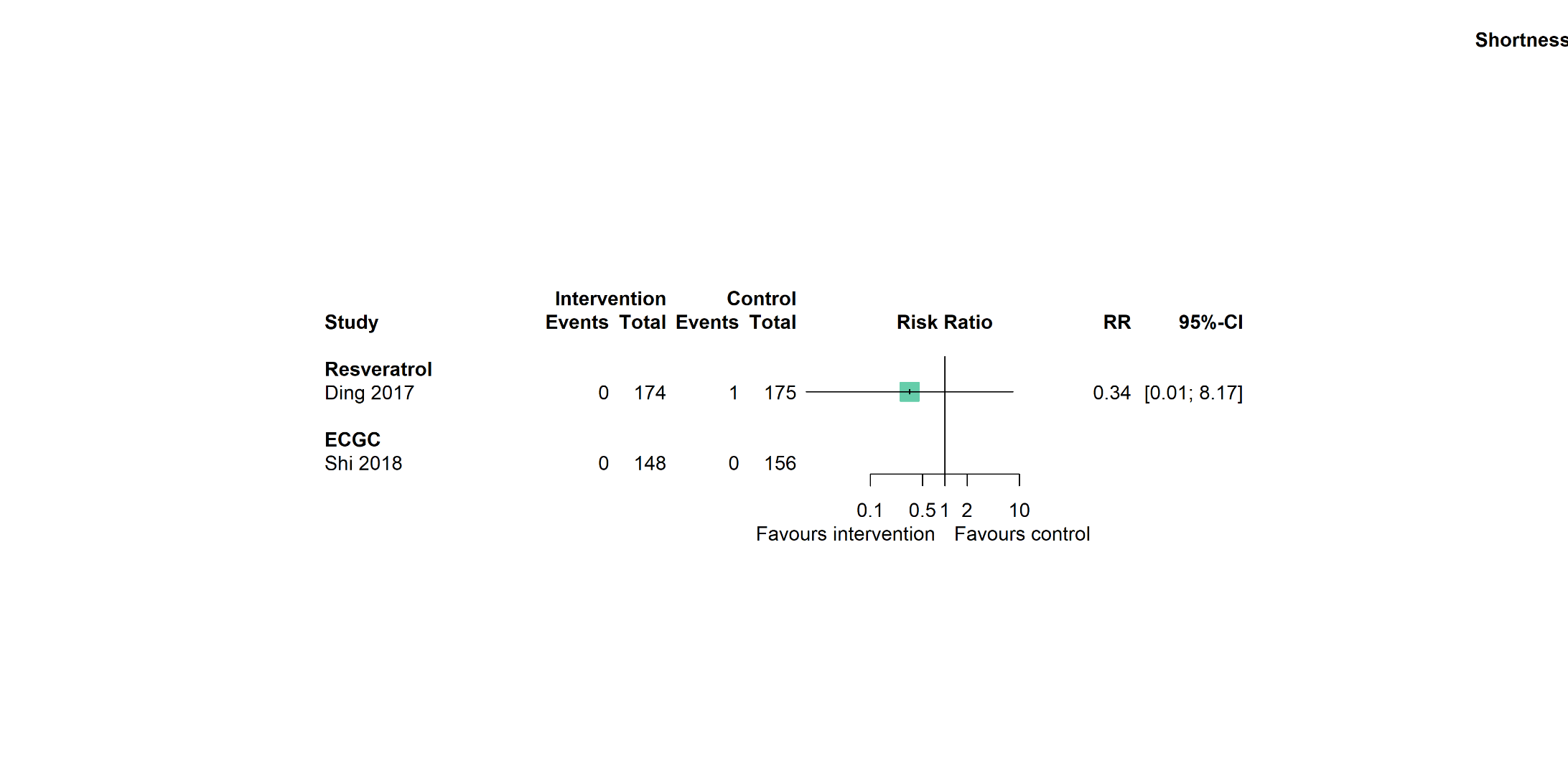
**

- 1. **Side effects – Stomach pain**

**
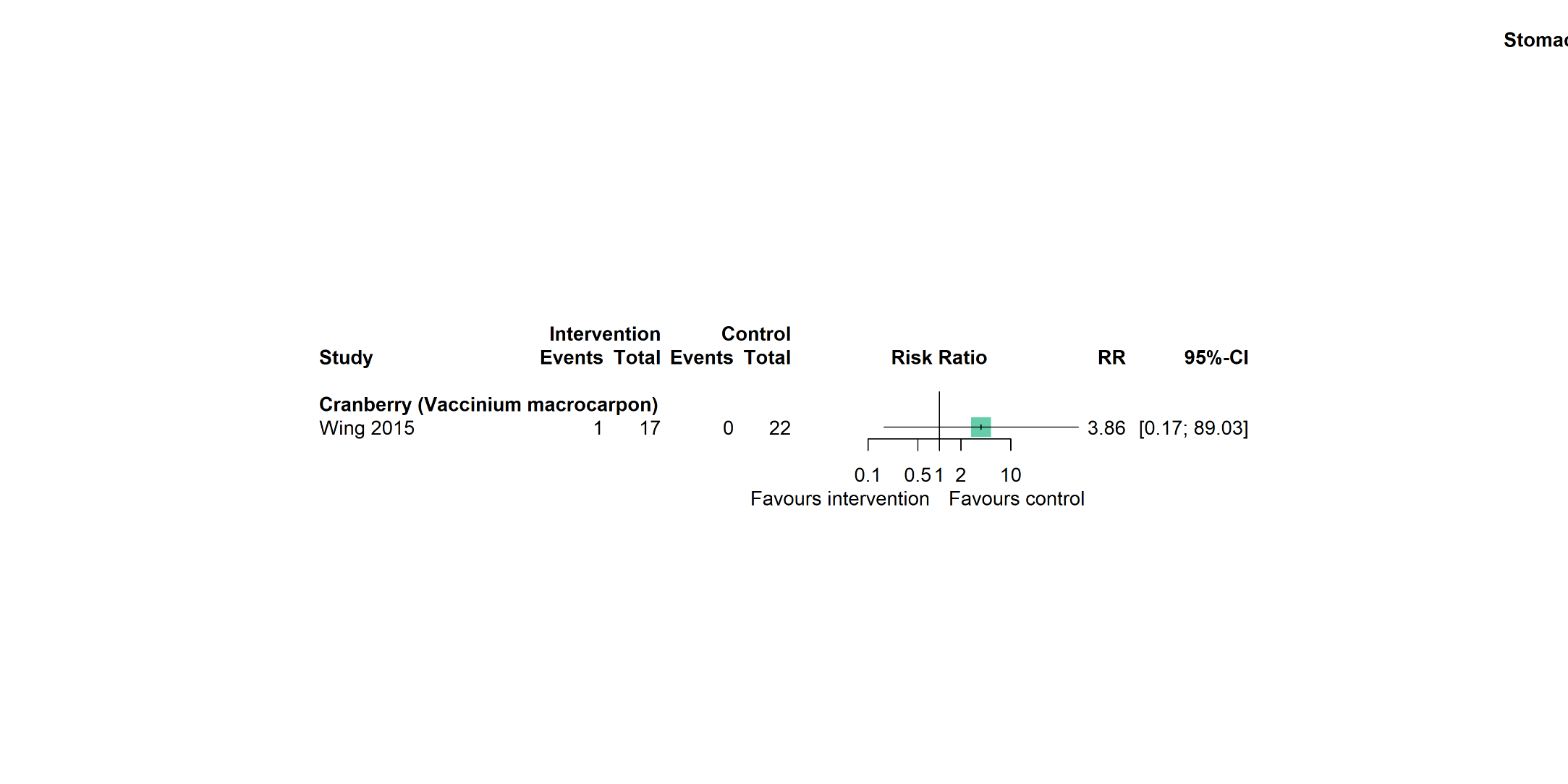
**

- 1. **Side effects – Tachycardia**

**
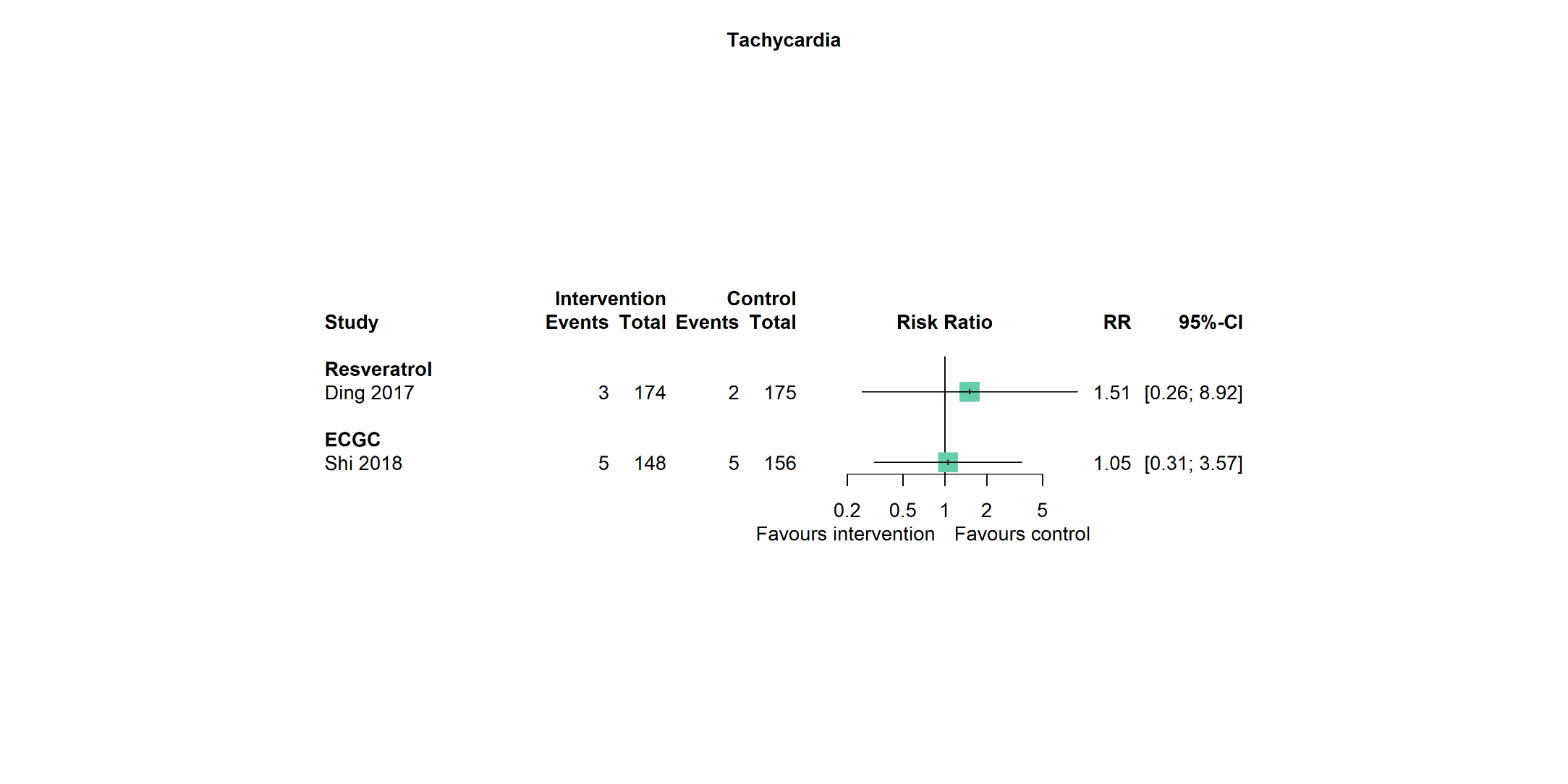
**

- 1. **Side effects – Uterine tightening**

**
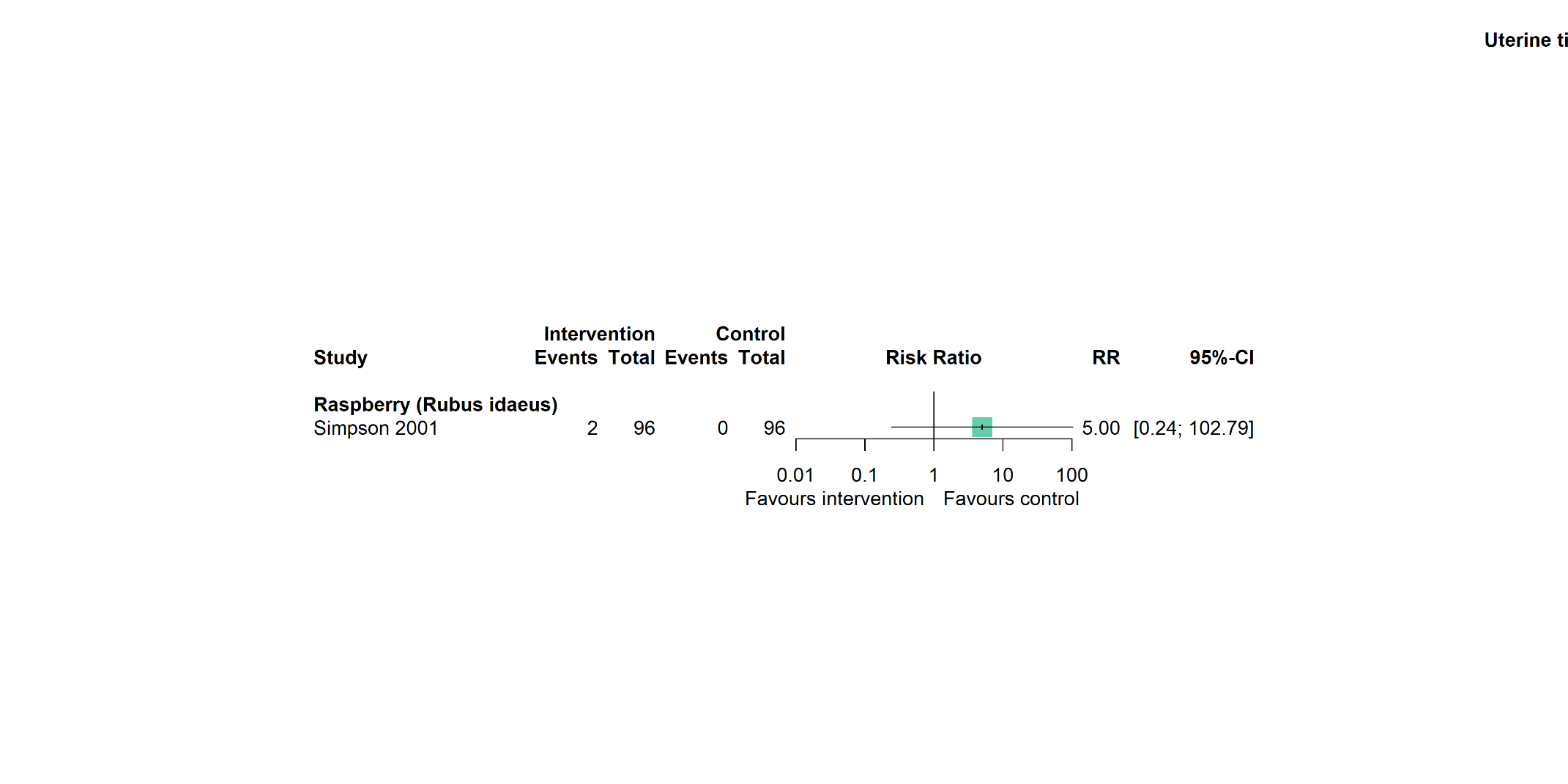
**

- 1. **Side effects – Vomiting**

**
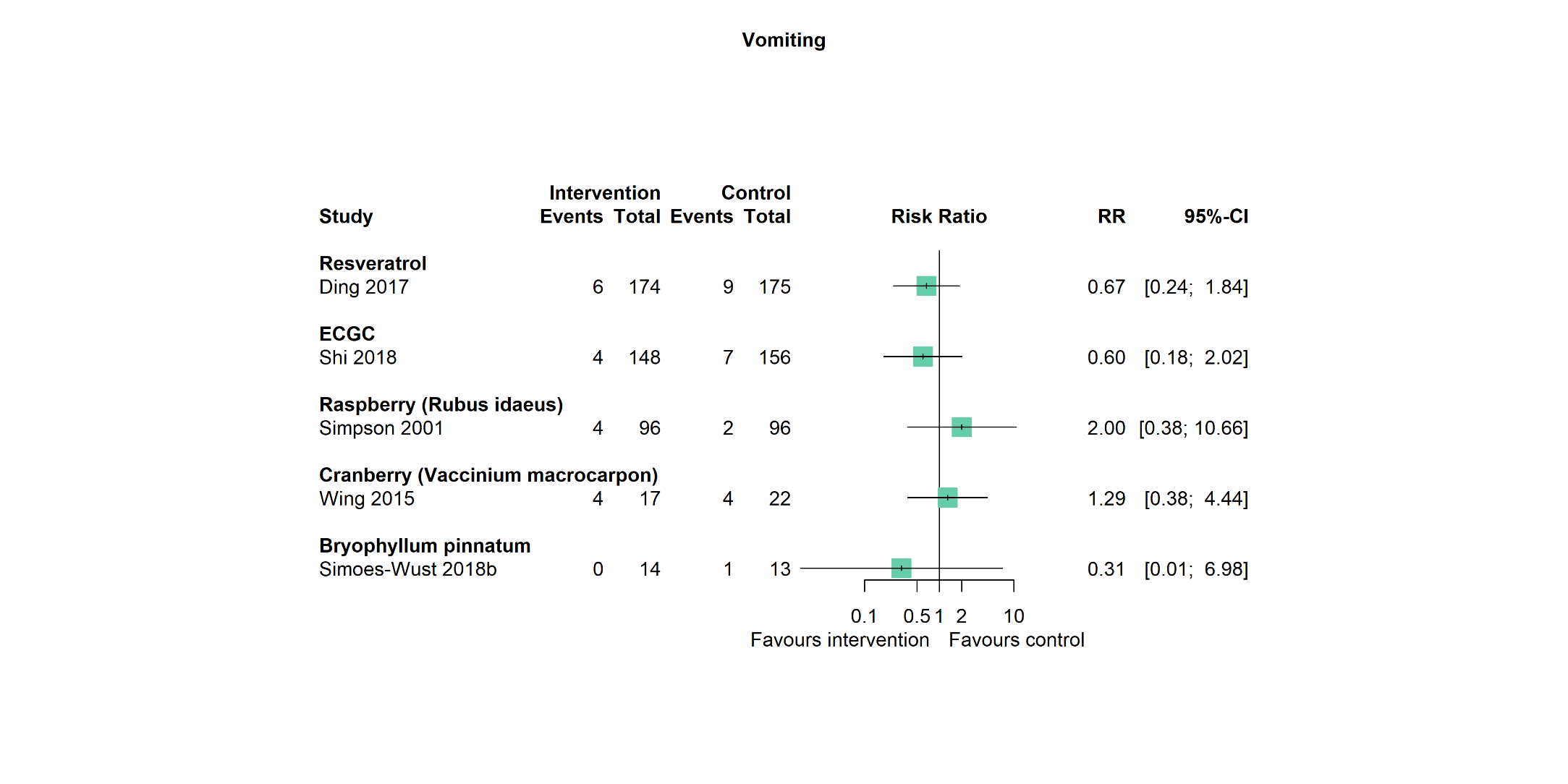
**

- 1. **Side effects – Hypotension**

**
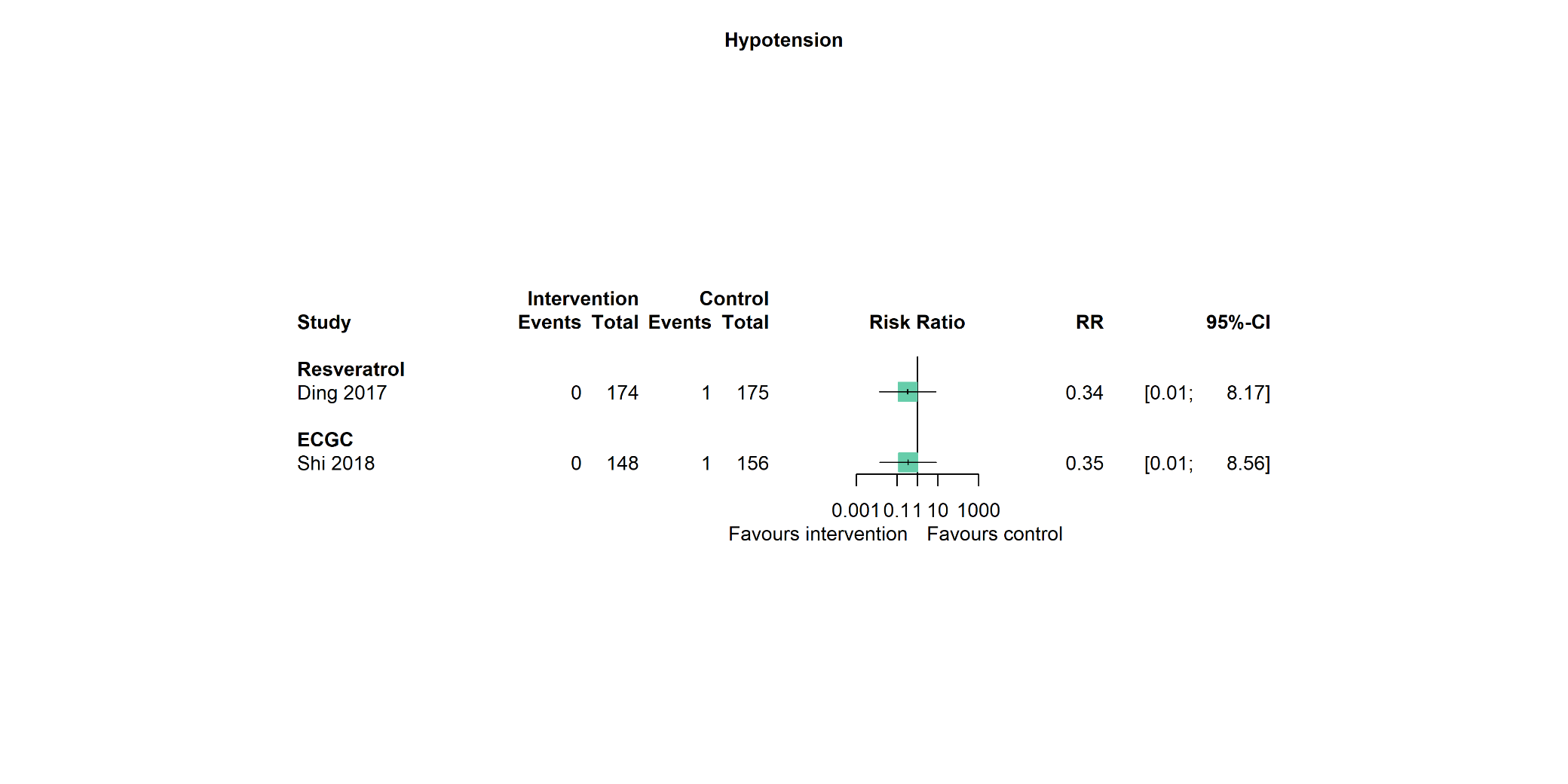
**

- 1. **Prostaglandin I2 (PGI2) (pg/L)**


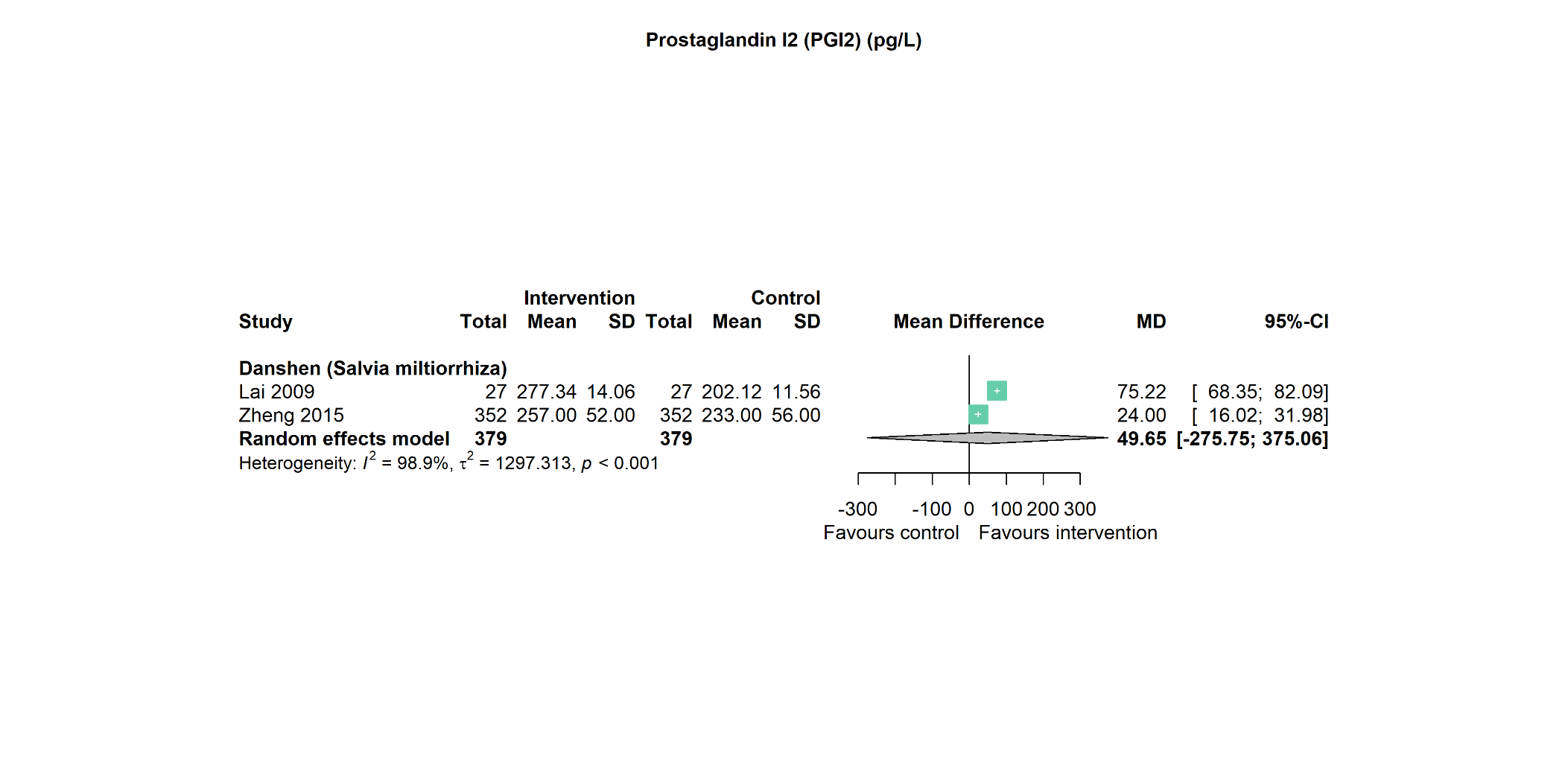


- 1. **Endothelin-1 (ET-1) (ng/L - venous blood; mg/L - serum)**

**
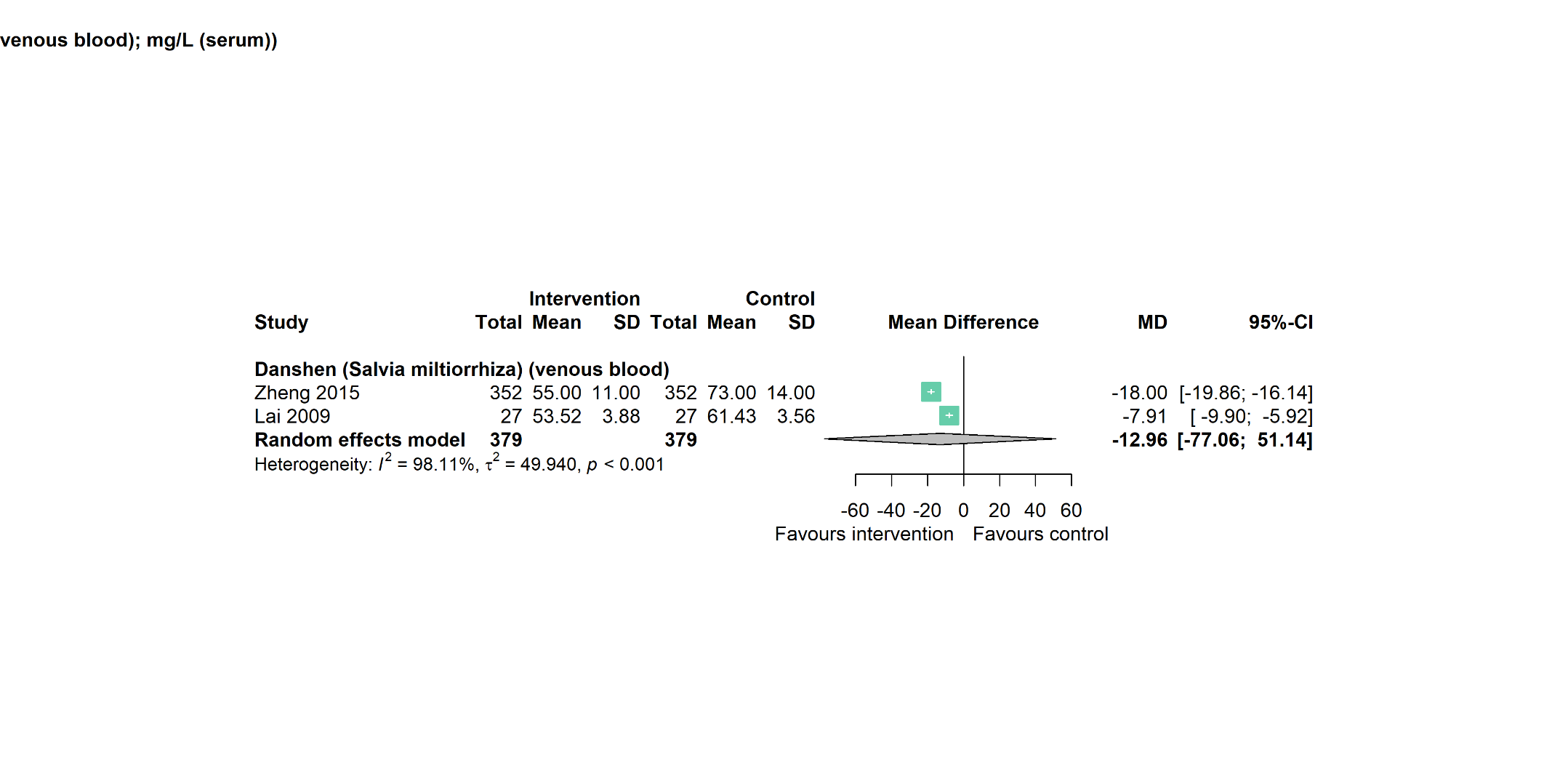
**

**Reference**

1. McDougall ARA, Hastie R, Goldstein M, Tuttle A, Tong S, Ammerdorffer A, et al. Systematic evaluation of the pre-eclampsia drugs, dietary supplements and biologicals pipeline using target product profiles. BMC Medicine. 2022 Nov 4;20(1):393.

2. Zhu L, Zhang Y, Liu Y, Zhang R, Wu Y, Huang Y, et al. Maternal and Live-birth Outcomes of Pregnancies following Assisted Reproductive Technology: A Retrospective Cohort Study. Sci Rep. 2016 Oct 20;6(1):35141.

3. Messerlian C, Maclagan L, Basso O. Infertility and the risk of adverse pregnancy outcomes: a systematic review and meta-analysis. Hum Reprod. 2013 Jan;28(1):125–37.

4. Weibel S, Popp M, Reis S, Skoetz N, Garner P, Sydenham E. Identifying and managing problematic trials: a Research Integrity Assessment (RIA) tool for randomized controlled trials in evidence synthesis. Research synthesis methods. 2022;

5. Schulz A, Schürmann C, Skipka G, Bender R. Performing Meta-analyses with Very Few Studies. Methods Mol Biol. 2022;2345:91–102.

6. Böhning D, Malzahn U, Dietz E, Schlattmann P, Viwatwongkasem C, Biggeri A. Some general points in estimating heterogeneity variance with the DerSimonian-Laird estimator. Biostatistics. 2002 Dec;3(4):445–57.

7. Cornell JE, Mulrow CD, Localio R, Stack CB, Meibohm AR, Guallar E, et al. Random-effects meta-analysis of inconsistent effects: a time for change. Ann Intern Med. 2014 Feb 18;160(4):267–70.

8. IntHout J, Ioannidis JP, Borm GF. The Hartung-Knapp-Sidik-Jonkman method for random effects meta-analysis is straightforward and considerably outperforms the standard DerSimonian-Laird method. BMC Med Res Methodol. 2014 Feb 18;14:25.

9. Andira A, Hadju V, Ariyandi A. The Effect Of Extract Moringa Oleifera Leaves Plus Royal Jelly On Hematocrit Level Of Anaemic Pregnant Women In Takalar District. Eur J Mol Clin Med. 2020;7(6).

10. Arundhana AI, Nurdin MS, Hadju V, Ansariadi, Zulkifli A. The Effect of Moringa-Based Supplementation on Fetal Birth Weight in Jeneponto Regency. Journal of Pharmacy and Nutrition Sciences. 2018 Jul 5;8(3):144–9.

11. Basri H, Hadju V, Zulkifli A, Syam A, Indriasari R. Effect of Moringa oleifera supplementation during pregnancy on the prevention of stunted growth in children between the ages of 36 to 42 months. J Public Health Res. 2021 Apr 14;10(2):2207.

12. Chen D jin, Zhao Y, Sheng R jing. Effect of Salvia Miltiorrhiza, Ligustrazine and Magnesium Sulfate on Outcomes of Patients with Pregnancy-induced Hypertension. Chinese Journal of Perinatal Medicine [Internet]. 2003 [cited 2023 Jun 5]; Available from: https://www.semanticscholar.org/paper/Effect-of-Salvia-Miltiorrhiza%2C-Ligustrazine-and-on-Dun-jin-Yang/9ab9410f1fe13bc93c0ba603502a7a3f69f83de8

13. Hastuty D, Hadju V, Ariyandy A. The Effect Of Giving Extracted Moringa Oleifera Leaves Plus Royal Jelly Supplement On Erythrocyte Index Of Anemia Pregnant Women In Takalar District. European Journal of Molecular & Clinical Medicine. 2020 Nov 25;7(6):701–10.

14. Li C fang, Gou W li, Han Z. [Investigation on treatment of fetal growth restriction by salvia injection combined with composite amino acid]. Zhongguo Zhong Xi Yi Jie He Za Zhi. 2009 Jan;29(1):68–71.

15. Mandasari M, Hadju V, Ariyandy A. The effect of giving extracted moringa oleifera leaves plus royal jelly supplement on infant weight and length of new born of anemia pregnant woman in Takalar district. European Journal of Molecular and Clinical Medicine. 2020 Sep 1;7:4952–61.

16. Baghbahadorani FK, Miraj S. The impact of Silymarin on improvement of platelet abnormalities in patients with severe preeclampsia. Electron Physician. 2016 May 25;8(5):2436–42.

17. Nadimin, Hadju V, As’ad S, Buchari A, Haruna I, Hartono R. Increasing of Nutrition Status of Pregnant Women after Supplementation of Moringa Leaf Extract (Moringa Oliefera) in the Coastal Area of Makassar, Indonesia. Ind Jour of Publ Health Rese & Develop. 2019;10(1):521.

18. Nadimin, Hadju V, As’ad S, Bukhari A, Arundhana AI, Imrawati. A comparison between extract Moringa oleifera and iron tablet on prevention low birth weight in pregnant mothers in Makassar, Indonesia. Enferm Clin. 2020 Jun 1;30:26–30.

19. Pawłowicz P, Wilczyński J, Stachowiak G, Hincz P. [Administration of natural anthocyanins derived from chokeberry retardation of idiopathic and preeclamptic origin. Influence on metabolism of plasma oxidized lipoproteins: the role of autoantibodies to oxidized low density lipoproteins]. Ginekol Pol. 2000 Aug;71(8):848–53.

20. Shen F, Lv H, Shi Y, Lau S, Guo F, Chen Q. A pilot study on investigating the role of Salvia miltiorrhiza in fetal growth restriction. Biosci Rep. 2020 Jun 26;40(6):BSR20201222.

21. Zakiah V, Hadju V, Ariyandy A. The Effect Of Giving The Supplement Of Kelor Leaves (Moringa Oleifera Leaves) Plus Royal Jelly To Malondialdehid Levels In Anemic Pregnant Women In Takalar District. Eur J Mol Clin Med. 2020;7(6).

22. Zhao X, Wang H, Gao Y, Wang Y. Effects of Compound Danshen Injection Combined with Magnesium Sulfate on Pregnancy-Induced Hypertension Syndrome under the Guidance of Empirical Mode Decomposition Algorithm-Based Ultrasound Image. J Healthc Eng. 2021;2021:9026223.

23. Zhou Y, Wang J, Wang L, Tang J, Zhang C. Effect of Compound Danshen Injection Combined with Magnesium Sulfate on Oxidative Stress, TNF-α, NO, and Therapeutic Efficacy in Severe Preeclampsia. Comput Intell Neurosci. 2022 Jul 18;2022:9789066.

24. El-Aal DEMA, Ali MK, Shahin AY, Zakherah MS, Abdellah MS, Abdel-Kawi AF, et al. Effects of oral ginkgo biloba extract on pregnancy complicated by asymmetrically intrauterine growth restriction: a double-blinded randomized placebo-controlled trial. Proceedings in Obstetrics and Gynecology [Internet]. 2017 Mar 1 [cited 2023 Jul 23];7(1). Available from: https://pubs.lib.uiowa.edu/pog/article/id/3545/

25. Chu HN, Shen MJ. Treating oligohydramnios with extract of Salvia miltiorrhiza: A randomized control trial. Ther Clin Risk Manag. 2008 Feb;4(1):287–90.

26. Fadinie W, Lelo A, Wijaya DW, Lumbanraja SN. Curcumin’s Effect on COX-2 and IL-10 Serum in Preeclampsia’s Patient Undergo Sectio Caesarea with Spinal Anesthesia. Open Access Maced J Med Sci. 2019 Oct 30;7(20):3376–9.

27. Fadinie W, Lelo A, Wijaya DW, Lumbanraja SN. Curcumin And Its Effect On Preeclampsia: As Anti-Inflammatory, Analgesic, And Anticoagulant. Int J Curr Pharm Sci. 2020 Mar 17;43–7.

28. Hekmatzadeh SF, Bazarganipour F, Malekzadeh J, Goodarzi F, Aramesh S. A randomized clinical trial of the efficacy of applying a simple protocol of boiled Anethum Graveolens seeds on pain intensity and duration of labor stages. Complement Ther Med. 2014 Dec;22(6):970–6.

29. Ma H, Qiao Z. Analysis of the efficacy of resveratrol treatment in patients with scarred uterus. Exp Ther Med. 2018 Jun;15(6):5410–4.

30. Sulistyowati D, Soejoenoes A, Hadisaputro S, Sujianto U. Effectiveness Of Nano Particles Of Moringa Oleifera Leaf Extract On Anxiety, Serotonin Levels In Pregnant Women With Hypertension. Neuroquantology. 2022 Nov;20(17):1110–4.
